# Genome-Wide Association and Transcriptome Analyses Reveal Candidate Genes Underlying Yield-determining Traits in *Brassica napus*

**DOI:** 10.3389/fpls.2017.00206

**Published:** 2017-02-15

**Authors:** Kun Lu, Liu Peng, Chao Zhang, Junhua Lu, Bo Yang, Zhongchun Xiao, Ying Liang, Xingfu Xu, Cunmin Qu, Kai Zhang, Liezhao Liu, Qinlong Zhu, Minglian Fu, Xiaoyan Yuan, Jiana Li

**Affiliations:** ^1^College of Agronomy and Biotechnology, Southwest UniversityChongqing, China; ^2^School of Management, Xihua UniversityChengdu, China; ^3^Oil Research Institute of Guizhou Province, Guizhou Academy of Agricultural SciencesGuiyang, China; ^4^College of Life Sciences, South China Agricultural UniversityGuangzhou, China; ^5^Industrial Crops Institute, Yunnan Academy of Agricultural SciencesKunming, China

**Keywords:** *Brassica napus*, yield-determining traits, genome-wide association study, transcriptome sequencing, candidate genes

## Abstract

Yield is one of the most important yet complex crop traits. To improve our understanding of the genetic basis of yield establishment, and to identify candidate genes responsible for yield improvement in *Brassica napus*, we performed genome-wide association studies (GWAS) for seven yield-determining traits [main inflorescence pod number (MIPN), branch pod number (BPN), pod number per plant (PNP), seed number per pod (SPP), thousand seed weight, main inflorescence yield (MIY), and branch yield], using data from 520 diverse *B. napus* accessions from two different yield environments. In total, we detected 128 significant single nucleotide polymorphisms (SNPs), 93 of which were revealed as novel by integrative analysis. A combination of GWAS and transcriptome sequencing on 21 haplotype blocks from samples pooled by four extremely high-yielding or low-yielding accessions revealed the differential expression of 14 crucial candiate genes (such as *Bna.MYB83, Bna.SPL5*, and *Bna.ROP3*) associated with multiple traits or containing multiple SNPs associated with the same trait. Functional annotation and expression pattern analyses further demonstrated that these 14 candiate genes might be important in developmental processes and biomass accumulation, thus affecting the yield establishment of *B. napus*. These results provide valuable information for understanding the genetic mechanisms underlying the establishment of high yield in *B. napus*, and lay the foundation for developing high-yielding *B. napus* varieties.

## Introduction

Yield is one of the most complex and important crop traits (Shi et al., 2009). An end product of the interaction between genotypes and the environment during the course of crop growth and development. Yield is determined by the yield-determining traits (YDTs), including direct effects of yield component traits (YCTs) and indirect effects of yield-related traits (YRTs), which have been validated in different crops (Yin et al., 2002; Liu et al., 2008; Cai et al., 2014). As the second largest oilseed crop in the world, *Brassica napus* (rapeseed, oilseed rape or canola) was formed from recursive and independent hybridizations between *Brassica rapa* (A genome) and *Brassica oleracea* (C genome) diploid species. For *B. napus*, seed yield (SY) is mainly determined by three YCTs: silique number (SN), seed number per silique (SPS), and thousand seed weight (TSW; Özer et al., 1999). YRTs such as plant height (PH), first branch height (FBH), inflorescence length (IL), silique length (SL), seed density (SD), silique breadth (SB), silique thickness (ST), and silique volume (SV) also influence SY by affecting the YCTs in *B. napus* (Quijada et al., 2006; Chen et al., 2007; Shi et al., 2009; Cai et al., 2014; Wang X. et al., 2016). Therefore, investigating the genetic basis of YDTs will improve our understanding of yield establishment in *B. napus*, and may be used in high-yield *B. napus* breeding programs.

Quantitative trait locus (QTL) mapping based on molecular markers is widely used to genetically analyze agronomically important traits in *B. napus* (Quijada et al., 2006; Udall et al., 2006; Chen et al., 2007, 2011; Shi et al., 2009; Yan et al., 2009; Yang et al., 2011; Ding et al., 2012). Recently, QTL mapping studies have shed light on the genetic architecture of YDTs in *B. napus*, and so far, hundreds of QTLs have been identified to have significant associations with YDTs in different bi-parental populations (Zhao et al., 2016). Li et al. (2007) constructed a genetic linkage map using an F_2_ population, and identified 133 QTLs for 12 yield traits (including SN, TSW and SPS, etc.), including 14 consistent ones across the two trail locations. They also found that most of the QTLs were clustered, especially on linkage groups (LGs) N2 and N7. Radoev et al. (2008), employing a doubled-haploid (DH) lines and their corresponding testcrosses at four locations, identified 33 QTLs for SY and three YCTs, of which 10 showed significant dominance effects. Shi et al. (2009) identified 85 QTLs for SY along with 785 QTLs for eight yield-associated traits (such as seed-number, seed-weight, biomass-yield, etc.) based on the analysis of two (TNDH and RC-F_2_) populations in 10 natural environments. Yang et al. (2012) identified a major QTL (*cqSWA9*) for SL and seed weight (SW), in two environments, which explained as much as 28.2% of the total SW variation. And this QTL was validated by Fu et al. (2015) in another DH and DH-derived reconstructed F_2_ populations. By fine mapping and association analysis, Liu et al. (2015) cloned the first QTL in polyploidy crops at the same region, and revealed that a 165-bp deletion in auxin-response factor 18 (*ARF18*) is associated with increased SW and SL.

Recently, these QTLs have been further integrated into consensus QTLs according to high-density consensus linkage maps constructed from different studies, using meta-analysis method. Zhou et al. (2014) carried out *in silico* integration of 1960 QTLs associated with 13 SY and YRTs from 15 *B. napus* mapping studies, and mapped 736 QTLs onto 283 loci in the A and C genomes of *B. napus*. Zhao et al. (2016) constructed a high-density consensus map and integrated 226 QTLs for SY and YRTs into 144 consensus QTLs, which was further integrated into 72 pleiotropic unique QTLs by trait-by-trait meta-analysis. Several candidate genes controlling YDTs in the consensus QTL regions were also observed based on comparative mapping among *Arabidopsis, B. rapa, B. oleracea*, and *B. napus*, including five each for SY (*GASA4, CLH1, RBCS1A, LQY1*, and *GGH1*) and TSW (*PTH2, AP2, LCR64, LCR65*, and *PDF1*; Zhao et al., 2016). However, the majority of consensus QTLs were environment specific, 70.8 and 23.6% of QTLs were only detected in winter and spring regions, respectively. Only a few QTLs (5.6%) were detected in both regions (Zhao et al., 2016), which present a barrier to apply QTLs associated with YDTs to marker-based breeding efforts aimed at developing high-yield *B. napus* varieties.

Genome-wide association studies (GWAS) were used first to identify loci associated with complex human diseases (Donnelly, 2008). With improvements in sequencing technologies, and the reduction in the cost of sequencing and genotyping, this method is widely applied to unravel complex quantitative traits in many crops, especially those for which genome sequences are available (Clark, 2010; Huang et al., 2010; Tian et al., 2010; Huang and Han, 2014; Zhou et al., 2015). The recent release of the genome sequences for *B. napus* and its parental species *B. rapa* and *B. oleracea* (Wang et al., 2011; Chalhoub et al., 2014; Liu et al., 2014; Parkin et al., 2014), and the development of high-density customized single nucleotide polymorphism (SNP) arrays (i.e., the *Brassica* 60K Illumina Infinium SNP array, and the *B. napus* 6K Illumina Infinium SNP array) provided the *Brassica* scientific community with powerful tools for unraveling the genetic architecture of important traits and identifying the loci and candidate genes underlying traits of interest in *Brassica* crops (Li et al., 2014; Körber et al., 2016). Based on *Brassica* 60 K SNP array, 9 and 312 SNPs have been identified to be significantly associated with harvest index and flowering time in *B. napus*, respectively (Luo et al., 2015; Xu et al., 2015; Wang N. et al., 2016). In addition, a total of 50, 25, and 8 loci were detected to be associated with seed oil content, branch angle and PH in different *B. napus* natural populations, respectively (Li F. et al., 2016; Liu J. et al., 2016; Liu S. et al., 2016; Sun et al., 2016). For SY and YDTs, Schiessl et al. performed a GWAS with a 60 K SNP array for a panel of 158 European winter-type accessions grown in highly diverse field environments, and identified 36 genome regions associating with SY in *B. napus* (Schiessl et al., 2015). Cai et al. conducted a GWAS for 6 YDTs and detected 7 and 9 associated markers for SPS and TSW using a panel of 192 inbred lines of *B. napus*, which was genotyped using 451 single-locus microsatellite markers and 740 amplified fragment length polymorphism markers (Cai et al., 2014). However, GWAS has not yet been used to systematically and separately elucidate the genetic basis of YDTs on the main inflorescence and branches of *B. napus*.

To extend our knowledge of the genetic control of YDTs, and to identify candidate genes to improve *B. napus* yield, we conducted GWAS on an association panel of 520 *B. napus* accessions grown in two different yield conditions. Yunnan (YN) was chosen to represent a typical high-yielding Chinese production region, with average yields of over 5.3 tons per hectare, and Chongqing (CQ) was chosen to represent a standard production region, with an average yield of about 2.7 tons per hectare (Zhang et al., 2012; Cai et al., 2016; Lu et al., 2016). We also sequenced the transcriptomes of eight tissues from high-yield and low-yield *B. napus* accessions from the two aforementioned regions. Finally, differentially expressed candidate genes were selected from haplotype blocks (HBs) of significant loci affecting the YDTs. These results provide insight into the genetic basis underlying the establishment of high yield in *B. napus*, and lay the foundation for marker-based breeding efforts to develop high-yield varieties.

## Materials and methods

### Plant materials and field trails

Detailed information on the 520 diverse *B. napus* accessions and genotypes are described in our previous study (Lu et al., 2016). All *B. napus* accessions were grown in a randomized block design with three replications in three natural environments at two locations. The three natural environments were E1 (Chongqing, CQ; 29°45′ N, 106° 22′ E, 238.57 m) in the 2012–13 growing seasons, and E2 (CQ) and E3 (Yunnan, YN; 23° 43′ N, 100° 02′ E, 1819.50 m) in the 2013–14 growing season. Each accession was grown in a plot with three rows, 10 plants per row, with a distance of 20 cm between plants in each row and 30 cm between rows. Meteorological data for the two sites during cultivation are listed in Supplementary Table 1.

### Measurement of yield-determining traits

Using the Biologische Bundesanstalt, Bundessortenamt, and CHemical (BBCH) industry scale (Lancashire et al., 1991), five representative plants from the middle of each plot were harvested at growth stage 89 (fully ripe), and seven YDTs were measured, including main inflorescence pod number (MIPN), branch pod number (BPN), pod number per plant (PNP), seed number per pod (SPP), TSW (g), main inflorescence yield (MIY, g/plant), and branch yield (BY, g/plant). TSW was the average dry weight in grams of 1,000 well-filled seeds mixed from five sampled plants. MIPN and MIY were the number of effective pods and seed yield on the main inflorescence of each harvested individual, respectively. BPN and BY were the number of effective pods and seed yield on the secondary and tertiary branches of each harvested individual, respectively. PNP and SPP were measured as effective pod number per plant and seed number per pod, respectively. All the traits investigated in this study, and corresponding measurement method, cultivation environments and unit were summarized in the Supplementary Table 2.

A virtual environment (E4) was used to identify significant signals underlying environmental variation in YTRs. Trait value in E4 was calculated according to the following formula: 2 × (E3 − E2)/(E3 + E2).

### Statistical analysis

Descriptive statistics, analysis of variance (ANOVA) and correlation analysis of YDTs were performed using SAS version 8.0 (SAS Institute Inc.). Broad-sense heritability was calculated as:

$$\begin{array}{l} {\text{h}}^{2}=\sigma_{g}^{2}/\left(\sigma_{g}^{2}+\sigma_{ge}^{2}/E+\sigma_{\varepsilon }^{2}/ER\right) \end{array}$$

Where: $\sigma_{g}^{2}$, $\sigma_{ge}^{2}$, and $\sigma_{\varepsilon }^{2}$ are estimates of the variances of genotype, genotype × environment interactions, and error, respectively. *E* is the number of environments, and *R* is the number of replications per environment (Kowles, 2001).

The ANOVA model was as follows:

$$\begin{array}{l} y_{ger}=\mu +\alpha_{g}+\beta_{e}+{\left(\alpha \beta \right)}_{ge}+\gamma_{r\left(e\right)}+\varepsilon_{ger} \end{array}$$

Where: *y*_*ger*_ was the phenotypic trait value of the *g*th genotype in the *e*th environment for the *r*th replicate, μ the grand mean, α_*g*_ the effect of the *g*th genotype, β_*e*_ the main effect of the *e*th environment, (αβ)_*ge*_ the interaction effect of the *g*th genotype and the *e*th environment, γ_*r*(*e*)_ the block effect within the *e*th environment, and ε_*ger*_ the residual (Freund and Littell, 1981). All effects were treated as random.

### Genotyping, quality control, and location of SNPs

The genotype of the association panel was assayed using a *Brassica* 60K Illumina Infinium SNP array, according to the manufacturer's protocol (Infinium HD Assay Ultra Protocol Guide; Li et al., 2014). SNP alleles were called using GenomeStudio software v2011.1 (Illumina, Inc., San Diego, CA) with the Genotyping module (v1.9.4).

Only SNPs with a percentage of missing data of <10% across all genotypes and a minor allele frequency (MAF) of >0.05 were retained. From 52,157 SNPs in the array, 20,678 SNPs were filtered, and 31,839 were analyzed further. Physical localization of SNPs was assigned using BLASTN searches against the *B. napus* “*Darmor*-bzh” reference genome version 4.1, with an *E*-value cut-off of 1E-5 (Altschul et al., 1997; Chalhoub et al., 2014). Only SNPs with a maximum bit-score were retained as unique SNPs, and subjected to further analysis.

### Population structure and genome-wide association mapping

The population structure (Q) matrix was generated using STRUCTURE 2.3.4 (Pritchard et al., 2000), as reported in our previous study (Lu et al., 2016). In short, three runs were performed for each number of populations (k) varying from 1 to 10, with a burn-in period of 100,000 and 1,000,000 Monte Carlo Markov Chain (MCMC) iterations. The most likely *K*-value was determined by the log probability of the data (LnP(D)) and delta K as proposed by previous study (Evanno et al., 2005). The relative kinship matrix (K) of the association population was calculated using TASSEL 5.2.1 (Bradbury et al., 2007). Linkage disequilibrium (LD) between pairs of SNPs on each chromosome was estimated as the correlation of allele frequencies (*r*^2^) using TASSEL 5.2.1.

Two statistical models were used to test trait–SNP associations. First, the general linear model (GLM) was used, without controlling for population structure (Q) or relative kinship (K), and containing only the SNP that was tested as a fixed effect. Second, the mixed linear model (MLM) was used, controlling for Q and K as fixed and random effects, respectively (Liu S. et al., 2016). Both the GLM and MLM were implemented in TASSEL 5.2.1.

Suggestive (1/*n*) and significant (0.05/*n*) *P*-value thresholds were set to control the genome-wide type 1 error rate derived from MLM and GLM, respectively (Duggal et al., 2008); *n* represented the effective number of independent SNPs calculated using Genetic Type I Error Calculator (GEC) software (Li et al., 2012). Due to the estimated the effective number of independent tests was 12873.97, the *P*-value thresholds used to identify significantly associated SNPs were set at 7.77 × 10−5 [suggestive, −log10(P) = 4.11] and 3.88 × 10−6 [significant, −log10(P) = 5.41]. The Manhattan plot was generated using the R package qqman (Turner, 2014).

To determine the explanatory power of significant SNPs, stepwise regression was applied to estimate the total variance explained by using the R function “stepAIC” in the MASS package (Venables and Ripley, 2002). The best stepwise model was determined according to Akaike information criterion (AIC) values, using the genotypes of significant SNPs as predictor variables in different stepwise models fitted to phenotypic variables. The adjusted *R*^2^ of the best stepwise model was represented as the total variance explained by multiple significant SNPs.

### Transcriptome sequencing and gene ontology (GO) enrichment analysis

Total RNA was extracted from eight tissues of high-SY and low-SY accessions grown at CQ and YN (Supplementary Tables 3, 4). Tissues from mature leaves (Le) and stems (St), and buds on the primary branch (BP) and the main inflorescence (BM), were harvested at BBCH flowering stage 63–65 (Lancashire et al., 1991), according to our previous description (Lu et al., 2015). Seed and silique pericarps on the main inflorescence (SM and SPM, respectively) and on the primary branch (SB and SPB, respectively) were harvested 20 days after flowering. Then, equal amounts of total RNA from four high-SY and low-SY *B. napus* accessions were separately pooled. For each sample, two biological replicates, each obtained from three independent plants, were collected for RNA sequencing (RNA-seq) and quantitative reverse-transcription polymerase chain reaction (qRT-PCR) analysis.

Sixty-four RNA-seq libraries (eight tissues × two different kinds of SY accessions × two environments × two biological replicates per sample) were prepared by the Biomarker Technologies Corporation (Beijing, China) and sequenced using an Illumina HiSeq 2500 sequencer (Illumina Inc., San Diego, CA). Briefly, mRNA was enriched with oligo(dT)-rich magnetic beads and then broken into short fragments using an RNA Fragmentation Kit (Beckman Coulter, Brea, CA, USA). The first- and second-strand cDNAs were synthesized using the cleaved mRNA fragments as templates. After end repair and the addition of single nucleotide A, the sequencing adaptors were ligated to these cDNA fragments. The desired fragments were separated using AMPure XP beads (Beckman Coulter, Brea, CA, USA), and the purified cDNA fragments were enriched through PCR. Finally, the 64 libraries were constructed and sequenced, and 125 bp paired-end reads were generated.

Original RNA-seq data have been deposited in the National Center for Biotechnology Information (NCBI) Short Read Archive under reference number SRP072900. Sequence data quality filtering was performed using Trimmomatic-0.33 (Bolger et al., 2014). Filtered reads were then mapped to the *B. napus* reference genome v4.1 using STAR 2.4.2a (Dobin et al., 2013). Differentially expressed genes (DEGs) were detected using the cuffdiff program, filtered with the following requirements: false discovery rate (FDR, Benjamini–Hochberg multiple test correction) <0.05 and absolute fold change >2 (Benjamini and Hochberg, 1995). To cluster the different RNA-seq samples, principal component analysis (PCA) and cluster analysis were performed as our previously described (Lu et al., 2016).

Gene ontology (GO) terms were assigned to all *B. napus* proteins based on BLASTP analysis against the *Arabidopsis* proteome (TAIR10), with an *E*-value cut-off of 1E-5 (Altschul et al., 1997). BinGO v2.4.4 was used to identify significantly enriched GO terms (FDR < 0.05; Maere et al., 2005). The *P*-values of significantly overrepresented GO terms were corrected for multiple comparisons using the Benjamini–Hochberg method (Benjamini and Hochberg, 1995). Only terms with an FDR of <0.01 were included and visualized using the R package ggplot2 (Ginestet, 2011).

### Candidate gene mining

To identify reliable significant association signals in our GWAS, LD analysis was used to determine the haplotype block (HB) for each significant SNP. Only HBs associated with multiple traits, or containing multiple SNPs associated with the same trait, were used to identify candidate genes. LD was estimated as the correlation coefficient *r*^2^ between all SNP pairs, and calculated using Haploview 4.2 (Barrett et al., 2005). The Four Gamete Rule, with a fourth haplotype frequency cutoff of 0.1, was used to define haplotype blocks (HBs) based on LD. If significant SNPs were located outside of all the HBs, the 100-kb flanking regions on either side of the markers were treated as an HB. Candidate genes regulating YDTs were identified by screening DEGs within HBs.

To identify candidate genes controlling YDTs, results from GWAS and RNA-seq analysis were integrated and, between the high-SY and low-SY accessions within the HBs, only DEGS associated with multiple traits, or containing multiple SNPs associated with the same trait, were chosen for functional annotation based on their *Arabidopsis* orthologs. In each HB, only the most likely candidate gene was selected from DEGs underlying flower or seed developmental processes, cell organization and biogenesis, or transcription factor activity.

### Validation of DEGs by qRT-PCR

Quantitative PCR analysis of differentially expressed (DE) candidate genes was performed as described previously (Lu et al., 2015). Briefly, RNA samples prepared for the aforementioned RNA-seq were used for cDNA synthesis and qRT-PCR detection. Melting curve analysis ranging from 55 to 95°C was used to assess specificity. Two independent biological replicates, each with three technical replicates, were employed for each tested sample and template-free negative controls. Gene-specific primers were designed using Geneious Pro 8.1.5 (Supplementary Table 5; Kearse et al., 2012). Relative quantification was normalized to the *B. napus* housekeeping genes *Bna.UBC21* and *Bna.ACT7*, and a fold-change in gene expression calculation was performed using the 2^−ΔΔ^Ct method (Qu et al., 2015).

## Results

### Phenotypic analysis of YDTs

**Figure 1:**
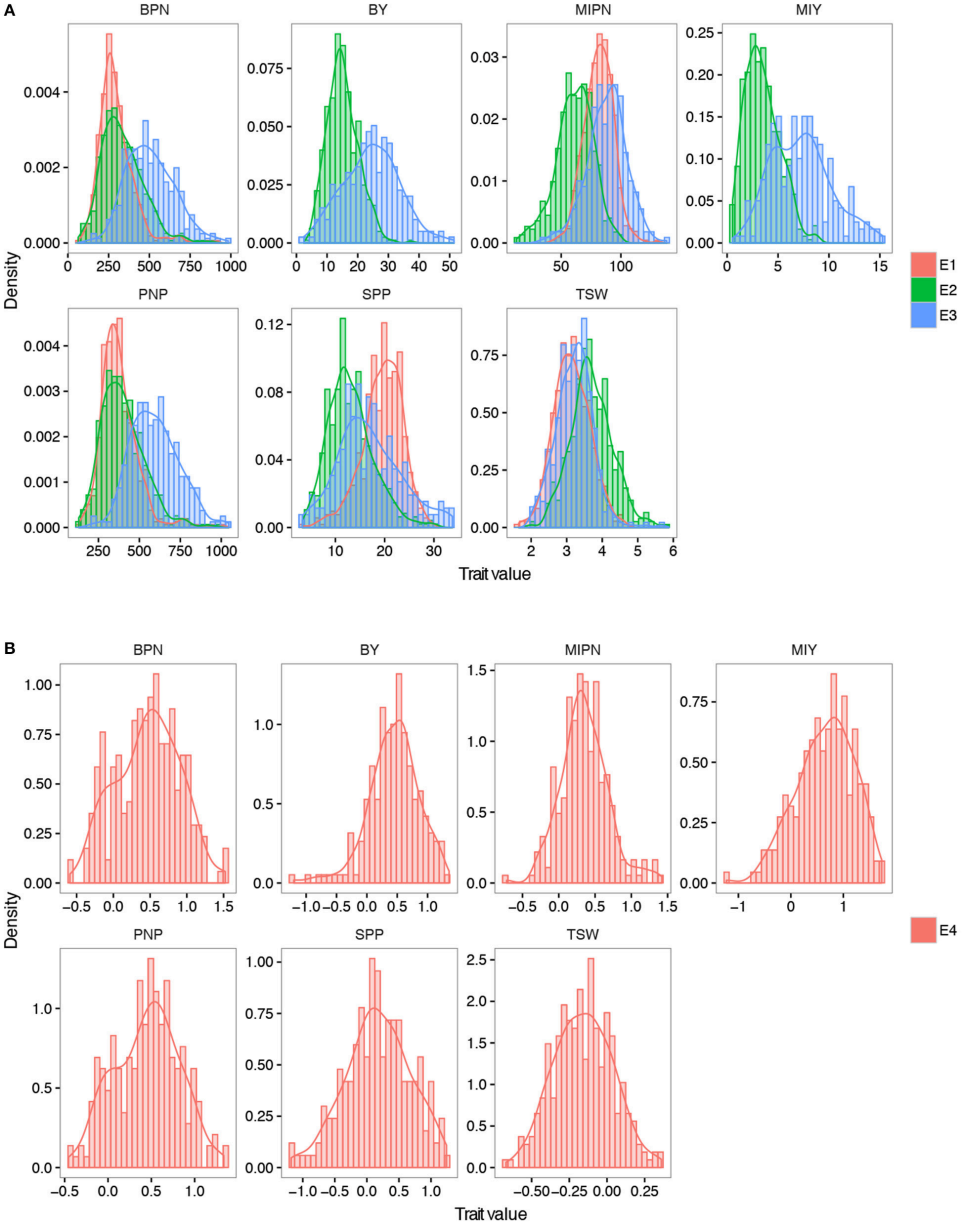
**Frequency distribution of seven YDTs. (A)** Density plot of all the YDTs in three natural environments. **(B)** Density plot of traits in the virtual environments. MIPN, main inflorescence pod number; BPN, branch pod number; PNP, pod number per plant; SPP, seed number per pod; TSW, thousand seed weight; MIY, main inflorescence yield; BY, branch yield. E1, E2, and E3 refer to plants grown in CQ 2013, CQ in 2014, and YN in 2014, respectively. E4 refers to plants grown in a virtual environment. The trait value in E4 was calculated by the formula: 2 × (E3 − E2)/(E3 + E2).

In trait evaluation under three natural environments, descriptive statistics revealed extensive phenotypic variation for seven YDTs: MIPN, BPN, PNP, SPP, TSW, MIY, and BY (Table 1 and Figure 1). TSW was the most stable, ranging from 1.65 to 5.87 g, with a coefficient of variation (CV) ranging from 16.01 to 16.22%. Conversely, MIY ranged from 0.55 to 15.27 g, with the highest CV ranging from 40.37 to 48.46%. This shows extensive variation in the association panel. CVs of all eight traits in the virtual environment (E4) were much higher than those in the natural environments (E1, E2, and E3), illustrating that YDTs are influenced by environmental variation, consistent with the ANOVA results that revealed significant differences in 3 investigated traits in terms of genotype, environment, and genotype-by-environment interactions. Relatively high broad-sense heritability (*h*^2^) was calculated for six traits (MIPN, BPN, PNP, SPP, TSW, and MIY), ranging from 56.52 to 82.30% (Table 1). Normal or approximately normal distribution was observed for all assessed traits (Figure 1). Significant positive correlations were observed between PNP and BPN, SPP and MIY, SPP and BY, BY and BPN, and BY and PNP. Negative correlations were observed between SPP and BPN, SPP and PNP, SPP and TSW, and MIY and BPN (Table 2).

**Table 1:** **Phenotypic variations for YDTs in the association panel**.

**Trait**	**Environment**	**Mean ±***SD*****	**Range**	**CV (%)**	**Skewness**	**Kurtosis**	**G**	**E**	**G × E**	***h*****^2^(%)**
MIPN	E1	81.50 ± 12.07	42.76 to 130.90	14.81	0.00	0.48	^**^	^**^	^**^	81.16
	E2	61.36 ± 15.54	12.67 to 98.79	25.32	−0.37	0.19				
	E3	88.01 ±16.57	33.60 to 138.20	18.83	−0.31	0.49				
	E4	0.35 ± 0.33	−0.72 to 1.42	92.97	0.37	0.90				
BPN	E1	297.00 ± 109.60	63.29 to 911.30	36.88	1.68	5.26	^**^	^**^	^**^	56.52
	E2	336.80 ± 127.70	83.29 to 881.80	37.92	0.84	1.19				
	E3	506.90 ± 146.60	143.40 to 983.60	28.92	0.41	0.08				
	E4	0.47 ± 0.44	−0.59 to 1.52	93.63	−0.08	−0.54				
PNP	E1	377.30 ± 113.90	138.10 to 1016.00	30.18	1.57	4.85	^**^	^**^	^**^	65.29
	E2	399.20 ± 131.40	130.00 to 965.60	32.91	0.86	1.29				
	E3	594.90 ± 146.60	222.40 to 1046.00	24.65	0.37	0.04				
	E4	0.45 ± 0.38	−0.46 to 1.34	83.89	−0.08	−0.54				
TSW	E1	3.15 ± 0.51	1.65 to 4.62	16.22	0.30	0.43	^**^	^**^	^**^	82.30
	E2	3.70 ± 0.59	2.00 to 5.87	16.01	0.46	1.81				
	E3	3.22 ± 0.52	2.04 to 5.66	16.06	0.03	−0.04				
	E4	−0.16 ± 0.19	−0.67 to 0.36	123.28	0.30	0.43				
SPP	E1	19.51 ± 4.15	3.48 to 29.82	21.25	−0.62	0.70	^**^	^**^	^**^	70.81
	E2	13.17 ± 4.60	3.87 to 30.24	34.93	0.73	0.78				
	E3	16.96 ± 6.48	3.31 to 33.74	38.21	0.45	−0.07				
	E4	0.17 ± 0.51	−1.18 to 1.24	293.22	−0.17	−0.25				
MIY	E1	–	–	–	–	–	^**^	^**^	^**^	73.14
	E2	3.47 ± 1.68	0.55 to 8.82	48.46	0.58	−0.04				
	E3	7.28 ± 2.94	0.90 to 15.27	40.37	0.41	−0.23				
	E4	0.65 ± 0.55	−1.23 to 1.73	83.39	−0.41	−0.14				
BY	E1	–	–	–	–	–	^**^	^**^	^**^	72.71
	E2	15.16 ± 5.07	3.85 to 37.46	33.46	0.52	0.45				
	E3	24.39 ± 9.14	2.47 to 51.41	37.47	0.07	−0.16				
	E4	0.44 ±0.42	−1.20 to 1.35	93.85	−0.63	1.32				

*MIPN, main inflorescence pod number; BPN, branch pod number; PNP, pod number per plant; SPP, seed number per pod; TSW, thousand seed weight; MIY, main inflorescence yield; BY, branch yield*.

** *The values are significant at P < 0.01 for the effect of genotype (G), environment (E) and genotype by environment interaction (G × E) on phenotypic variance estimated by two-way ANOVA. SD, standard deviation; CV, coefficient of variation; “–”, data not collected. h^2^, broad-sense heritability*.

**Table 2:** **Phenotypic correlations among YDTs in four environments**.

**Environment**	**Trait**	**MIPN**	**BPN**	**PNP**	**TSW**	**SPP**	**MIY**
E1	BPN	0.288^**^					
	PNP	0.3854^**^	0.9925^**^				
	TSW	0.0533	−0.1246^**^	−0.1070^*^			
	SPP	0.1544^**^	−0.1239^**^	−0.0989^*^	0.2433^**^		
E2	BPN	0.1882^**^					
	PNP	0.2970^**^	0.9937^**^				
	TSW	0.1653^**^	−0.2277^**^	−0.2082^**^			
	SPP	0.0550	−0.4598^**^	−0.4408^**^	−0.1014^*^		
	MIY	0.5464^**^	−0.0139	0.0469	0.2112^**^	0.4008^**^	
	BY	0.2164^**^	0.3821^**^	0.3960^**^	0.0536	0.4490^**^	0.3624^**^
E3	BPN	−0.0549					
	PNP	0.0581	0.9936^**^				
	TSW	−0.1372^*^	0.0583	0.0428			
	SPP	−0.0717	−0.4467^**^	−0.4553^**^	−0.2599^**^		
	MIY	0.2502^**^	−0.2488^**^	−0.2202^**^	0.0913	0.5704^**^	
	BY	−0.2047^**^	0.2534^**^	0.2299^**^	0.1358^**^	0.5442^**^	0.2517^**^
E4	BPN	0.0762					
	PNP	0.1954^**^	0.9915^**^				
	TSW	−0.0245	−0.0476	−0.0533			
	SPP	−0.1883^**^	−0.5601^**^	−0.5769^**^	−0.3594^**^		
	MIY	0.4108^**^	−0.1150	−0.0689	0.0128	0.3656^**^	
	BY	−0.2199^**^	0.2770^**^	0.2458^**^	−0.0499	0.4782^**^	0.0771

*MIPN, main inflorescence pod number; BPN, branch pod number; PNP, pod number per plant; SPP, seed number per pod; TSW, thousand seed weight; MIY, main inflorescence yield; BY, branch yield*.

* Significant at P < 0.05;

** *Significant at P < 0.01*.

### GWAS of YDTs

**Figure 2:**
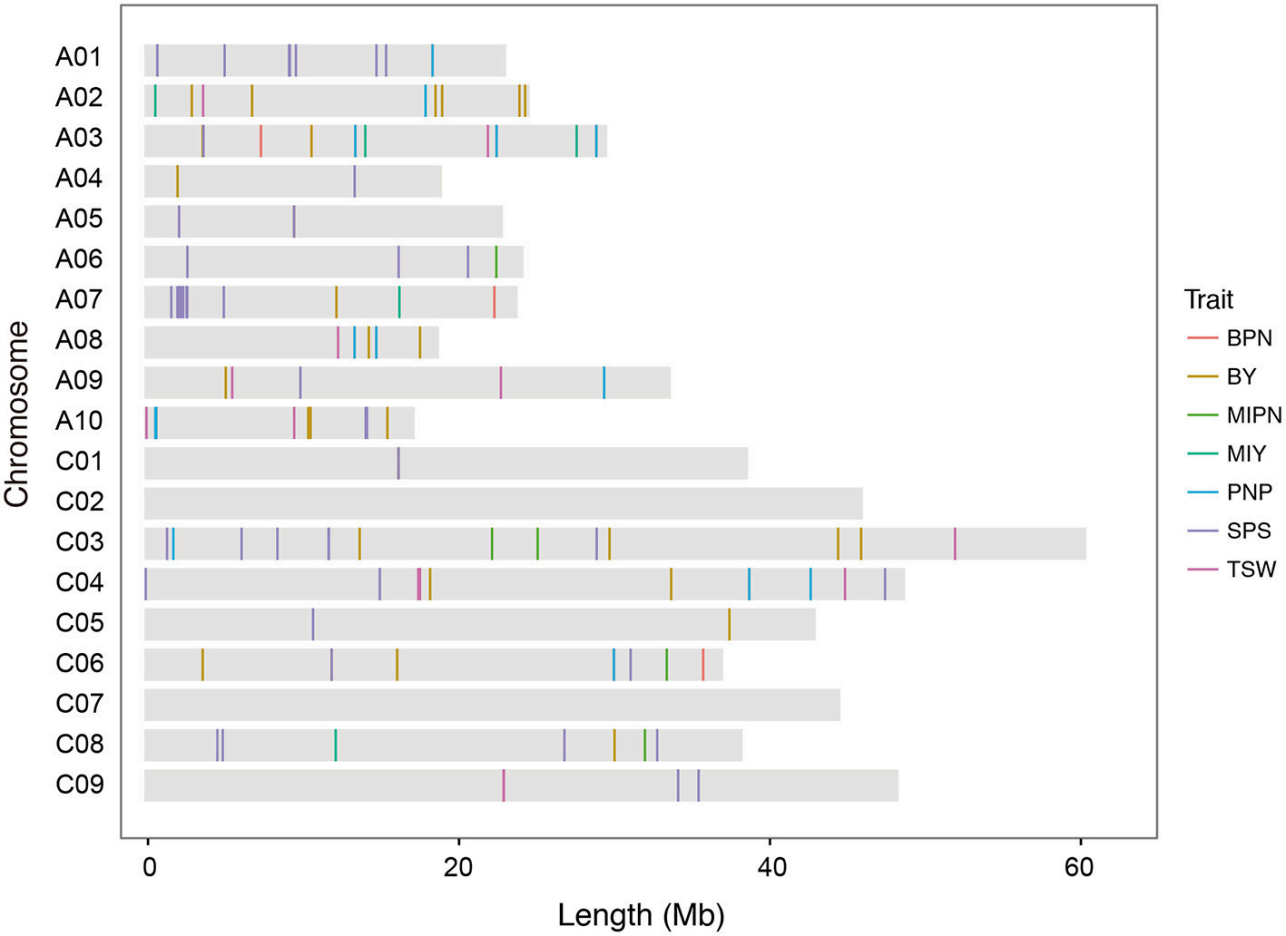
**Distribution of single nucleotide polymorphisms (SNPs) significantly associated with YDTs on the ***B. napus*** reference genome**. MIPN, main inflorescence pod number; BPN, branch pod number; PNP, pod number per plant; SPP, seed number per pod; TSW, thousand seed weight; MIY, main inflorescence yield; BY, branch yield.

In GWAS with the MLM model, 128 SNP–YDT associations were identified at a suggestive threshold (Figure 2, Supplementary Figures 1–4, and Supplementary Table 6), and 53 of these were detected at a significant threshold in the GLM model analysis. These SNPs were unevenly distributed across all chromosomes, except for chromosomes C02 and C07. Eighty significant SNPs were distributed across the A subgenome, while the remaining 48 SNPs were distributed across the C subgenome. Among the 128 SNPs, 51, 14, and 9 SNPs were significantly associated with three typical YCTs SPP, PNP, and TSW, respectively. The significant SNP–trait associations on the main inflorescence (both four SNPs for MIPN and MIY) were much less than those on the branches (16 SNPs for BPN and 30 SNPs for BY), implying the complexity of genetic architecture of traits on the branches. These significant associations individually explained 3.48–18.07% of the phenotypic variance. For YDTs in individual natural environments, between 1 and 25 SNPs were significantly associated with a single YDT. No SNP–trait associations were detected for E1-MIPN, E3-MIPN, or E3-MIY. In the virtual environment E4, 29 SNPs were significantly associated with YDTs, which individually explained 7.94–14.31% of the phenotypic variance (Supplementary Table 6). Estimating the total phenotypic variance for each YDT in the association panel using stepwise regression, we found that significant SNPs collectively accounted for 4.00–40.18% of the total phenotypic variance.

In the LD analyses, 15 HBs were associated with multiple traits (such as BPN and PNP), and six HBs contained multiple SNPs associated with an individual trait (Table 3). These HBs were used for the following candidate gene mining.

**Table 3:** **Summary of haplotype blocks consistently detected as associated with multiple traits, or as containing multiple single nucleotide polymorphisms associated with a single trait**.

**HB**	**Chr**.	**Associated trait**	***P*****-value (MLM)**	***R*****^2^ (MLM)**	**Range (bp)**	**Genes in the HB**	**Candidate gene**
1	A01	E1-SPP, E4-SPP	7.22E-05~1.86E-05	0.0503~0.1167	786,674–873,224	BnaA01g01460D-BnaA01g01640D	*Bna.ROP3* (*BnaA01g01610D, AT2G17800*)
2	A01	E3-SPP, E4-SPP	5.47E-05~8.30E-05	0.0958~0.1151	5,042,605–5,187,842	BnaA01g10130D-BnaA01g10390D	*Bna.QRT3* (*BnaA01g10390D, AT4G20050*)
3	A01	E1-BPN, E1-PNP	1.89E-05~2.75E-05	0.0492~0.0509	18,466,247–18,579,414	BnaA01g26410D-BnaA01g26530D	
4	A02	E1-BPN, E1-PNP	4.70E-12~2.00E-12	0.1192~0.1233	17,990,431–18,286,433	BnaA02g24670D-BnaA02g24930D	
5	A03	E3-BPN, E3-PNP	9.07E-05~9.17E-05	0.0853~0.0854	13,494,779–13,589,341	BnaA03g27360D-BnaA03g27650D	*Bna.PEL7* (*BnaA03g27540D, AT3G01270*)
6	A03	E1-BPN, E1-PNP, E4-TSW	6.53E-06~4.32E-05	0.0555~0.0794	22,079,719–22,660,395	BnaA03g43820D-BnaA03g44650D	*Bna.TAP35* (*BnaA03g44550D, AT4G20420*)
7	A03	E2-BPN, E2-PNP	7.94E-05~5.72E-05	0.0451~0.0467	29,073,873–29,098,026	BnaA03g54460D-BnaA03g54480D	
8	A05	E3-BY, E3-SPP	2.83E-06~2.61E-05	0.1027~0.1299	9,531,586–9,731,586	BnaA05g29380D-BnaA05g32400D	*Bna.MYB83* (*BnaA05g29680D, AT3G08500*)
9	A07	E3-SPP	3.98E-06~6.38E-05	0.0944~0.1287	2,125,090-2,910,402	BnaA07g02520D-BnaA07g03250D	
10	A08	E2-BPN, E2-PNP	3.96E-05~6.25E-05	0.0463~0.0482	13,520,923–13,598,303	BnaA08g16720D-BnaA08g16910D	*Bna.BBX20* (*BnaA08g16780D, AT4G39070*)
11	A08	E1-BPN, E1-PNP	3.60E-05~5.81E-05	0.0460~0.0480	14,822,078–15,022,078	BnaA08g19200D-BnaA08g19550D	*Bna.BBX15* (*BnaA08g19420D, AT1G25440*)
12	A09	E2-BPN, E2-PNP	5.13E-05~5.66E-05	0.0408~0.0413	29,464,731–29,598,718	BnaA09g42360D-BnaA09g42490D	*Bna.BRK1* (*BnaA09g42410D, AT2G22640*)
13	A10	E2-BPN, E2-PNP	3.18E-06~1.58E-05	0.0524~0.0866	486,257–784,765	BnaA10g00920D-BnaA10g01540D	*Bna.LEA3* (*BnaA10g01410D, AT1G02820*)
14	A10	E1-SPP, E4-SPP	8.23E-05~4.78E-05	0.0438~0.1216	14,240,793–14,329,378	BnaA10g20290D-BnaA10g20540D	*Bna.LRP1* (*BnaA10g20370D, AT5G12330*)
15	C01	E3-BY, E3-SPP	5.30E-08~1.58E-06	0.1293~0.1703	15,864,962–16,351,149	BnaC01g22420D-BnaC01g22820D	*Bna.PRP17* (*BnaC01g22500D, AT4G15160*)
16	C04	E1-BPN, E1-PNP	1.13E-07~3.30E-07	0.0688~0.0736	42,450,406–42,860,566	BnaC04g41700D-BnaC04g42260D	*Bna.bHLH91* (*BnaC04g42030D, AT2G31210*)
17	C06	E3-BY, E3-SPP	2.89E-05~4.09E-05	0.0863~0.0938	12,052,153–12,534,523	BnaC06g10070D-BnaC06g10370D	*Bna.SPL5* (*BnaC06g10070D, AT3G15270*)
18	C06	E1-BPN, E1-PNP	2.99E-06~6.45E-06	0.0556~0.0591	30,202,408–30,337,696	BnaC06g29120D-BnaC06g29450D	
19	C08	E4-SPP	5.05E-05~9.31E-05	0.1137~0.1209	4,546,027–5,462,228	BnaC08g04150D-BnaC08g04770D	*Bna.SUS2* (*BnaC08g04400D, AT1G80070*)
20	C09	E3-TSW	1.10E-05~3.45E-05	0.0785~0.0869	23,116,889–23,117,415		
21	C09	E3-BY, E3-SPP	6.46E-06~4.47E-05	0.0855~0.1080	34,334,321–34,506,204	BnaC09g31320D-BnaC09g31560D	

*Chr, chromosome; R^2^ is the percentage of phenotypic variance explained by single nucleotide polymorphism*.

### Transcriptome analysis for identifying DEGs

In the transcriptome analyses, four extremely high- and low-SY accessions at CQ and YN were separatively chosen based on their SY and kinship coefficients (Supplementary Table 4). Calculation of kinship coefficients between plant materials used for transcriptome sequencing revealed that only B400 showed high kinship (higher than 0.5) with B206 and B376 among the 10 uniquely selected sequencing samples. Most of kinship coefficients between the rest *B. napus* accessions ranged from 0 to 0.2, suggesting that these materials have no kinship or a relatively weak kinship, which offers guarantee for the accuracy of DEG identification.

After filtering out low quality sequencing reads and trimming of the first 5 bp error-enriched nucleotides at the 5′ end, approximate 256 gigabases (Gb) of 120-bp paired-end reads were remained, with an average of 16.72 million reads per sample, as per our previous description. Mapping results showed that about 84% of the input reads uniquely mapped to the *B. napus* reference genome. Then, these mapped reads were used for estimation of FPKM-based transcript abundance values for all RNA-seq samples.

**Figure 3:**
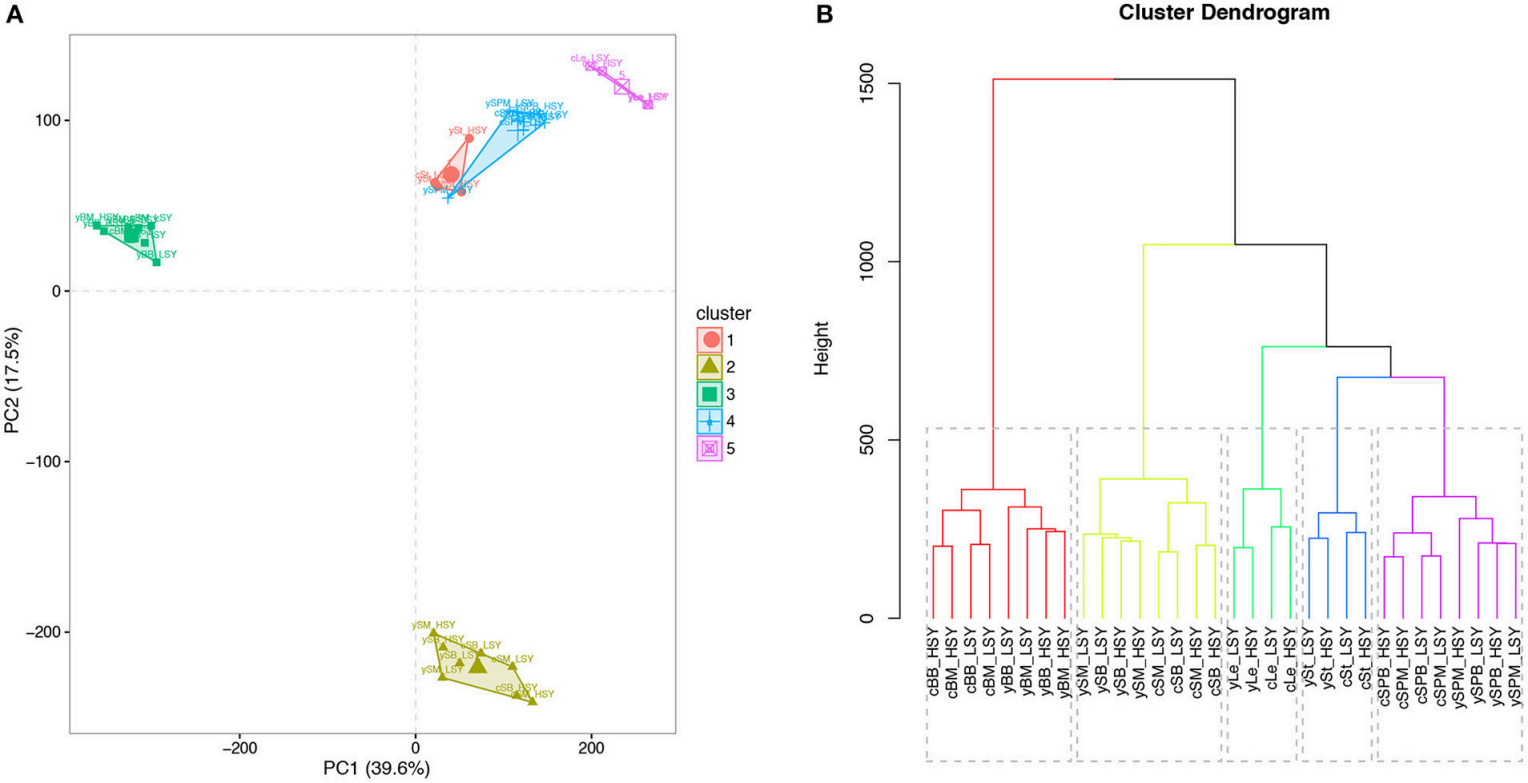
**Principal component analysis (PCA) plot and cluster analysis of all RNA-seq samples. (A)** PCA plot of all RNA-seq samples. **(B)** Cluster dendrogram of all RNA-seq samples. First letters (c and y) represent cultivation regions Chongqing (CQ) and Yunnan (YN), respectively. St, stems; Le, leaves; BM, buds on the main inflorescence; BB, buds on the primary branch; SPM, silique pericarps on the main inflorescence; SPB, silique pericarps on the primary branch; SM, seeds harvested 20 days after flowering on the main inflorescence; SB, seeds harvested 20 days after flowering on the primary branch; LSY, low seed yield accessions; HSY, high seed yield accessions. The log2-nomalized (FPMK+1) values of all the genes were used for PCA and cluster analyses.

The Pearson correlation coefficients (PCCs) between all RNA-seq samples were calculated from log2-transformed (FPKM+1) values (Figure 3). Comparing results revealed that the PCCs between samples harvested from the same tissues were higher than those between samples collected from different tissues. Results from PCA and cluster analysis were consistent with the abovementioned correlation analysis (Supplementary Figure 5), suggesting that our RNA-seq results met the requirement of following DEG identification.

**Figure 4:**
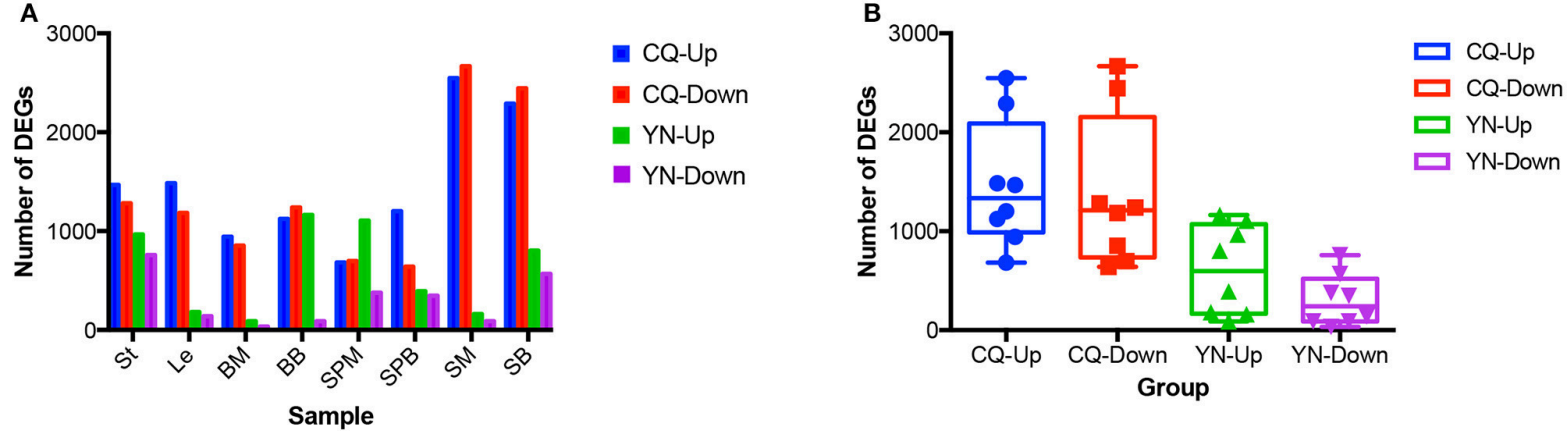
**Comparison of differentially expressed genes (DEGs) between high-yield and low-yield accessions harvested from two different cultivation regions. (A)** Number of up-regulated and down-regulated DEGs in eight different tissues from high-yield and low-yield accessions grown at Yunnan (YN) and Chongqing (CQ). **(B)** Average number of up-regulated and down-regulated DEGs in all tissues between high-yield and low-yield accessions grown at YN and CQ. “Up” and “down” indicate up-regulated and down-regulated genes, respectively.

Pair-wise comparisons between the same tissues from high-SY and low-SY accessions were conducted to call the DEGs using the aforementioned standards. Between 250 and 5,215 genes were differentially expressed in individual tissues (Figure 4). Of these DEGs, the average number in most tissues at CQ (2,844) was much higher than those at YN (908), except for SPM; this indicates that the transcriptomic variation between high-SY and low-SY accessions was greater at CQ. The number of DEGs was different among the eight tissues at the two locations: a relatively higher number of DEGs were found in SM, SB, St, and Le at CQ, but the number of DEGs in St, SPM, SB, and BB at YN was relatively higher than in other tissues at YN.

### Functional enrichment analysis

Based on the GO enrichment analysis, between 41 and 995 significantly overrepresented GO terms were identified in DEGs in individual tissues between the high-SY and low-SY accessions; of these, the average number of overrepresented GO terms in up-regulated genes was higher than those in down-regulated genes at YN, while the reverse trend was observed for DEGs at CQ. There were fewer overrepresented GO terms in DEGs from buds than those in other tissues. DEGs in the stem at both locations were enriched into 1,203 GO terms; the highest among the eight tissues investigated.

The most prevalent GO terms in up-regulated and down-regulated genes in BM and BB of high-SY accessions at CQ were pollen wall assembly (GO:0010208) and cell wall modification (GO:0042545), respectively. Up-regulated genes in BM and BB at YN were enriched in nutrient reservoir activity (GO:0045735), while no significantly enriched GO term was identified for down-regulated genes in BB. In leaves from plants at both locations, up-regulated genes were significantly enriched for GO terms including photosynthesis (GO:0015979) and photosynthesis and light harvesting (GO:0009765), but defense response (GO:0006952) and galactolipid biosynthetic processes (GO:0019375) were the most significantly enriched GO terms in the down-regulated genes at CQ and YN, respectively. Overrepresented GO terms among DEGs in the stem were different between the two locations; glycosinolate biosynthetic process (GO:0019758) was the most significantly enriched GO term both for up-regulated genes at YN and down-regulated genes at CQ. GO terms significantly enriched in the silique pericarps were different between the main inflorescence and the primary branch. Secondary metabolic processes such as anthocyanin biosynthesis (GO:0009718) and phenylpropanoid biosynthesis (GO:0009699) were the most significantly enriched GO terms for up-regulated genes in SPM, whereas stress response-related GO terms, such as response to organic substances (GO:0010033) and response to ultraviolet light (UV, GO:0009411) were the most significantly enriched terms for up-regulated genes in SPB. For up-regulated genes, pollen exine formation (GO:0010584) and lipid localization (GO:0010876) were highly enriched in the SM and SB at YN, while RNA methylation (GO:0001510) and seed dormancy (GO:0010162) were mostly significant enriched within the SM and SB at CQ, respectively. More significantly enriched GO terms are shown in Supplementary Table 6.

### Discovery candidate genes for YDTs by integrating GWAS and RNA-seq datasets

Among the 21 HBs with particular interest, a total of 1,107 genes were retrieved, according to the *B. napus* reference genome annotation (Supplementary Table 7). There were 635 DEGs in at least one tissue from plants grown at YN or CQ. Subsequently, we combined GWAS and RNA-seq results to mine candidate genes in each HB (Supplementary Table 8). Finally, 14 candidate genes were selected from 21 HBs; no candidate genes were found in the other 7 HBs. As an example, we found 30 genes within HB5 on chromosome A03. Since HB5 significantly associated with E3-BPN and E3-PNP, only eight DEGs in seeds or silique pericarps on branches at YN were chosen for further functional annotation. Then, the up-regulated pectate lyase gene *Bna.PEL7* (*BnaA03g27540D*) was identified as a key candidate within HB5, because of its putative function in the growth of pollen tubes in female floral tissues. Candidate genes within other HBs were screened out using this combination method (Table 3).

Functional annotation of these 14 candidates revealed that five genes were involved in transcription factor activity, and six in developmental processes. For example, *Bna.MYB83* (*BnaA05g29680D*), located within HB8 of SNP rs10508, associated with BY and SPP in E3 (Table 3) and encoded an ortholog of a putative R2R3-type MYB transcription factor MYB83 (AT3G08500), a master transcriptional regulator responsible for secondary wall biosynthesis and biomass production in *Arabidopsis* (Zhong et al., 2015). *Bna.SPL5* (*BnaC06g10070D*) was a candidate gene found in HB17 of SNP rs36347, which associated with BY and SPP in E3 and encoded SQUAMOSA PROMOTER BINDING PROTEIN-LIKE 5 (AtSPL5, AT3G15270) in *Arabidopsis*, which is involved in the regulation of flowering and vegetative phase change (Xu et al., 2016). All candidate genes within HBs are listed in Table 3, including orthologs of *QUARTET3* (*Bna.QTR3, BnaA01g10390D*), *BRICK1* (*Bna.BRK1, BnaA09g42410D*) and *LATE EMBRYOGENESIS ABUNDANT3* (*Bna.LEA3, BnaA10g01410D*) in *Arabidopsis*.

### Validation of DEGs

To validate the accuracy of identified DEGs, we determined the expression patterns of the 14 candidate genes by qRT-PCR. To avoid non-specific amplification, amplified PCR products were recovered and confirmed by sequencing. All amplified qRT-PCR products investigated revealed only a single target product with the desired length for each candidate gene. The PCC was estimated to evaluate the consistency between RNA-seq and qRT-PCR results (Supplementary Figure 6, 7). As expected, PCC was high (*r*^2^ = 0.8846), suggesting the reliability and accuracy of RNA-seq data.

## Discussion

Seed (or grain) yield is one of the most economically important and complex crop traits (Richards, 2000; Lobell et al., 2009; Cai et al., 2014; Shi et al., 2015). SY manifests via a complex relationship between the YCTs PNP, SPP, and TSW in oilseed crops, or thousand grain weight (TGW), grains per spike (GPS), and grain weight per spike (GWS) in cereal crops. The three YCTs vary among accessions of *B. napus*, and different combinations of YCTs can lead to similar yield production (Cai et al., 2016); thus, it is difficult to realize high-yield breeding through the selection of high-yield genotypes.

In the present study, we investigated the genetic basis for variation in seven YDTs in natural populations of 520 *B. napus* accessions. Significant phenotypic variation and correlations between YDTs were observed. BY significantly positively correlated with other YDTs under natural environments, except with TSW at CQ, as reported previously (Cai et al., 2014; Wang X. et al., 2016). We also calculated the correlation coefficients between YDT trait values in the virtual environment (representing the environmental response for each trait) and found no significant correlations between TSW and MIY, or between TSW and BY (Table 3). Hence, we speculate that TSW is more stable than other YDTs because the correlation between SY (the sum of BY and MIY) and TSW was less sensitive to environmental change. TSW might be a prime breeding target for the development of high-yielding *B. napus* cultivars with a strong ability to adapt, especially those that will be cultivated in different cultivation environments.

Numerous QTLs for YDTs in *B. napus* have been identified by QTL mapping and GWAS (Quijada et al., 2006; Chen et al., 2011; Zhang et al., 2011; Zhou et al., 2014; Fu et al., 2015; Shi et al., 2015; Zhao et al., 2016). Using the *B. napus* genome sequence as a reference, we compared the physical location of significant SNPs identified in this study with QTLs from previous studies. Among our 128 significant SNP–trait associations, 35 SNPs for YDTs identified in this study were located in, or overlapped with, confidence intervals of QTLs reported previously, whereas the 93 remaining SNPs are novel loci (Supplementary Table 6). Based on previous reports, there is a major QTL simultaneously associated with SL and TSW on chromosome A9 in *B. napus* (Yang et al., 2012; Fu et al., 2015; Liu et al., 2015). Fan et al. (2010) identified two stable major QTLs, *TSWA7a* and *TSWA7b*, which collectively explained 27.6–37.9% of the trait variation in another DH population, but didn't detected the major QTL on chromosome A9. In this study, we failed to confirm the effects from the QTLs *cqSWA9, TSWA7a*, and *TSWA7b*, though nine significant SNPs for TSW were identified. The reason why their effects may not have been confirmed is because they were highly influenced by the genetic background. So far, there were three research groups reported the major QTL *cqSWA9* at the same QTL region on chromosome A9. We found that the parental lines (zy72360, SWU07, and S1) used for mapping population construction may share the similar genetic background, since all the three lines were semi-winter type, long SL and large SW. More importantly, the major QTL *cqSWA9* was found to be associated with the SL and SW simultaneously in the three studies. The failure to confirm *cqSWA9* QTL in this study and Fan et al.'s findings was probably due to the difference of genetic background, as the SJ-DH population in Fan et al.'s study was produced from a cross between SW Hickory (a spring-type *B. napus* variety) and JA177 (a winter-type *B. napus* pure line), and a natural population was adopted for GWAS in this study.

We noticed that there were 35 common QTLs associated with different traits to those previously identified, implying that some are likely to be pleiotropic QTLs that simultaneously regulate at least two different YDTs. For example, the significant association between SNP rs45027 and MIPN was observed in the current study, and the HB harboring rs45027 overlapped with QTL confidence intervals for seed number (*qSN.C06-2*), seed weight (*cqSW-C6-3*), and SY (*cqSY-C6-3*) (Shi et al., 2015; Zhao et al., 2016). Pleiotropic QTLs identified across different studies will be important targets of yield-related gene cloning for elucidating the molecular mechanism of YDTs in *B. napus*. These results indicate that YDTs are complex traits controlled by an array of yield-related genes that are unevenly distributed on the subgenomes of *B. napus*.

In addition to common QTLs, 93 novel loci were detected in this study (Supplementary Table 6). To maximize the detection power, researchers generally prefer the permissive model, GLM, which potentially introduces more false positives than the stringent mixed model, MLM. To reduce the risk of type 1 error, the 93 novel association loci were all identified based on the MLM, implying that these association signals are fairly reliable, and deserving further validation in other population and environments.

Chromosome distribution analysis showed that 128 significant SNPs identified in this study were unevenly distributed on the A and C subgenomes of *B. napus*; 80 significant SNPs were distributed on the A subgenome, and the remaining 48 SNPs were distributed on the C subgenome (Supplementary Table 6). In QTL or association mapping studies, other researchers have also identified more QTL or SNP–trait associations in the A-subgenome than C-subgenome of *B. napus* (Cai et al., 2014, 2016; Shi et al., 2015; Wang X. et al., 2016). For example, Shi et al. identified eight and 16 QTLs for PNP and SPP, respectively, but only two and three QTLs on the C subgenome, respectively (Shi et al., 2015). An uneven distribution of significant signals for other traits was also observed. GWAS identified 26 SNPs significantly associated with *Sclerotinia sclerotiorum* resistance in *B. napus*, which corresponded to three loci on the C subgenome (Wu et al., 2016). Using GWAS with MLM, Liu et al. identified 11 *B. napus* SNPs significantly associated with seed oil content, corresponding to five loci located on chromosomes A3, A5, A9, A10, and C7, exhibiting an A-subgenome preference (Liu S. et al., 2016). The fact that different traits seem to be unevenly distributed in different ways at the subgenome level suggests that expansion of our existing genetic resources might be warranted to target traits for breeding. Indeed, the resynthesis of *B. napus* has already led to the successful transfer of resistance to *S. sclerotiorum* from wild-type *B. oleracea* (Ding et al., 2013). Further exploration of the A subgenome (or the A subgenome donor *B. rapa*) will be crucial for further improving YDTs in *B. napus*.

As a powerful means to identify genomic regions (loci) associated with complex traits, GWAS has been successfully employed to understand the genetic basis of several agronomically important traits in crops (Huang and Han, 2014), and has become a standard tool for candidate gene discovery. Though numerous key loci associated with complex traits have been identified by GWAS in crops, it remains arduous to fine-map and clone the target genes responsible for the phenotypic variation of those traits, and difficult to achieve a comprehensive understanding of the genetic mechanisms. To overcome these challenges, several “omics” profiling approaches (transcriptome, proteome, metabolome, epigenome, and microbiome) were recently integrated with GWAS to functionally characterize the associations (van der Sijde et al., 2014). Although hurdles remain, these combined methods have shown promise in investigating the molecular mechanisms underlying complex traits.

Transcriptome sequencing (RNA-seq) has been widely used to estimate gene expression changes, and enables the detection of novel transcripts (both coding genes and several kinds of non-coding RNA), alternative spliced transcripts, gene fusions, and post-transcriptional modifications. To increase the efficiency and accuracy of candidate gene discovery in GWAS, resolution has been increased by combining GWAS (or QTL mapping) with and RNA-seq (Hua et al., 2016; Li L. et al., 2016; Lu et al., 2016; Wei et al., 2016; Wu et al., 2016). For example, Hua et al. revealed a *NODULIN 26-LIKE INTRINSIC PROTEIN* (*NIP*) gene underlying the major boron uptake efficiency QTL *qBEC-A3a* in *B. napus* by integrating QTL fine mapping with digital gene expression profiling (Hua et al., 2016). Integrated RNA-seq data from *B. rapa* and GWAS by Li et al. indicated that the Toll Interleukin1 Receptor–Nucleotide Binding Site (TIR-NBS) gene family might be important in clubroot (*Plasmodiophora brassicae* Woronin) resistance (Li L. et al., 2016). In this work, we retrieved 1,107 genes from 21 HBs based on GWAS and LD analysis. Of these genes, 635 were differentially expressed at least in one tissue from plants grown at YN or CQ. Subsequently, we combined GWAS and RNA-seq results to mine candidate genes in each HB, and identified 14 candidate genes based on their expression patterns and biological function. It is obviously that the combination of GWAS and RNA-seq (or other “omics” technologies) provides higher resolution in illuminating the genetic basis of complex traits, and is likely to become a widely used standard approach.

To rapidly identify candidate genes controlling YDTs, 21 HBs associated with multiple traits, or containing multiple SNPs associated with the same trait, were chosen for candidate gene identification using the combination of strategies described above. Among these 21 HBs, 14 DEG candidates were obtained, including five transcription factors (TFs) and six genes involved in developmental processes (Table 3). MYB and bHLH (basic helix–loop–helix) TFs may be crucial regulators of YDTs. Within HB8, *BnaA05g29680D* was identified as the most plausible candidate gene, which encodes an ortholog of the MYB TF *AtMYB83* in *Arabidopsis*. Overexpression of *MYB83* activates several genes involved in cellulose, xylan, and lignin biosynthesis, and concomitantly induces ectopic secondary wall deposition (McCarthy et al., 2009), providing a valuable approach to increase crop biomass production. In this study, up-regulation of *Bna.MYB83* expression was detected in SPM, SPB, and SB, implying that yield increase might be caused by higher biomass accumulation at SP, an important photosynthesis organ for seed filling during the reproductive stage. Within HB16 on chromosome C04, *BnaC04g42030D* encodes an ortholog of AtbHLH91 in *Arabidopsis*. This gene is important for *Arabidopsis* another development, possibly by forming a feed-forward loop with DYSFUNCTIONAL TAPETUM1 (DYT1) (Zhu et al., 2015). Expression of *Bna.bHLH91* was significantly up-regulated in seeds from CQ, suggesting that this gene may regulate yield increase.

Several genes involved in developmental processes were also considered as important candidates for YDT variation. In HB1 on chromosome A01, we identified *BnaA01g01610D*, which encodes an ortholog of *AtROP3*, an important gene underlying embryo development and auxin-dependent plant growth (Huang et al., 2014). Significant up-regulation of *Bna.ROP3* expression in the SM at YN and CQ suggests a crucial role for regulating seed and silique development. Within HB2, *BnaA01g10390D* was significantly up-regulated in silique pericarps of high-yielding *B. napus* accessions grown at YN; this gene encodes an ortholog of QUARTET3 in *Arabidopsis*. *AtQRT3* mutations cause failure of microspore separation during pollen development because of a defect in degradation of the pollen mother cell wall during the late stages of pollen development (Rhee et al., 2003). Hence, we propose that *Bna.QRT3* is also a key regulator of high yield in *B. napus*. Key candidate genes within other HBs are summarized in Table 3. These candidate genes are valuable resources for further experimental verification, and may be important in high-yield *B. napus* breeding.

## Author contributions

KL, JLi, and YL designed the work; KL, LP, CZ, JLu, BY, ZX, QZ, MF, and XY performed the experiments; KL, YL, XX, KZ, LL, and CQ contributed to data analysis and interpretation; KL, LP, and JLi drafted the paper. All authors reviewed the manuscript.

## Funding

This work was supported by grants from the National Natural Science Foundation of China (U1302266, 31571701 and 31360346), the National Key Research and Development Plan (2016YFD0101007), the 973 Project (2015CB150201), the National High Technology Research and Development Programs of China (2013AA102602), the National Key Technology Support Program (2013BAD01B03-12) and the 111 Project (B12006).

### Conflict of interest statement

The authors declare that the research was conducted in the absence of any commercial or financial relationships that could be construed as a potential conflict of interest.
