# Supplementary material for: Genome-Wide Association and Transcriptome Analyses Reveal Candidate Genes Underlying Yield-determining Traits in Brassica napus
Source: Front Plant Sci. 2017 Feb 15;8:206. doi: 10.3389/fpls.2017.00206 (PMC5309214; doi:10.3389/fpls.2017.00206)
Supplement: Supplementary file 1 [file Table1.PDF]

Supplementary Material

Genome-Wide Association and Transcriptome Analyses Reveal Candidate Genes Underlying Yield-determining Traits in *Brassica napus*

Kun Lu[1†*], Liu Peng[1,2†], Chao Zhang[1,3], Junhua Lu[1], Bo Yang[1], Zhongchun Xiao[1], Ying Liang[1], Xingfu Xu[1], Cunmin Qu[1], Kai Zhang[1], Liezhao Liu[1], Qinlong Zhu[4], Minglian Fu[5], Xiaoyan Yuan[5], Jiana Li[1*]

*** Correspondence:**

Kun Lu: drlukun@swu.edu.cn
Jiana Li: ljn1950@swu.edu.cn

Supplementary Table S1. Meteorological data from two cultivation regions, CQ and YN

Environment	Date	Max air temp (℃)	Average air temp (℃)	Min air temp (℃)	Average diurnal temp difference (℃)	Daily precipitation (mm)	Relative humidity (%)	Illumination time (h)	Monthly averaged direct normal radiation (kWh m^{-2} d^{-1})
CQ	Sep, 2013	26.36	23.30	20.10	6.26	3.27	77.43	13.05	1.99
	Oct, 2013	23.16	19.68	16.29	6.87	2.02	76.16	12.18	NA
	Nov, 2013	16.23	14.10	12.17	4.06	1.22	84.93	11.45	1.55
	Dec, 2013	11.42	8.52	5.81	5.61	0.32	81.70	11.12	1.58
	Jan, 2014	11.39	8.13	5.06	6.33	0.13	78.35	11.35	1.61
	Feb, 2014	11.18	8.68	6.32	4.86	0.43	74.39	12.03	1.56
	Mar, 2014	18.16	14.39	10.94	7.22	4.38	72.84	12.83	2.06
	Apr, 2014	22.87	19.57	16.27	6.60	2.71	77.53	13.77	2.25
	May, 2014	24.61	21.00	17.74	6.87	2.86	75.77	14.55	2.32
YN	Sep, 2013	25.63	21.70	17.87	7.76	3.98	76.17	12.97	3.00
	Oct, 2013	23.23	19.09	15.03	8.20	3.01	74.19	12.30	3.55
	Nov, 2013	23.73	16.70	9.87	13.86	0.17	63.90	11.73	4.92
	Dec,2013	18.55	12.00	5.32	13.23	0.70	66.16	11.48	5.76
	Jan, 2014	21.29	13.06	4.87	16.42	0.00	51.77	11.65	7.17
	Feb, 2014	22.18	15.00	7.93	14.25	0.43	44.82	12.18	6.91
	Mar, 2014	25.29	17.97	10.55	14.74	0.20	37.03	12.80	5.93
	Apr, 2014	28.27	21.07	13.77	14.50	0.33	31.13	13.53	5.26
	May, 2014	28.68	23.13	17.48	11.20	0.90	46.39	14.15	4.02

The monthly averaged direct normal radiation was calculated from Nasa data spanning the 22 years from 1983 to 2005.
NA means absence in that month
