# Supplementary material for: Genome-Wide Association and Transcriptome Analyses Reveal Candidate Genes Underlying Yield-determining Traits in Brassica napus
Source: Front Plant Sci. 2017 Feb 15;8:206. doi: 10.3389/fpls.2017.00206 (PMC5309214; doi:10.3389/fpls.2017.00206)
Supplement: Supplementary file 2 [file Table2.PDF]

Supplementary Material

Genome-Wide Association and Transcriptome Analyses Reveal Candidate Genes Underlying Yield-determining Traits in *Brassica napus*

Kun Lu[1†*], Liu Peng[1,2†], Chao Zhang[1,3], Junhua Lu[1], Bo Yang[1], Zhongchun Xiao[1], Ying Liang[1], Xingfu Xu[1], Cunmin Qu[1], Kai Zhang[1], Liezhao Liu[1], Qinlong Zhu[4], Minglian Fu[5], Xiaoyan Yuan[5], Jiana Li[1*]

*** Correspondence:**

Kun Lu: drlukun@swu.edu.cn
Jiana Li: ljn1950@swu.edu.cn

Supplementary Table S2. Traits and measurement methods in this study

Trait	Measurement methods	Environment	Unit
Main inflorescence pod number per plant (MIPN)	The number of effective pods on the main inflorescence of each harvested individual, which was measured as the average of five plants	E1, E2, E3 and E4	
Branch pod number (BPN)	The number of effective pods on the secondary and tertiary branches of each harvested individual, which was measured as the average of five plants	E1, E2, E3 and E4	
Pod number per plant (PNP)	The number of effective pods on the main inflorescence and branches of each harvested individual, which was measured as the average of five plants	E1, E2, E3 and E4	
Seed number per pod (SPP)	The number of seeds in each harvested pod, which was counted for the average of ten random pods.	E1, E2, E3 and E4	
Thousand seed weight (TSW)	The average dry weight in grams of 1000 well-filled seeds mixed from five sampled plants	E1, E2, E3 and E4	g
Main inflorescence yield (MIY)	The dry weight of seeds on the main inflorescence of each harvested individual, which was measured as the average of five plants	E2, E3 and E4	g/plant
Branch yield (BY)	The average dry weight of seeds on the branches of each harvested individual, which was measured as the average of five plants	E2, E3 and E4	g/plant
