# Supplementary material for: Genome-Wide Association and Transcriptome Analyses Reveal Candidate Genes Underlying Yield-determining Traits in Brassica napus
Source: Front Plant Sci. 2017 Feb 15;8:206. doi: 10.3389/fpls.2017.00206 (PMC5309214; doi:10.3389/fpls.2017.00206)
Supplement: Supplementary file 3 [file Table3.PDF]

Supplementary Material

Genome-Wide Association and Transcriptome Analyses Reveal Candidate Genes Underlying Yield-determining Traits in *Brassica napus*

Kun Lu[1†*], Liu Peng[1,2†], Chao Zhang[1,3], Junhua Lu[1], Bo Yang[1], Zhongchun Xiao[1], Ying Liang[1], Xingfu Xu[1], Cunmin Qu[1], Kai Zhang[1], Liezhao Liu[1], Qinlong Zhu[4], Minglian Fu[5], Xiaoyan Yuan[5], Jiana Li[1*]

*** Correspondence:**

Kun Lu: drlukun@swu.edu.cn
Jiana Li: ljn1950@swu.edu.cn

Supplementary Table S3. Plant materials used for transcriptome sequencing

Location	Group	Accession number	Accession name	Provider
YN	high yield	B400	Zhongshuang4	ORI, CAAS
		B163	09−P64−1	HAZU
		B376	Zhongshuang12	ORI, CAAS
		B18	SWU47	SWU
	low yield	B25	SWU82	SWU
		B56	R431	SWU
		B124	2012−9323	SWU
		B206	Ningyou12	JAAS
CQ	high yield	B376	Zhongshuang12	ORI, CAAS
		B25	SWU82	SWU
		B400	Zhongshuang4	ORI, CAAS
		B163	09−P64−1	HAZU
	low yield	B58	10−1047	HNZU
		B141	Huayou2	HAZU
		B124	2012−9323	SWU
		B206	Ningyou12	JAAS

CQ, Chongqing; YN, Yunnan; HAZU, Huazhong Agricultural University; HNZU, Hunan Agricultural University; ORI, CAAS, Oilcrop Research Institute, Chinese Academy of Agricultural Sciences; SWU, Southwest University; JAAS, Jiangsu Academy of Agricultural Sciences
