# Supplementary material for: Genome-Wide Association and Transcriptome Analyses Reveal Candidate Genes Underlying Yield-determining Traits in Brassica napus
Source: Front Plant Sci. 2017 Feb 15;8:206. doi: 10.3389/fpls.2017.00206 (PMC5309214; doi:10.3389/fpls.2017.00206)
Supplement: Supplementary file 4 [file Table4.PDF]

Supplementary Material

Genome-Wide Association and Transcriptome Analyses Reveal Candidate Genes Underlying Yield-determining Traits in *Brassica napus*

Kun Lu[1†*], Liu Peng[1,2†], Chao Zhang[1,3], Junhua Lu[1], Bo Yang[1], Zhongchun Xiao[1], Ying Liang[1], Xingfu Xu[1], Cunmin Qu[1], Kai Zhang[1], Liezhao Liu[1], Qinlong Zhu[4], Minglian Fu[5], Xiaoyan Yuan[5], Jiana Li[1*]

*** Correspondence:**

Kun Lu: drlukun@swu.edu.cn
Jiana Li: ljn1950@swu.edu.cn

Supplementary Table S4. Kinship coefficients between plant materials used for transcriptome sequencing

Accession number	B18	B25	B56	B58	B124	B141	B163	B206	B376	B400
B18	2.000	0.444	0.094	0.000	0.000	0.296	0.118	0.063	0.126	0.044
B25	0.444	2.000	0.242	0.060	0.000	0.082	0.000	0.006	0.000	0.062
B56	0.094	0.242	2.000	0.170	0.000	0.218	0.260	0.188	0.000	0.000
B58	0.000	0.060	0.170	2.000	0.000	0.095	0.000	0.192	0.027	0.000
B124	0.000	0.000	0.000	0.000	2.000	0.000	0.131	0.000	0.000	0.095
B141	0.296	0.082	0.218	0.095	0.000	2.000	0.000	0.183	0.000	0.000
B163	0.118	0.000	0.260	0.000	0.131	0.000	2.000	0.172	0.000	0.000
B206	0.063	0.006	0.188	0.192	0.000	0.183	0.172	2.000	0.000	1.000
B376	0.126	0.000	0.000	0.027	0.000	0.000	0.000	0.000	2.000	0.576
B400	0.044	0.062	0.000	0.000	0.095	0.000	0.000	1.000	0.576	2.000
