# Supplementary material for: Genome-Wide Association and Transcriptome Analyses Reveal Candidate Genes Underlying Yield-determining Traits in Brassica napus
Source: Front Plant Sci. 2017 Feb 15;8:206. doi: 10.3389/fpls.2017.00206 (PMC5309214; doi:10.3389/fpls.2017.00206)
Supplement: Supplementary file 5 [file Table5.PDF]

Supplementary Material

Genome-Wide Association and Transcriptome Analyses Reveal Candidate Genes Underlying Yield-determining Traits in *Brassica napus*

Kun Lu[1†*], Liu Peng[1,2†], Chao Zhang[1,3], Junhua Lu[1], Bo Yang[1], Zhongchun Xiao[1], Ying Liang[1], Xingfu Xu[1], Cunmin Qu[1], Kai Zhang[1], Liezhao Liu[1], Qinlong Zhu[4], Minglian Fu[5], Xiaoyan Yuan[5], Jiana Li[1*]

*** Correspondence:**

Kun Lu: drlukun@swu.edu.cn
Jiana Li: ljn1950@swu.edu.cn

Supplementary Table S5. Primers designed for qRT-PCR analysis

Gene name	Primer sequence (5 ́–3 ́)	Tm (℃)	AT (℃)	PLC (bp)
Bna.ROP3	F: ACGGAGCCACGGTCAATCTTG	63.0	58	155
	R: GATCCACTTCTTGGAGACATTCTCAT	60.6		
Bna.QRT3	F: GTGGTAGCTACTTAATCAGCCGTC	61.0	58	188
	R: GATGAGAAGGTCTCTGAGAGTGA	58.7		
Bna.PEL7	F: GAATCCTAGACCCGGAACACTTC	60.4	58	249
	R: CTTATCATACCACCACTGCCGG	60.8		
Bna.TAP35	F: TCATCCTCCCGTACTTCCGG	60.8	58	111
	R: CTGAAGAAACCAGCTATCGCAGAG	61.5		
Bna.MYB83	F: CCATGCACATGGACTCTTCCTCATTTTA	63.5	58	107
	R: TTGTTGATATCGGCTATGATCCGGT	62.0		
Bna.BBX20	F: TGTCTCCCATGAGGATAACATTATCAGCA	63.7	58	95
	R: CCCAAAACCCTTGATGATGGTACTGTAAA	63.5		
Bna.BBX15	F: CGGGATGGAACGTTGCCTG	61.4	58	253
	R: CACTTGAGGGTAAACATCCACCAAC	62.0		
Bna.BRK1	F: CAGTAAACGTAGGAATCGCTGTCC	61.5	58	86
	R: GAAGAGACGGCGAACATTGAGAG	61.5		
Bna.LEA3	F: GGAACAACCGCTTCTACCGC	61.4	58	100
	R: GTCTGTAGTAACCGGTTTTAGGATCTG	61.1		
Bna.LRP1	F: GTCTAGCTCGAACAACTCAGGCT	62.2	58	89
	R: TGCATTCCTTCTTCGCCTGGTTTC	63.9		
Bna.PRP17	F: CCACCCCACCACCTCCATA	60.6	58	96
	R: GCGTAGGGGATGGAGTGTATG	60.0		
Bna.bHLH91	F: GAGTTCTCTGTCTTCCCACCTG	60.0	58	280
	R: CGTATGAGATGCAAGAACCAGGCATTATAT	63.5		
Bna.SPL5	F: GTCAAGTTACCAGGAAGAAGAAGAAGTTG	62.2	58	160
	R: CCGGTGATACTGTTTATCCTCTTTCAAAT	61.7		
Bna.SUS2	F: GAAGAGCGTTCCCCGTCACT	62.2	58	77
	R: CATCATAGTCCATTGGAGGATCCTCATTA	62.1		
Bna.ACT7	F: TGGGTTTGCTGGTGACGAT	59.5	58	63
	R: TGCCTAGGACGACCAACAATACT	61.4		
Bna.UBC21	F: CCTCTGCAGCCTCCTCAAGT	61.6	58	77
	R: CATATCTCCCCTGTCTTGAAATGC	59.5		

F, forward primer; R, reverse primer; T, melting temperature; AT, annealing temperature; PLC, product length of cDNA sequences.
