# Supplementary material for: Genome-Wide Association and Transcriptome Analyses Reveal Candidate Genes Underlying Yield-determining Traits in Brassica napus
Source: Front Plant Sci. 2017 Feb 15;8:206. doi: 10.3389/fpls.2017.00206 (PMC5309214; doi:10.3389/fpls.2017.00206)
Supplement: Supplementary file 6 [file Table6.PDF]

frontiers

Supplementary Material

Genome-Wide Association and Transcriptome Analyses Reveal Candidate Genes Underlying Yield-determining Traits in *Brassica napus*

Kun Lu[1†*], Liu Peng[1,2†], Chao Zhang[1,3], Junhua Lu[1], Bo Yang[1], Zhongchun Xiao[1], Ying Liang[1], Xingfu Xu[1], Cunmin Qu[1], Kai Zhang[1], Liezhao Liu[1], Qinlong Zhu[4], Minglian Fu[5], Xiaoyan Yuan[5], Jiana Li[1*]

*** Correspondence:**

Kun Lu: drlukun@swu.edu.cn
Jiana Li: ljn1950@swu.edu.cn

Supplementary Table S6. Summary of SNPs significantly associated with YDTs

Chr.	SNP	Env	Trait	MAF	Allele	*P*-value (MLM)	R^2 (MLM)	*P*-value (GLM)	R^2 (GLM)	Range (bp)	Known loci
A01	rs265	E4	SPP	0.068	T/G	7.22E-05	0.1167	0.00251	0.07025	786674-873224	*qSN.A01-1* (Shi *et al.*, 2015)
A01	rs276	E1	SPP	0.344	C/T	1.86E-05	0.05031	5.55E-21	0.17949		
A01	rs2157	E3	SPP	0.196	G/A	5.47E-05	0.09579	1.68E-07	0.13155	5042605-5187842	
A01	rs140	E4	SPP	0.471	T/C	8.30E-05	0.11506	3.80E-04	0.08896		
A01	rs145	E4	SPP	0.114	T/C	7.37E-05	0.11647	2.79E-04	0.09197	9362727-9395621	
A01	rs156	E4	SPP	0.134	T/C	1.07E-05	0.13942	7.11E-05	0.1051		
A01	rs708	E4	SPP	0.151	A/G	7.74E-05	0.11589	3.75E-04	0.0891	14879746-14943778	
A01	rs673	E4	SPP	0.203	A/G	1.35E-05	0.13662	4.39E-05	0.10968		
A01	rs1222	E1	BPN	0.444	G/A	1.89E-05	0.05092	1.19E-05	0.05063	18466247-18579414	
A01	rs1222	E1	PNP	0.444	G/A	2.75E-05	0.0492	1.46E-05	0.04982		
A02	rs4234	E4	MIY	0.155	C/T	2.10E-05	0.12057	2.46E-06	0.12734	658778-709679	
A02	rs37993	E2	BY	0.371	A/G	7.65E-05	0.04624	5.43E-05	0.04636	3043002-3081720	
A02	rs4600	E2	TSW	0.312	T/C	6.51E-05	0.0433	3.67E-10	0.08678	3775679-3792401	
A02	rs2679	E3	BY	0.170	C/A	1.95E-05	0.11068	1.47E-10	0.18993		
A02	rs3343	E1	BPN	0.243	T/C	2.00E-12	0.12328	1.38E-13	0.12021	17990431-18286433	*cqSY-A2-2* (Zhao *et al.*, 2016)
A02	rs3343	E1	PNP	0.243	T/C	4.70E-12	0.11916	5.94E-13	0.11488		
A02	rs3396	E3	BY	0.450	C/T	8.53E-05	0.09613	6.91E-06	0.10881	18660662-18784334	*cqSY-A2-2* (Zhao *et al.*, 2016)
A02	rs3465	E3	BY	0.202	T/G	6.00E-06	0.12237	2.59E-13	0.23387	19037159-19272015	*cqSW-A2-5* (Zhao *et al.*, 2016)
A02	rs3974	E3	BY	0.196	G/A	7.92E-05	0.08439	1.93E-20	0.32383	23778808-24237427	*cqSW-A2-5* (Zhao *et al.*, 2016)
A02	rs4091	E3	BY	0.217	G/A	6.46E-05	0.09886	1.08E-14	0.25492	24486888-24526395	
A03	rs7475	E2	BY	0.332	A/C	2.52E-05	0.05131	3.85E-05	0.04781	3720445-3782212	*cqSW-A3-1* (Zhao *et al.*, 2016)
A03	rs7481	E2	SPP	0.400	G/A	8.70E-05	0.04811	1.77E-04	0.04315	3808737-3838529	*cqSW-A3-1* (Zhao *et al.*, 2016)
A03	rs8030	E1	BPN	0.056	C/A	7.91E-05	0.0446	3.41E-05	0.0464	7501530-7646487	*cqBY-A3* (Zhao *et al.*, 2016)
A03	rs5320	E3	BY	0.334	G/A	6.96E-05	0.09813	2.81E-08	0.15142	10754837-10758037	*Sil/dm_N3* (Shi *et al.*, 2009)

A03	rs5617	E3	BPN	0.143	T/C	9.07E-05	0.08535	2.40E-05	0.09201	13494779-13589341	*qPN.A03-1* (Shi *et al.*, 2015)
A03	rs5617	E3	PNP	0.143	T/C	9.17E-05	0.08526	2.55E-05	0.09155		
A03	rs5687	E2	MIY	0.221	G/A	2.92E-05	0.04409	8.42E-07	0.05755	14132363-14239734	*qPN.A3-2* (Shi *et al.*, 2009)
A03	rs6515	E4	TSW	0.138	G/A	4.32E-05	0.07935	2.66E-05	0.07685	22079719-22200696	
A03	rs6571	E1	BPN	0.292	T/A	5.51E-06	0.05636	8.26E-06	0.0521	22622257-22660395	
A03	rs6571	E1	PNP	0.292	T/A	6.53E-06	0.05553	7.72E-06	0.05237		
A03	rs7286	E2	MIY	0.122	A/C	2.40E-05	0.05097	1.24E-05	0.05257	27596643-27989279	
A03	rs38231	E2	BPN	0.377	G/T	5.72E-05	0.04667	6.83E-06	0.05393	29073873-29098026	
A03	rs38231	E2	PNP	0.377	G/T	7.94E-05	0.04505	1.41E-05	0.05097		
A04	rs8649	E4	SPP	0.106	G/A	3.19E-05	0.11088	2.53E-04	0.08066		
A04	rs9490	E3	BY	0.390	A/C	4.43E-05	0.10258	9.21E-22	0.35375	2040433-2240433	
A05	rs11582	E4	SPP	0.124	A/C	3.50E-05	0.12529	1.19E-04	0.10017		
A05	rs10508	E3	BY	0.224	C/A	2.83E-06	0.12988	1.61E-17	0.2962	9531586-9731586	
A05	rs10508	E3	SPP	0.224	C/A	2.61E-05	0.10273	2.53E-09	0.16169		
A06	rs14211	E2	SPP	0.410	G/A	1.91E-05	0.0554	5.06E-07	0.06857	2768311-2772149	*cqSY-A6-2* (Zhao *et al.*, 2016)
A06	rs13096	E4	SPP	0.069	A/G	7.54E-05	0.1162	2.79E-04	0.09197	16294441-16498856	*BnGMS583* (Cai *et al.*, 2014)
A06	rs13722	E4	SPP	0.284	T/C	3.97E-05	0.12378	2.50E-05	0.11496	20587640-20820984	*EA06MG11*_4 (Cai *et al.*, 2014)
A06	rs13904	E4	MIPN	0.412	C/T	9.89E-05	0.08829	2.93E-04	0.07421	22642754-22643759	*qSN.A6* (Yang *et al.*, 2015)
A07	rs16016	E3	SPP	0.089	A/C	7.60E-05	0.09272	8.02E-05	0.08477	1664012-1752215	*cqSW-A7-1* (Zhao *et al.*, 2016)
A07	rs22237	E3	SPP	0.364	G/A	3.99E-05	0.09875	3.40E-06	0.10914	2125090-2314808	*cqBY-A7-2* (Zhao *et al.*, 2016)
A07	rs22222	E3	SPP	0.169	T/C	1.08E-05	0.11103	8.82E-05	0.08402		
A07	rs22205	E3	SPP	0.399	C/T	4.72E-05	0.09717	1.58E-05	0.09742		
A07	rs22200	E3	SPP	0.335	A/G	6.38E-05	0.09435	7.91E-05	0.08488	2333587-2496374	
A07	rs22188	E3	SPP	0.125	G/A	1.62E-05	0.1072	1.03E-04	0.08278		
A07	rs22183	E3	SPP	0.146	G/C	4.84E-06	0.11863	3.08E-05	0.09224	2506200-2588895	
A07	rs22242	E3	SPP	0.205	G/A	4.52E-05	0.09757	8.98E-04	0.06549	2686110-2910402	
A07	rs22245	E3	SPP	0.229	A/G	3.98E-06	0.12048	3.23E-05	0.09188		
A07	rs22243	E3	SPP	0.225	T/C	4.14E-06	0.1201	4.72E-05	0.08891		
A07	rs22252	E3	SPP	0.275	A/C	5.77E-06	0.11695	2.78E-06	0.11065		
A07	rs22253	E3	SPP	0.270	C/T	9.51E-06	0.11223	4.04E-06	0.10782		
A07	rs22244	E3	SPP	0.212	C/T	2.36E-05	0.10366	1.72E-04	0.07877		
A07	rs16641	E3	SPP	0.287	T/G	6.15E-05	0.09469	4.71E-05	0.08894	5114965-5237791	
A07	rs15181	E2	BY	0.127	A/G	8.85E-05	0.04557	8.23E-05	0.0446	12315158-12453447	*qPN.A7-1* (Shi *et al.*, 2009)
A07	rs16427	E1	BPN	0.296	A/G	7.82E-05	0.04465	6.52E-04	0.03433	22417611-22617611	*cqBY-A7-2* (Zhao *et al.*, 2016)
A08	rs17921	E3	TSW	0.059	T/C	8.41E-05	0.07205	2.15E-05	0.07823	12464196-12777087	
A08	rs18034	E2	BPN	0.188	C/T	6.25E-05	0.04628	1.60E-04	0.04093	13520923-13598303	
A08	rs18034	E2	PNP	0.188	C/T	3.96E-05	0.04815	6.60E-05	0.04461		
A08	rs18149	E3	BY	0.304	G/T	2.91E-06	0.1296	2.06E-10	0.18749	14438882-14438973	
A08	rs36695	E3	BY	0.200	C/T	2.68E-08	0.1614	4.22E-17	0.2775	17734109-17956051	
A08	rs18241	E1	BPN	0.294	T/C	5.81E-05	0.04596	2.63E-04	0.03809	14822078-15022078	
A08	rs18241	E1	PNP	0.294	T/C	3.60E-05	0.048	1.90E-04	0.03942		
A09	rs21723	E3	BY	0.285	C/T	4.21E-05	0.10308	1.35E-04	0.08466	5037524-5238665	
A09	rs21796	E1	TSW	0.389	T/G	3.08E-05	0.04843	1.94E-11	0.10182	5484355-5747976	
A09	rs19611	E2	SPP	0.436	A/G	2.51E-05	0.05409	1.60E-07	0.07343	9757051-10043720	
A09	rs11020	BLUP	TSW	0.205	C/A	4.95E-05	0.07783	1.78E-04	0.0647	22768463-23063554	
A09	rs21657	E2	BPN	0.244	T/C	5.13E-05	0.04133	2.38E-05	0.04321	29464731-29598718	*EA05MC08*_1 (Cai *et al.*, 2014); *qSW.A9-6* (Shi *et al.*, 2009); *cqSW-A9-1* (Zhao *et al.*, 2016)
A09	rs21657	E2	PNP	0.244	T/C	5.66E-05	0.04077	1.98E-05	0.04394		
A10	rs23256	E2	BPN	0.236	A/C	1.58E-05	0.05244	1.62E-07	0.06897	486257-751636	*cqSW-A10-1* (Zhao *et al.*, 2016)
A10	rs23256	E2	PNP	0.236	A/C	1.22E-05	0.0534	1.85E-07	0.06845		

A10	rs23241	E2	BPN	0.203	C/T	1.85E-06	0.05561	4.62E-06	0.0497	762097-784765	*cqSW-A10-1* (Zhao *et al.*, 2016)
A10	rs23241	E2	PNP	0.203	C/T	3.18E-06	0.05308	1.50E-06	0.05413		
A10	rs23708	E1	TSW	0.105	G/A	9.59E-05	0.04346	4.16E-05	0.0455	9623160-9741176	*cqPMI-A10* (Zhao *et al.*, 2016)
A10	rs23886	E3	BY	0.252	C/T	4.13E-05	0.10327	0.01428	0.04464	10522163-10580420	*cqPMI-A10* (Zhao *et al.*, 2016)
A10	rs23911	E3	BY	0.264	A/C	8.23E-05	0.09648	0.19825	0.01994	10684625-10695208	*cqPMI-A10* (Zhao *et al.*, 2016)
A10	rs14614	E4	SPP	0.304	G/A	4.78E-05	0.12159	2.50E-05	0.11498	14240793-14241120	
A10	rs22562	E1	SPP	0.117	C/T	8.23E-05	0.04383	1.14E-08	0.07774	14264189-14329378	
A10	rs22805	E3	BY	0.190	G/A	1.22E-05	0.1153	5.88E-22	0.35627	15604598-15669763	
C01	rs44041	E3	BY	0.203	A/G	5.30E-08	0.1703	2.51E-27	0.42205	15864962-16351149	*EA09MC07*_12 (Cai *et al.*, 2012)
C01	rs44041	E3	SPP	0.203	A/G	1.58E-06	0.12926	3.87E-13	0.22113		
C03	rs34018	E4	SPP	0.052	C/T	6.17E-05	0.10344	3.62E-05	0.07511	1446387-1551844	
C03	rs33989	E2	PNP	0.221	G/T	6.87E-05	0.0457	6.37E-08	0.11497	1834216-2321120	
C03	rs47973	E2	SPP	0.428	C/A	9.90E-06	0.05857	7.57E-06	0.05696	6208800-6279695	*qPN-LP3-C3a* (Shi *et al.*, 2013)
C03	rs40655	E4	SPP	0.162	A/C	8.02E-05	0.11546	2.11E-04	0.09466	8130967-8956388	
C03	rs28065	E4	SPP	0.060	C/G	3.89E-06	0.13489	0.00224	0.06007		
C03	rs28062	E4	SPP	0.063	T/C	4.38E-06	0.13352	0.00347	0.05588		
C03	rs28070	E3	BY	0.367	G/A	5.87E-05	0.09981	7.86E-15	0.25699	13854748-13992927	
C03	rs44581	E2	MIPN	0.089	G/T	7.58E-05	0.04292	8.10E-07	0.0591	25192580-25519066	
C03	rs42595	E4	SPP	0.132	A/G	7.07E-05	0.11696	5.36E-04	0.08559	28713711-29122413	*cqPMI-C3* (Zhao *et al.*, 2016)
C03	rs42583	E3	BY	0.199	G/A	5.35E-05	0.10072	4.20E-18	0.30441	29823811-30173527	*cqPMI-C3* (Zhao *et al.*, 2016)
C03	rs30540	E3	BY	0.013	G/A	3.96E-09	0.18065	3.49E-21	0.33375	46101407-46453312	
C03	rs17613	E3	TSW	0.149	C/T	4.15E-05	0.07719	2.38E-06	0.09221	51969206-52357539	
C03	rs24243	E3	BY	0.149	G/T	3.47E-05	0.0921	0.36395	0.00868	44526168-44726168	
C04	rs26877	E2	SPP	0.315	T/C	8.70E-05	0.04811	6.32E-04	0.03746	82492-368607	
C04	rs43638	E4	SPP	0.234	T/G	2.60E-05	0.12881	2.69E-04	0.09231	14883588-15179528	
C04	rs32756	E3	BY	0.428	A/C	4.87E-05	0.10165	3.50E-09	0.16694	18333781-18833673	
C04	rs35385	E3	BY	0.263	C/T	7.34E-05	0.0976	2.32E-13	0.23461	33855498-33887045	
C04	rs8444	E4	BPN	0.277	G/T	9.01E-06	0.10482	4.19E-07	0.1194	38869747-38928048	
C04	rs8444	E4	PNP	0.277	G/T	3.08E-05	0.09348	2.96E-06	0.10437		
C04	rs35245	E1	BPN	0.069	T/C	1.13E-07	0.07363	3.12E-07	0.06507	42450406-42860566	
C04	rs35245	E1	PNP	0.069	T/C	3.30E-07	0.06876	1.68E-06	0.05845		
C04	rs35690	E3	TSW	0.373	T/A	1.93E-05	0.08276	3.17E-07	0.10476	44916733-45068789	
C04	rs42510	E4	SPP	0.329	A/C	3.09E-05	0.12677	0.00213	0.0719	47490243-47735836	
C05	rs44626	E2	SPP	0.376	G/A	7.75E-05	0.04866	3.71E-04	0.03985	10802240-11040280	*cqSW-C5* (Zhao *et al.*, 2016)
C05	rs52078	E3	BY	0.017	C/T	2.36E-07	0.13991	4.78E-14	0.23234	37499848-37652652	
C06	rs50722	E4	BY	0.158	C/T	5.05E-05	0.11116	0.00142	0.07075	3480960-3757565	
C06	rs36347	E3	BY	0.201	C/A	2.89E-05	0.0938	4.61E-17	0.27694	12052153-12534523	
C06	rs36347	E3	SPP	0.201	C/A	4.09E-05	0.08632	1.46E-07	0.12103		
C06	rs4658	E3	BY	0.358	T/C	3.41E-05	0.10514	7.73E-15	0.25709	16266277-16679279	
C06	rs42292	E1	BPN	0.208	A/G	2.99E-06	0.05907	1.85E-06	0.05806	30202408-30337696	*cqSY-C6-2* (Zhao *et al.*, 2016)
C06	rs42292	E1	PNP	0.208	A/G	6.45E-06	0.05559	4.90E-06	0.05419		
C06	rs49349	E2	SPP	0.392	T/A	7.87E-05	0.04859	1.28E-06	0.06459	31278885-32026740	*qSN.C06-2* (Shi *et al.*, 2015); *cqSW-C6-3* and *cqSY-C6-3* (Zhao *et al.*, 2016)
C06	rs45027	E2	MIPN	0.184	C/T	4.40E-05	0.04522	1.75E-06	0.05617	33566777-33749111	*qSN.C06-2* (Shi *et al.*, 2015); *cqSW-C6-3* and *cqSY-C6-3* (Zhao *et al.*, 2016)
C06	rs39010	E3	BPN	0.000	C/C	5.28E-05	0.06505	8.23E-06	0.0763	35728070-36178549	
C08	rs45939	E4	SPP	0.271	C/T	9.31E-05	0.11371	0.00135	0.07644	4546027-4866240	
C08	rs34198	E4	SPP	0.280	G/A	5.05E-05	0.12094	7.68E-04	0.08206	4993688-5462228	
C08	rs38351	E2	MIY	0.000	G/G	4.80E-05	0.03476	4.48E-05	0.03464	11972821-12326545	
C08	rs35139	E2	SPP	0.437	A/C	3.16E-05	0.05298	2.05E-05	0.05264	27018598-27019668	

C08	rs31484	E4	BY	0.105	G/A	6.56E-05	0.10834	6.52E-09	0.17625	29993033-30248637	*cqSW-C8-1* (Zhao *et al.*, 2016)
C08	rs29994	E4	MIPN	0.381	A/T	7.45E-05	0.09088	4.76E-04	0.07035	32199279-32209089	*cqSW-C8-1* (Zhao *et al.*, 2016)
C08	rs29889	E3	SPP	0.098	G/T	7.31E-05	0.09308	4.29E-06	0.10737	32976567-33073389	
C09	rs44262	E3	TSW	0.335	T/C	3.45E-05	0.07853	2.05E-05	0.07854	23116889-23117415	
C09	rs3118	E3	TSW	0.316	C/A	1.10E-05	0.0869	2.86E-06	0.09104		
C09	rs39414	E3	BY	0.155	C/A	6.46E-06	0.10796	4.72E-19	0.30495	34334321-34506204	
C09	rs39414	E3	SPP	0.155	C/A	4.47E-05	0.08554	6.77E-10	0.15891		
C09	rs44016	E4	SPP	0.100	A/C	7.89E-06	0.14309	3.20E-05	0.11266	35650406-36027396	

Chr, chromosome; MAF, minor allele frequency; MIPN, main inflorescence pod number; BPN, branch pod number; PNP, pod number per plant; SPP, seed number per pod; TSW, thousand seed weight; MIY, main inflorescence yield; BY, branch yield.
R^2 is the percentage of phenotypic variance explained by SNP.
