# Supplementary material for: Genome-Wide Association and Transcriptome Analyses Reveal Candidate Genes Underlying Yield-determining Traits in Brassica napus
Source: Front Plant Sci. 2017 Feb 15;8:206. doi: 10.3389/fpls.2017.00206 (PMC5309214; doi:10.3389/fpls.2017.00206)
Supplement: Supplementary file 7 [file Table7.PDF]

Supplementary Material

Genome-Wide Association and Transcriptome Analyses Reveal Candidate Genes Underlying Yield-determining Traits in *Brassica napus*

Kun Lu[1†*], Liu Peng[1,2†], Chao Zhang[1,3], Junhua Lu[1], Bo Yang[1], Zhongchun Xiao[1], Ying Liang[1], Xingfu Xu[1], Cunmin Qu[1], Kai Zhang[1], Liezhao Liu[1], Qinlong Zhu[4], Minglian Fu[5], Xiaoyan Yuan[5], Jiana Li[1*]

*** Correspondence:**

Kun Lu: drlukun@swu.edu.cn
Jiana Li: ljn1950@swu.edu.cn

Supplementary Table S7. Summary of significantly enriched gene ontology (GO) terms.

Sample name	BP		MF		CC		Total	
	Up	Down	Up	Down	Up	Down	Up	Down
cSt	155	229	35	48	44	15	234	292
cLe	459	280	58	93	93	12	610	385
cBM	39	92	10	42	0	7	49	141
cBB	19	147	1	51	18	10	38	208
cSPM	63	182	9	38	29	22	101	242
cSPB	252	171	27	36	6	25	285	232
cSM	106	507	15	4	35	75	156	586
cSB	103	508	41	59	7	77	151	644
ySt	389	54	147	22	43	22	579	98
yLe	45	84	3	20	31	12	79	116
yBM	31	1	3	6	0	0	34	7
yBB	126	0	26	0	7	0	159	0
ySPM	166	292	117	72	14	41	297	405
ySPB	229	35	39	21	39	0	307	56
ySM	75	8	18	4	42	2	135	14
ySB	62	0	16	0	17	17	95	17

BP, biological process; MF, molecular function; CC, cell component.

First letters (c and y) in the sample names represent the cultivation region Chongqing and Yunnan, respectively. SPP, silique pericarps on the primary branch; BP, buds on the primary branch; SP, seeds harvested 20 days after flowering on the primary branch; Le, leaves; St, stems; SPM, silique pericarps on the main inflorescence; BM, buds on the main inflorescence; SM, seeds harvested 20 days after flowering on the main inflorescence.
