# Supplementary material for: Genome-Wide Association and Transcriptome Analyses Reveal Candidate Genes Underlying Yield-determining Traits in Brassica napus
Source: Front Plant Sci. 2017 Feb 15;8:206. doi: 10.3389/fpls.2017.00206 (PMC5309214; doi:10.3389/fpls.2017.00206)
Supplement: Supplementary file 8 [file Table8.PDF]

frontiers

Supplementary Material

Genome-Wide Association and Transcriptome Analyses Reveal Candidate Genes Underlying Yield-determining Traits in *Brassica napus*

Kun Lu[1†*], Liu Peng[1,2†], Chao Zhang[1,3], Junhua Lu[1], Bo Yang[1], Zhongchun Xiao[1], Ying Liang[1], Xingfu Xu[1], Cunmin Qu[1], Kai Zhang[1], Liezhao Liu[1], Qinlong Zhu[4], Minglian Fu[5], Xiaoyan Yuan[5], Jiana Li[1*]

* Correspondence:

Kun Lu: drlukun@swu.edu.cn
Jiana Li: ljn1950@swu.edu.cn

Supplementary Table S8. Candidate genes retrieved from the 21 important HBs

HB blocks	Candidate genes	*Arabidopsis* ortholog	Function annotation	Fold change															
				cSt	cLe	cBM	cBB	cSPM	cSPB	cSM	cSB	ySt	yLe	yBM	yBB	ySPM	ySPB	ySM	ySB
1	BnaA01g01460D	AT4G36140	disease resistance protein (TIR-NBS-LRR class)	0.70	0.02	0.55	-0.37	-0.05	0.57	-0.75	0.20	0.59	-0.55	-0.21	-0.62	-0.17	-0.19	-0.48	0.14
1	BnaA01g01470D	AT4G36130	Ribosomal protein L2 family	-0.24	0.23	-0.16	-0.05	-0.56	0.00	0.09	-0.09	-0.71	-0.33	-0.37	0.12	-0.16	0.20	-0.01	0.04
1	BnaA01g01480D	AT4G36120	Plant protein of unknown function (DUF869)	0.01	-0.91	-0.24	-1.97	0.22	-0.54	0.70	-0.38	0.13	1.00	0.18	0.16	-0.14	0.10	0.85	-1.08
1	BnaA01g01490D	AT4G36110	SAUR-like auxin-responsive protein family	-0.45	0.00	0.00	0.00	0.00	0.00	0.00	0.00	-0.10	0.00	-0.44	0.00	0.00	0.00	0.00	0.00
1	BnaA01g01500D	AT4G36105	unknown protein	0.22	0.36	-0.24	0.23	0.33	0.04	1.04	0.57	-0.76	-0.28	0.03	0.01	0.25	-0.53	0.04	-0.06
1	BnaA01g01510D	AT4G36090	oxidoreductase, 2OG-Fe(II) oxygenase family protein	1.68	0.20	0.52	-2.14	1.72	0.35	0.00	0.60	0.00	inf	0.44	0.03	-0.04	0.00	inf	inf
1	BnaA01g01520D	AT4G36080	phosphotransferases, alcohol group as acceptor	-0.22	-0.87	0.36	0.11	-0.07	-0.14	-0.46	-0.04	0.66	-0.15	-0.42	-0.15	0.33	-0.46	-0.06	-0.01
1	BnaA01g01530D			0.00	0.00	0.00	0.00	0.00	0.00	0.00	0.00	0.00	0.00	0.00	0.00	0.00	0.00	0.00	0.00
1	BnaA01g01540D	AT4G36050	endonuclease/exonuclease/phosphatase family protein	-0.50	-0.49	0.87	0.00	0.06	0.27	-0.19	-0.11	-0.05	-0.02	0.49	0.26	0.26	-0.77	-0.09	-0.45
1	BnaA01g01550D	AT4G36040	Chaperone DnaJ-domain superfamily protein	0.02	-0.93	0.17	-0.68	0.10	0.48	-1.18	-0.05	-0.32	0.56	-0.23	0.30	0.18	-0.21	0.60	-0.08
1	BnaA01g01560D	AT4G36020	cold shock domain protein 1 (CSDP1)	0.27	0.44	0.13	-0.28	-0.26	0.21	-0.53	0.21	-0.07	-0.06	-0.08	0.20	-0.07	0.71	0.43	0.70
1	BnaA01g01570D	AT4G36010	Pathogenesis-related thaumatin superfamily protein	0.96	2.29	0.91	-1.29	-0.23	0.43	-1.58	1.19	-0.88	-0.04	-0.19	0.01	-0.79	-0.06	0.07	0.41
1	BnaA01g01580D	AT4G35987	S-adenosyl-L-methionine-dependent methyltransferases superfamily protein	0.02	-0.22	0.21	0.11	-0.56	-0.04	0.88	0.62	-0.61	0.47	0.15	-0.58	-0.71	-0.01	-0.26	-0.70
1	BnaA01g01590D	AT4G35980	unknown protein	0.54	0.76	0.63	0.14	-0.25	0.17	-0.17	-0.14	0.30	0.46	0.11	-0.26	0.34	-0.74	-0.69	-1.26
1	BnaA01g01600D	AT4G35970	ascorbate peroxidase 5 (APX5)	0.38	0.16	-0.29	inf	0.00	-0.99	0.00	0.43	0.07	0.13	-0.03	0.00	-2.94	-0.08	-0.41	0.00
1	BnaA01g01610D	AT2G17800	RHO-RELATED GTPASES FROM PLANTS 3 (ROP3)	0.00	0.00	-0.99	0.00	0.00	0.00	inf	-0.82	0.00	0.00	0.08	0.50	0.00	0.00	inf	0.00
1	BnaA01g01620D	AT4G35940	unknown protein	0.23	-0.98	-0.27	-0.43	0.10	0.83	-1.19	-0.35	-0.50	-0.36	0.00	0.14	0.72	-0.17	-0.27	-0.12
1	BnaA01g01630D	AT4G35920	mid1-complementing activity 1 (MCA1)	0.12	0.19	-0.35	-0.04	-0.49	0.34	0.12	-0.11	-0.13	0.55	0.07	-0.49	0.16	1.02	-0.65	-0.02
1	BnaA01g01640D	AT4G35900	FD	-1.20	1.15	0.00	0.00	1.84	1.12	0.11	-1.20	-1.71	0.00	-1.23	0.80	1.45	0.00	-1.69	inf
2	BnaA01g10130D			0.25	0.96	-0.09	0.00	-0.83	inf	2.46	-0.18	0.00	0.21	-0.30	-0.08	0.00	0.00	0.15	-0.49
2	BnaA01g10140D			-1.80	-0.58	0.07	-0.97	0.07	-0.56	-0.77	0.10	-0.85	1.36	-0.20	-0.17	-0.56	0.04	0.23	-0.48
2	BnaA01g10150D	AT4G18870	E2F/DP family winged-helix DNA-binding domain	0.00	0.00	0.66	0.00	inf	0.00	-1.07	-0.26	0.00	0.00	-0.35	0.00	0.45	0.00	-1.83	0.00
2	BnaA01g10160D	AT4G19640	ARA7	0.66	0.67	0.68	-0.16	0.54	0.37	-0.18	0.63	0.19	0.02	0.17	-0.09	-0.15	-0.19	-0.14	-0.63

2	BnaA01g10170D	AT4G19645	TRAM, LAG1 and CLN8 (TLC) lipid-sensing domain containing protein	-0.45	-0.90	-0.29	-0.13	0.52	0.22	0.37	-1.63	0.73	0.04	-0.25	0.46	1.07	0.01	-0.21	-0.78
2	BnaA01g10180D			0.00	0.00	0.00	0.00	0.00	0.00	0.00	0.00	0.00	0.00	0.00	0.00	0.00	0.00	0.00	0.00
2	BnaA01g10190D			0.00	0.00	0.00	0.00	0.00	0.00	0.00	0.00	0.00	0.00	0.00	0.00	0.00	0.00	0.00	0.00
2	BnaA01g10200D	AT4G19650	Mitochondrial transcription termination factor family protein	-0.37	0.35	-0.11	0.15	0.03	0.11	-0.10	-0.22	-0.17	-0.22	-0.62	0.06	-0.28	-0.47	-0.09	-0.10
2	BnaA01g10210D	AT3G18100	myb domain protein 4r1 (MYB4R1)	0.00	1.26	1.50	1.52	0.00	-0.95	1.11	0.66	inf	1.72	0.82	-0.45	0.65	-0.38	-0.48	-0.38
2	BnaA01g10220D	AT4G19660	NPR1-like protein 4 (NPR4)	0.15	-0.20	0.18	0.21	0.09	-0.18	-0.07	-0.06	0.16	-0.09	0.48	0.01	-0.01	-0.18	0.20	-0.13
2	BnaA01g10230D	AT4G19670	RING/U-box superfamily protein	-0.15	-0.43	0.06	0.04	0.15	0.04	-0.43	0.05	0.18	0.24	0.12	-0.02	0.17	-0.25	-0.37	-0.32
2	BnaA01g10240D	AT4G19680	iron regulated transporter 2 (IRT2)	0.00	0.00	inf	1.25	inf	0.00	0.00	inf	0.00	0.00	inf	inf	0.00	0.00	0.00	0.00
2	BnaA01g10250D	AT3G51670	SEC14 cytosolic factor family protein / phosphoglyceride transfer family protein	0.00	0.00	0.00	0.00	0.00	0.00	0.00	0.00	0.00	0.00	0.00	0.00	0.00	0.00	0.00	0.00
2	BnaA01g10260D	AT4G19850	lectin-related	-1.06	-0.11	-0.33	0.00	0.70	1.64	0.00	0.78	2.00	-0.53	0.00	-2.27	0.97	0.07	0.00	0.00
2	BnaA01g10270D	AT4G19860	alpha/beta-Hydrolases superfamily protein	0.13	-0.15	-0.16	0.27	0.22	0.09	0.17	-0.08	-0.03	-0.25	-0.06	0.13	0.21	-0.43	-0.03	-0.13
2	BnaA01g10280D	AT1G80070	ABNORMAL SUSPENSOR 2 (SUS2)	-0.13	-0.62	0.12	-0.05	-0.10	-0.22	-0.33	-0.12	0.43	-0.54	-0.28	0.03	0.17	-0.02	0.03	0.14
2	BnaA01g10290D	AT4G19890	Pentatricopeptide repeat (PPR-like) superfamily protein	-0.61	-0.16	-0.64	-0.87	-0.68	-0.40	0.10	-0.45	0.28	-1.00	0.30	-0.12	0.55	-0.84	-0.83	-0.24
2	BnaA01g10300D	AT4G19890	Pentatricopeptide repeat (PPR-like) superfamily protein	-2.55	-0.20	0.13	1.61	0.37	1.26	-1.15	-0.11	0.00	0.36	-0.90	0.41	-0.45	0.00	0.35	0.57
2	BnaA01g10310D	AT4G19960	K+ uptake permease 9 (KUP9)	-0.66	-0.89	0.22	1.42	0.27	0.75	0.65	-1.25	0.21	0.10	0.90	1.36	-0.05	-0.52	0.67	-0.23
2	BnaA01g10320D	AT4G19970	CONTAINS InterPro DOMAIN/s: Nucleotide-diphospho-sugar transferase, predicted (InterPro:IPR005069)	-0.50	-2.47	-0.98	inf	-0.03	-0.63	inf	-0.56	0.88	-2.24	0.00	0.00	-0.38	-0.35	0.54	-2.94
2	BnaA01g10330D	AT4G19980	unknown protein	0.00	0.00	0.39	0.00	0.00	0.00	-0.69	1.83	0.00	0.00	0.04	1.36	0.00	0.00	-2.48	-1.16
2	BnaA01g10340D	AT4G19985	Acyl-CoA N-acyltransferases (NAT) superfamily protein	-0.45	0.49	0.52	-0.40	0.29	0.32	-0.75	0.37	0.65	-0.33	-0.10	-0.42	-0.54	-0.22	-0.03	-0.32
2	BnaA01g10350D	AT4G20000	VQ motif-containing protein	-0.45	0.00	0.00	0.00	0.06	-0.77	0.00	0.00	0.00	0.00	0.00	0.00	0.43	0.00	0.00	0.00
2	BnaA01g10360D	AT4G20020	unknown protein	-0.03	-0.16	0.02	0.45	0.23	-0.19	0.78	-0.08	-0.21	0.23	-0.31	-0.01	-0.04	0.05	-0.22	0.08
2	BnaA01g10370D	AT4G20030	RNA-binding (RRM/RBD/RNP motifs) family protein	-0.69	0.06	0.25	0.32	0.31	-0.06	0.66	-0.09	0.13	-0.26	0.33	-0.30	-0.17	-0.05	0.02	0.20
2	BnaA01g10380D	AT4G20040	Pectin lyase-like superfamily protein	0.61	0.00	-0.16	0.08	-0.63	2.73	-1.02	0.86	-0.41	1.07	-0.34	0.02	-1.31	2.53	0.57	0.33
2	BnaA01g10390D	AT4G20050	QUARTET 3 (QRT3)	0.97	0.35	0.16	-0.95	-0.42	0.27	-1.41	0.21	-0.88	-0.62	0.48	0.11	3.20	1.06	-0.25	0.77
3	BnaA01g26410D	AT3G17540	F-box and associated interaction domains-containing protein	0.00	0.00	inf	0.00	0.00	0.00	0.00	inf	0.00	inf	0.00	inf	0.00	0.00	-1.52	inf
3	BnaA01g26420D	AT3G17530	F-box and associated interaction domains-containing protein	0.00	0.00	0.64	0.29	0.00	0.00	0.00	-1.88	0.00	1.04	-0.43	-0.55	inf	0.00	-1.70	1.31
3	BnaA01g26430D	AT3G18830	polyol/monosaccharide transporter 5 (PMT5)	-1.32	-1.89	-0.86	0.09	-0.15	-0.62	0.49	-1.09	1.66	0.18	-0.25	0.18	0.58	0.72	3.46	-1.93
3	BnaA01g26440D	AT1G51720	Amino acid dehydrogenase family protein	0.00	0.96	0.58	0.00	3.28	0.99	0.38	-2.51	-1.14	0.00	-0.40	-1.91	-1.57	-1.61	-0.99	-2.25
3	BnaA01g26450D	AT3G17300	unknown protein	0.00	0.00	0.00	0.00	0.00	0.00	0.00	0.00	0.00	0.00	0.00	0.00	0.00	0.00	0.00	0.00
3	BnaA01g26460D	AT3G17530	F-box and associated interaction domains-containing protein	0.00	0.00	0.00	0.00	0.00	0.00	0.00	0.00	0.00	0.00	0.00	0.00	0.00	0.00	0.00	0.00
3	BnaA01g26470D	AT3G18820	RAB GTPase homolog G3F (RAB7B)	-0.04	-0.10	-0.07	0.03	0.24	0.19	0.47	0.20	-0.76	0.49	-0.34	0.58	-0.15	-0.06	0.01	-0.46
3	BnaA01g26480D	AT3G18810	Protein kinase superfamily protein	-1.45	-1.66	-2.90	0.00	inf	0.11	0.00	-4.11	-0.49	2.19	1.23	5.89	0.00	-1.18	-1.11	0.00
3	BnaA01g26490D	AT3G18790	FUNCTIONS IN: molecular_function unknown	0.10	1.27	-0.05	1.24	1.65	0.00	0.39	0.45	0.00	-0.11	1.77	1.42	1.23	0.28	0.00	0.00
3	BnaA01g26500D	AT3G18780	actin 2 (ACT2)	-0.69	-0.77	-0.63	-1.15	-0.52	-1.08	-0.76	-0.70	-0.25	-0.47	-0.33	0.18	0.50	0.01	0.92	0.02
3	BnaA01g26510D	AT1G49230	RING/U-box superfamily protein	-2.88	0.00	0.00	inf	0.00	0.00	0.00	inf	-2.06	inf	-0.21	0.00	0.00	0.00	0.00	0.00
3	BnaA01g26520D	AT3G18773	RING/U-box superfamily protein	1.18	-2.31	0.00	-0.34	0.00	inf	-2.03	0.00	0.76	0.04	inf	inf	inf	0.00	0.73	0.00
3	BnaA01g26530D	AT5G57030	LUTEIN DEFICIENT 2 (LUT2)	inf	0.26	inf	0.23	0.00	-1.28	-1.03	0.19	inf	inf	-1.39	-0.32	0.00	-0.96	-0.96	-0.58
4	BnaA02g24670D	AT5G46780	VQ motif-containing protein	-2.35	-3.86	-1.52	0.28	-0.61	-0.61	1.45	-4.13	-0.51	-1.49	1.01	-0.50	-0.48	-0.02	-0.68	inf
4	BnaA02g24680D	AT5G46795	microspore-specific promoter 2 (MSP2)	0.00	0.00	-0.37	0.00	0.00	0.00	-0.05	-0.53	0.00	0.00	0.04	-0.02	0.00	0.00	inf	inf
4	BnaA02g24690D	AT5G46800	A BOUT DE SOUFFLE (BOU)	-0.32	0.36	-0.56	-0.63	-0.14	-0.94	0.08	-0.41	0.47	-0.03	-0.19	-0.06	-0.57	0.75	0.28	0.23
4	BnaA02g24700D	AT5G46820	Protein of Unknown Function (DUF239)	0.00	0.00	0.00	0.00	0.00	0.00	0.00	0.00	0.00	0.00	0.00	0.00	0.00	0.00	0.00	0.00
4	BnaA02g24710D	AT5G04120	Phosphoglycerate mutase family protein	0.00	0.00	0.00	0.00	0.00	0.00	0.00	0.00	0.00	0.00	0.00	0.00	0.00	0.00	0.00	0.64
4	BnaA02g24720D	AT3G01780	TPLATE	0.00	-0.91	-0.63	-0.08	-0.28	-0.55	-0.29	-0.89	0.02	-0.15	-0.28	0.03	0.02	0.24	-0.14	-0.12
4	BnaA02g24730D	AT1G29720	Leucine-rich repeat transmembrane protein kinase	-2.13	-0.55	-0.76	-0.78	-0.58	-0.77	0.00	-0.76	1.37	0.00	-0.35	-1.25	-0.45	-0.05	inf	-1.38
4	BnaA02g24740D	AT1G29720	Leucine-rich repeat transmembrane protein kinase	0.00	0.00	0.00	0.00	0.00	0.00	0.00	0.00	0.00	0.00	0.00	0.00	0.00	0.00	0.00	0.00

4	BnaA02g24750D	AT1G29720	Leucine-rich repeat transmembrane protein kinase	0.00	0.00	0.00	0.00	0.00	-2.41	0.00	inf	inf	0.00	0.00	0.00	0.00	0.25	0.00	inf
4	BnaA02g24760D			0.00	0.00	-0.14	0.00	0.00	0.00	0.00	0.38	0.00	inf	0.35	-0.68	0.00	0.00	inf	0.00
4	BnaA02g24770D	AT5G46860	VACUOLAR MORPHOLOGY 3 (VAM3)	-2.39	-3.45	-1.95	0.58	-2.27	-1.66	0.53	-1.15	-0.94	-1.02	-1.73	-0.19	0.85	-0.13	-0.26	0.04
4	BnaA02g24780D	AT5G46910	Transcription factor jumonji (jmj) family protein / zinc finger (C5HC2 type) family protein	0.16	-0.71	-0.20	0.11	-0.24	0.63	0.11	0.07	-1.08	-0.87	0.16	-0.26	0.45	0.63	0.10	-0.07
4	BnaA02g24790D	AT5G46920	Intron maturase, type II family protein	0.00	0.00	0.00	0.00	0.00	0.00	0.00	0.00	0.00	0.00	0.00	0.00	0.00	0.00	0.00	0.00
4	BnaA02g24800D	AT5G46940	Plant invertase/pectin methylesterase inhibitor superfamily protein	0.00	inf	-2.74	0.00	0.00	0.00	0.00	-4.45	-1.02	inf	1.84	3.03	0.00	0.00	0.00	-1.36
4	BnaA02g24810D	AT5G47600	HSP20-like chaperones superfamily protein	0.00	inf	2.39	0.00	0.00	0.00	0.00	2.45	0.00	0.00	1.07	-0.66	0.00	0.00	inf	inf
4	BnaA02g24820D	AT5G47590	Heat shock protein HSP20/alpha crystallin family	1.15	0.66	-1.09	0.46	-1.10	0.63	1.00	0.87	0.00	0.00	0.00	-1.65	0.30	-0.24	-0.64	0.67
4	BnaA02g24830D	AT5G47590	Heat shock protein HSP20/alpha crystallin family	-0.18	-0.01	1.78	0.37	0.58	0.90	-0.62	1.34	-0.01	0.11	-0.95	0.29	-0.17	0.43	-0.39	1.73
4	BnaA02g24840D	AT5G46960	Plant invertase/pectin methylesterase inhibitor superfamily protein	0.00	0.00	0.00	-1.54	0.00	0.00	-2.10	0.00	0.00	0.00	0.00	inf	0.00	-1.27	0.25	1.55
4	BnaA02g24850D	AT5G47590	Heat shock protein HSP20/alpha crystallin family	inf	inf	0.00	0.24	0.70	2.31	0.00	0.44	-0.03	-1.00	-0.42	-0.51	-2.97	1.85	inf	-2.25
4	BnaA02g24860D	AT5G02960	Ribosomal protein S12/S23 family protein	0.00	0.00	0.00	0.00	0.00	0.00	0.00	0.00	0.00	0.00	0.00	0.00	0.00	0.00	0.00	0.00
4	BnaA02g24870D	AT1G54620	Plant invertase/pectin methylesterase inhibitor superfamily protein	0.00	1.08	-1.25	inf	1.31	-0.27	inf	-1.64	0.00	0.00	0.42	2.87	0.00	0.00	0.00	0.00
4	BnaA02g24880D	AT5G47000	Peroxidase superfamily protein	0.00	-1.88	-2.87	inf	0.00	0.00	0.00	0.00	0.00	0.00	1.39	inf	0.00	0.00	0.14	0.00
4	BnaA02g24890D	AT5G47010	LOW-LEVEL BETA-AMYLASE 1 (LBA1)	0.08	-0.65	-0.25	0.10	-0.17	0.01	-0.23	-0.43	0.41	-0.31	-0.40	0.04	0.34	0.25	-0.02	0.04
4	BnaA02g24900D	AT5G47030	ATPase, F1 complex, delta/epsilon subunit	0.02	0.34	-0.55	-0.17	-0.33	-0.16	0.20	-0.21	-0.48	0.60	0.09	0.22	-0.07	0.50	0.00	-0.05
4	BnaA02g24910D	AT5G47050	SBP (S-ribonuclease binding protein) family protein	-0.72	1.93	-0.02	0.43	-1.47	-1.00	-0.18	-0.34	0.60	0.87	0.46	0.93	0.35	0.12	0.64	-0.52
4	BnaA02g24920D	AT2G04045	Defensin-like (DEFL) family protein	0.00	0.00	0.00	0.00	0.00	0.00	0.00	0.00	0.00	0.00	0.00	0.00	0.00	0.00	-1.80	0.00
4	BnaA02g24930D	AT4G04540	cysteine-rich RLK (RECEPTOR-like protein kinase) 39 (CRK39)	-0.99	-1.65	-1.05	0.19	0.55	-0.17	0.86	-1.07	1.03	-0.21	2.45	0.00	1.22	0.66	inf	inf
4	BnaA02g33210D	AT5G62160	zinc transporter 12 precursor (ZIP12)	0.00	0.00	inf	0.00	0.00	0.00	0.00	0.00	0.00	0.00	0.47	1.20	inf	0.00	0.00	0.00
4	BnaA02g33220D			inf	0.00	0.00	0.00	0.00	inf	0.00	0.00	0.00	-2.42	0.00	0.00	0.00	0.00	0.00	0.00
4	BnaA02g33230D	AT5G62165	AGAMOUS-like 42 (AGL42)	-0.12	-0.04	-0.05	0.18	-0.10	-0.59	0.08	0.03	0.48	-0.03	-0.14	0.11	-0.19	0.67	inf	inf
4	BnaA02g33240D	AT5G62180	carboxyesterase 20 (CXE20)	0.00	-2.05	-1.82	0.00	-3.25	-0.03	0.00	1.76	inf	0.00	0.00	0.00	0.00	-0.38	-0.77	0.84
4	BnaA02g33250D			0.00	-0.46	-1.43	0.00	-0.30	0.00	1.11	inf	0.00	0.00	0.00	inf	0.00	inf	-0.81	inf
4	BnaA02g33260D	AT5G62180	carboxyesterase 20 (CXE20)	0.00	-1.52	0.00	0.00	0.00	0.00	-0.01	inf	0.00	inf	inf	0.00	0.00	0.00	-1.93	0.00
4	BnaA02g33270D	AT5G62180	carboxyesterase 20 (CXE20)	0.11	-0.62	-0.29	-0.74	-0.17	-0.11	-0.51	0.21	-0.32	0.21	-0.32	0.57	-0.36	0.91	0.27	0.89
4	BnaA02g33280D			0.00	0.00	0.00	0.00	0.00	0.00	0.00	0.00	0.00	0.00	0.00	0.00	0.00	0.00	0.00	0.00
4	BnaA02g33290D	AT5G62180	carboxyesterase 20 (CXE20)	0.00	0.00	0.00	0.00	inf	0.00	0.00	0.00	0.00	0.00	0.00	inf	0.00	0.00	0.00	0.00
4	BnaA02g33300D	AT5G62180	carboxyesterase 20 (CXE20)	0.00	0.00	2.10	0.00	0.65	0.36	0.00	-0.42	0.00	0.00	-0.28	-0.48	-1.67	inf	0.00	0.00
4	BnaA02g33310D	AT5G62200	Embryo-specific protein 3, (ATS3)	-0.42	0.02	0.14	0.16	-0.37	0.31	-0.50	0.00	-0.25	0.48	-0.11	0.28	-0.01	-0.13	0.02	-0.44
4	BnaA02g33320D	AT5G62260	AT hook motif DNA-binding family protein	0.00	-3.13	-0.72	1.14	-4.02	0.00	1.12	-0.84	-0.50	-1.10	-0.31	0.30	1.12	0.70	-0.35	-0.51
4	BnaA02g33330D	AT5G62270	FUNCTIONS IN: molecular_function unknown	0.00	inf	2.36	0.17	inf	0.00	-2.12	-1.05	0.00	0.00	-0.49	0.08	-0.70	inf	-0.30	-0.37
4	BnaA02g33340D	AT5G62290	nucleotide-sensitive chloride conductance regulator (ICln) family protein	-0.21	0.53	0.18	0.24	0.55	0.53	1.18	0.11	-0.80	0.94	-0.04	0.35	0.63	-0.17	0.27	-0.09
4	BnaA02g33350D	AT5G62310	INCOMPLETE ROOT HAIR ELONGATION (IRE)	0.00	0.00	0.00	0.00	0.00	0.00	0.00	0.00	0.00	0.00	0.00	0.00	0.00	0.00	0.00	0.00
4	BnaA02g33360D	AT5G62310	INCOMPLETE ROOT HAIR ELONGATION (IRE)	-0.48	-2.89	inf	0.00	-0.07	0.10	0.00	0.00	0.12	inf	0.49	0.11	0.00	1.25	-0.99	-0.52
4	BnaA02g33370D	AT5G62350	Plant invertase/pectin methylesterase inhibitor superfamily protein	0.28	0.48	0.12	-3.24	0.10	-0.54	-2.93	0.22	-0.13	0.76	-0.25	0.18	-0.96	0.37	-0.31	-0.44
4	BnaA02g33380D	AT5G62380	NAC-domain protein 101 (NAC101)	-0.04	0.98	0.02	inf	inf	0.00	0.00	-0.87	-1.36	-1.37	0.20	0.00	inf	0.00	-0.98	0.00
4	BnaA02g33390D	AT5G62420	NAD(P)-linked oxidoreductase superfamily protein	-0.57	-0.07	0.19	-0.25	inf	-0.23	-0.12	-0.18	-0.91	0.55	-0.16	0.27	0.26	1.05	1.98	2.33
4	BnaA02g33400D	AT3G10290	Nucleotide-sugar transporter family protein	0.00	0.00	0.00	0.00	0.00	0.00	0.00	0.00	0.00	0.00	0.00	0.00	0.00	0.00	0.00	0.00
4	BnaA02g33410D	AT5G62470	myb domain protein 96 (MYB96)	-3.59	-1.23	-0.49	-0.95	-0.60	0.85	0.09	0.17	1.02	-2.41	-0.45	-0.42	0.70	-0.71	-0.34	-1.10
4	BnaA02g33420D	AT5G62490	HVA22 homologue B (HVA22B)	0.00	2.09	0.44	1.11	-0.68	0.62	1.24	-0.03	0.00	0.00	inf	inf	inf	inf	-0.06	0.06
4	BnaA02g33430D	AT5G62500	end binding protein 1B (EB1B)	0.00	0.00	0.00	0.00	0.00	0.00	0.00	0.00	0.00	0.00	0.00	0.00	0.00	0.00	0.00	0.00
4	BnaA02g33440D	AT1G51250	Plant self-incompatibility protein S1 family	0.00	0.00	0.00	0.00	0.00	0.00	0.00	0.00	0.00	0.00	0.00	0.00	0.00	0.00	0.00	0.00
4	BnaA02g33450D	AT5G62520	similar to RCD one 5 (SRO5)	1.73	0.00	1.41	-1.32	-0.12	1.04	-0.89	0.93	1.44	-0.89	0.67	2.95	0.57	0.42	-0.28	-0.90
4	BnaA02g33460D	AT5G62530	aldehyde dehydrogenase 12A1 (ALDH12A1)	0.00	0.00	0.00	0.00	0.00	0.00	0.00	0.00	0.00	0.00	0.00	0.00	0.00	0.00	0.00	0.00

4	BnaA02g33470D	AT5G62530	aldehyde dehydrogenase 12A1 (ALDH12A1)	-1.81	0.00	0.00	0.62	0.10	0.24	0.90	0.00	-1.69	inf	inf	inf	-0.38	-1.74	0.32	0.01
4	BnaA02g33480D	AT5G62540	ubiquitin-conjugating enzyme 3 (UBC3)	0.39	0.75	0.53	0.69	0.03	0.20	0.71	0.16	-0.48	0.38	-0.09	-0.20	-0.63	-0.49	-0.63	-0.57
4	BnaA02g33490D	AT2G31050	Cupredoxin superfamily protein	0.00	0.00	0.00	inf	0.00	0.00	0.00	0.00	0.00	0.00	0.00	0.00	inf	0.00	0.00	0.00
4	BnaA02g33500D	AT5G62560	RING/U-box superfamily protein with ARM repeat domain	0.32	0.10	-0.35	-0.16	-0.18	-0.14	-0.32	-0.38	0.08	0.16	-0.27	0.43	0.13	0.29	0.58	0.53
4	BnaA02g33510D			0.00	0.00	0.00	inf	0.00	0.00	0.00	0.00	0.00	0.00	inf	0.00	0.00	0.00	0.79	0.00
4	BnaA02g33520D	AT5G62670	H(+)-ATPase 11 (HA11)	0.50	1.40	0.02	0.17	-0.19	0.27	-0.07	-0.29	0.33	-0.26	-0.26	0.23	2.14	0.47	0.25	0.04
4	BnaA02g33530D	AT5G62680	Major facilitator superfamily protein	-0.48	-0.56	0.23	0.27	-0.09	0.20	0.00	0.54	0.15	1.11	0.34	1.25	-0.11	-0.28	0.11	-0.13
4	BnaA02g33540D	AT5G62820	Uncharacterised protein family (UPF0497)	0.11	1.67	-0.24	-0.78	0.18	-1.60	-0.73	0.15	-0.20	1.28	-0.29	0.20	-0.27	-0.82	-0.17	-0.93
4	BnaA02g33550D	AT5G62850	VEGETATIVE CELL EXPRESSED1 (AtVEX1)	0.00	0.00	1.68	-0.74	inf	0.00	inf	0.89	-1.13	0.00	0.34	-0.69	inf	0.00	-0.16	-1.62
4	BnaA02g33560D	AT5G62850	VEGETATIVE CELL EXPRESSED1 (AtVEX1)	-0.48	0.00	0.23	0.00	0.00	inf	0.00	-5.02	inf	0.00	0.92	4.25	0.00	0.00	0.00	0.00
4	BnaA02g33570D	AT5G62060	F-box and associated interaction domains-containing protein	0.00	0.00	0.00	0.00	0.00	0.00	0.00	inf	0.00	0.00	0.00	0.00	0.00	0.00	0.00	0.00
4	BnaA02g33580D	AT5G62865	unknown protein	-0.32	-0.75	-0.21	-0.55	-0.35	0.53	-1.32	-0.26	-0.45	-1.04	0.54	1.91	0.56	0.73	0.98	0.78
4	BnaA02g33590D			0.00	0.00	0.00	0.00	0.00	0.00	0.00	0.00	0.00	0.00	0.00	0.00	0.00	0.00	0.00	0.00
4	BnaA02g33600D	AT5G62880	RAC-like 10 (RAC10)	-0.11	-0.35	0.13	0.07	-0.25	0.16	0.31	0.03	-0.23	1.36	-0.23	0.31	-0.25	0.35	0.65	-0.20
4	BnaA02g33610D	AT5G62890	Xanthine/uracil permease family protein	-0.06	0.56	-0.41	-1.25	0.35	-0.33	-1.51	-0.56	-0.01	0.54	-0.28	-0.36	1.38	-0.27	0.32	-0.28
4	BnaA02g33620D	AT5G62910	RING/U-box superfamily protein	0.41	0.11	0.79	-0.34	0.10	1.50	-0.60	0.19	0.52	0.45	0.06	0.74	0.00	-0.03	0.13	-0.68
4	BnaA02g33630D	AT5G62950	RNA polymerase II, Rpb4, core protein	0.21	-0.62	-0.24	0.38	-0.23	0.38	0.96	-0.04	0.97	0.64	0.25	0.11	-0.07	1.20	0.00	-0.13
4	BnaA02g33640D	AT3G62850	zinc finger protein-related	0.00	0.00	inf	0.00	-1.47	0.00	0.00	-1.31	0.00	0.00	-1.39	-1.48	0.00	0.00	0.00	0.00
4	BnaA02g33650D	AT5G63160	BTB and TAZ domain protein 1 (BT1)	0.93	0.23	0.00	1.20	-0.83	-0.54	inf	0.00	-0.09	0.76	-0.57	0.72	-0.76	-0.30	inf	inf
4	BnaA02g33660D	AT5G63170	GDSL-like Lipase/Acylhydrolase superfamily protein	0.00	0.00	0.00	0.00	0.00	0.00	0.00	0.00	0.00	0.00	0.00	0.00	0.00	0.00	0.00	0.00
4	BnaA02g33670D	AT5G63270	RPM1-interacting protein 4 (RIN4) family protein	0.00	0.00	0.00	0.00	0.00	0.00	0.00	0.00	0.00	0.00	0.00	0.00	0.00	0.00	0.00	0.00
4	BnaA02g33680D	AT5G63280	C2H2-like zinc finger protein	-0.15	-0.40	0.12	-0.51	0.28	0.19	-0.57	0.06	-0.10	0.30	-0.15	0.02	-0.46	-0.36	0.05	-0.27
4	BnaA02g33690D	AT5G63350	unknown protein	1.10	0.00	-0.12	3.79	0.84	inf	1.64	1.06	0.00	-1.06	0.32	0.97	1.24	0.00	-0.02	2.37
4	BnaA02g33700D	AT5G63460	SAP domain-containing protein	0.00	0.00	0.00	0.00	0.00	0.00	0.00	0.00	0.00	0.00	0.00	0.00	0.00	0.00	0.00	0.00
4	BnaA02g33710D	AT5G63490	CBS / octicosapeptide/Phox/Bemp1 (PB1) domains-containing protein	0.17	-0.70	0.29	0.47	0.28	0.52	0.57	0.06	0.60	0.16	0.11	0.15	-0.41	-0.42	-0.09	0.22
4	BnaA02g33720D	AT3G48680	gamma carbonic anhydrase-like 2 (GAMMA CAL2)	0.01	-0.06	-0.15	-0.65	-0.13	-0.40	-0.36	0.02	-0.20	0.10	-0.03	0.37	-0.34	0.55	0.09	0.59
4	BnaA02g33730D	AT2G14378	LOCATED IN: endomembrane system	0.00	0.00	0.00	0.00	0.00	0.00	0.00	0.00	0.00	0.00	inf	0.00	0.00	0.00	0.00	0.00
4	BnaA02g33740D	AT5G52975	Protein of unknown function (DUF1278)	0.00	0.00	0.00	0.00	0.00	0.00	0.00	0.00	0.00	0.00	0.00	0.00	0.00	0.00	0.00	0.00
4	BnaA02g33750D			0.00	0.00	0.00	0.00	0.00	0.00	0.00	0.00	0.00	0.00	0.00	0.00	0.00	0.00	0.00	0.00
4	BnaA02g33760D	AT5G63520	CONTAINS InterPro DOMAIN/s: F-box domain, Skp2-like (InterPro:IPR022364), FIST C domain (InterPro:IPR019494), FIST domain, N-terminal (InterPro:IPR013702)	-0.45	-0.62	0.04	-0.07	-0.41	-0.11	0.05	-0.40	-0.23	0.34	0.22	-0.24	0.34	-0.29	0.12	-0.24
4	BnaA02g33770D	AT5G63530	farnesylated protein 3 (FP3)	0.08	0.07	-0.16	-0.17	-0.82	-0.36	-0.28	-0.11	-0.34	0.49	0.03	-0.09	0.70	0.85	0.07	-0.01
4	BnaA02g33780D	AT5G63590	flavonol synthase 3 (FLS3)	-0.97	0.11	-2.03	1.09	-3.07	0.18	0.42	-2.07	0.10	1.32	-0.01	-0.34	3.76	0.82	-0.05	0.27
4	BnaA02g33790D	AT1G34150	Pseudouridine synthase family protein	0.00	0.00	0.00	0.00	0.00	0.00	0.00	0.00	0.00	0.00	0.00	0.00	0.00	0.00	0.00	0.00
4	BnaA02g33800D	AT5G63590	flavonol synthase 3 (FLS3)	2.68	-1.48	0.36	-0.68	-1.04	0.63	-2.68	0.94	-0.92	inf	-2.74	-0.09	0.00	inf	-0.30	-2.72
4	BnaA02g33810D	AT5G63610	cyclin-dependent kinase E	0.00	0.00	0.00	0.00	0.00	0.00	0.00	0.00	0.00	0.00	0.00	0.00	0.00	0.00	0.00	0.00
4	BnaA02g33820D	AT1G14800	Nucleic acid-binding, OB-fold-like protein	0.00	0.00	0.00	0.00	0.00	0.00	0.00	0.00	0.00	0.00	0.00	0.00	0.00	0.00	0.00	0.00
4	BnaA02g33830D	AT5G63640	ENTH/VHS/GAT family protein	-0.17	-0.27	-0.30	0.12	0.20	0.13	0.00	-0.43	0.78	-0.72	-0.02	0.05	-0.36	-0.45	0.31	-0.13
4	BnaA02g33840D			0.00	0.00	inf	0.00	0.00	0.00	0.00	0.00	0.00	0.00	0.00	0.00	0.00	0.00	0.00	0.00
4	BnaA02g33850D	AT5G63660	PDF2.5	0.00	0.00	0.00	0.00	0.00	0.00	0.00	0.00	0.00	0.00	0.00	0.00	0.00	0.00	inf	0.00
4	BnaA02g33860D	AT5G63670	SPT4 homolog 2 (SPT42)	-0.15	0.19	-0.27	-0.17	0.30	-0.32	0.10	-0.09	0.09	0.14	-0.01	-0.01	0.37	0.24	0.14	-0.15
4	BnaA02g33870D	AT5G63690	Nucleic acid-binding, OB-fold-like protein	-0.25	-1.09	0.30	1.38	0.67	-0.64	2.23	-3.86	0.00	1.79	0.64	0.47	0.08	0.82	0.22	1.44
4	BnaA02g33880D	AT5G63750	ARIADNE 13 (ARI13)	-1.93	-2.73	1.18	-0.22	-0.57	-0.05	-0.36	0.45	-0.10	-3.19	0.40	0.65	0.48	0.01	0.34	0.26
4	BnaA02g33890D	AT5G63770	diacylglycerol kinase 2 (DGK2)	0.00	0.00	0.00	0.00	0.00	0.00	0.00	0.00	0.00	0.00	0.00	0.00	0.00	0.00	0.00	0.00
4	BnaA02g33900D	AT5G63780	shoot apical meristem arrest 1 (SHA1)	0.12	0.65	0.06	-0.44	0.00	-0.39	-0.60	0.00	0.44	0.09	-0.16	0.55	-0.24	0.23	-0.45	-0.20
4	BnaA02g33910D	AT5G63790	NAC domain containing protein 102 (NAC102)	-0.35	-0.09	0.07	-0.49	0.07	0.51	0.52	-0.22	1.12	-0.60	0.14	0.26	-0.31	0.34	-0.07	0.96

4	BnaA02g33920D	AT5G63800	MUCILAGE-MODIFIED 2 (MUM2)	1.08	-0.56	-0.75	0.23	-0.57	0.12	-0.01	-0.76	0.15	1.27	0.53	1.14	0.48	-1.22	0.09	-0.27
4	BnaA02g33930D	AT5G63850	amino acid permease 4 (AAP4)	-3.35	0.00	-3.58	0.00	0.00	0.00	inf	-3.23	0.00	inf	1.56	2.40	2.31	1.69	0.63	0.00
4	BnaA02g33940D	AT5G63860	UVB-RESISTANCE 8 (UVR8)	0.54	-0.11	0.36	-0.49	-0.08	-0.06	-0.46	0.27	0.12	-0.24	-0.04	0.33	-0.35	-0.04	0.07	-0.73
4	BnaA02g33950D			0.00	0.00	0.00	0.00	0.00	0.00	0.00	0.00	0.00	0.00	0.00	0.00	0.00	0.00	0.00	0.00
4	BnaA02g33960D	AT5G63920	topoisomerase 3alpha (TOP3A)	-0.04	1.77	0.37	-0.69	0.94	-0.20	-1.66	0.13	-0.80	0.59	0.20	0.26	-0.02	1.47	0.29	0.60
4	BnaA02g33970D	AT5G63940	Protein kinase protein with adenine nucleotide alpha hydrolases-like domain	-0.46	-0.60	-0.23	0.29	-0.14	-0.19	-0.02	-0.34	0.18	0.33	0.12	0.00	0.62	0.40	0.76	0.25
4	BnaA02g33980D	AT5G63970	Copine (Calcium-dependent phospholipid-binding protein) family	0.77	0.71	-0.79	0.36	-0.22	-0.13	-0.08	-1.00	0.02	1.46	-0.07	0.55	0.84	-1.52	0.01	-0.20
4	BnaA02g33990D	AT4G24260	glycosyl hydrolase 9A3 (GH9A3)	0.00	0.00	0.00	0.00	0.00	0.00	0.00	0.00	0.00	0.00	0.00	0.00	0.00	0.00	0.00	0.00
4	BnaA02g34000D	AT5G64000	SAL2	-0.75	-0.53	-0.09	0.91	1.61	0.48	0.67	-0.06	0.46	0.34	-0.07	-0.08	0.76	-0.51	0.70	-0.46
4	BnaA02g34010D	AT5G64020	TRICHOME BIREFRINGENCE-LIKE 14 (TBL14)	0.40	-0.31	0.15	0.08	0.71	0.44	-0.71	-0.08	0.03	0.18	-0.39	0.97	-0.37	0.24	1.51	-0.32
4	BnaA02g34020D	AT5G64030	S-adenosyl-L-methionine-dependent methyltransferases superfamily protein	0.02	-0.10	-0.07	-0.62	0.43	-0.15	-0.49	-0.10	-0.16	0.20	-0.11	0.14	0.16	0.39	0.12	-0.03
4	BnaA02g34030D	AT5G64040	PSAN	-1.56	-1.21	-0.72	-4.23	-0.25	-0.48	-3.64	-1.94	0.12	1.27	-2.28	-0.16	0.43	-0.41	-0.12	-0.26
4	BnaA02g34040D	AT5G64070	phosphatidylinositol 4-OH kinase beta1 (PI-4KBETA1)	0.10	-0.87	-0.18	-0.03	-0.20	-0.20	-0.22	-0.51	0.19	-0.32	-0.05	0.03	-0.02	-0.08	-0.02	0.25
4	BnaA02g34050D	AT5G64080	Bifunctional inhibitor/lipid-transfer protein/seed storage 2S albumin superfamily protein	0.61	0.65	-0.34	-0.25	-0.30	0.79	-0.87	-0.21	-0.40	0.20	-0.62	0.55	3.44	-0.43	0.30	-0.01
4	BnaA02g34060D	AT5G64100	Peroxidase superfamily protein	0.00	0.00	0.00	0.00	0.00	0.00	inf	0.00	0.00	0.00	0.00	0.00	0.00	0.00	0.00	0.00
4	BnaA02g34070D	AT5G64120	Peroxidase superfamily protein	-0.58	-1.06	0.65	-0.70	-3.54	-0.78	0.00	-2.13	-1.45	2.16	inf	inf	1.31	1.53	0.00	0.00
4	BnaA02g34080D	AT5G64130	cAMP-regulated phosphoprotein 19-related protein	0.20	0.81	0.11	-0.37	0.14	0.24	-0.34	-0.21	-0.68	-0.23	-0.23	0.06	0.14	0.25	0.30	-0.48
4	BnaA02g34090D	AT5G64230	unknown protein	-0.38	-0.66	-0.41	0.03	0.00	0.92	-0.26	-0.35	0.45	-0.15	0.45	0.24	0.75	1.02	0.35	0.36
4	BnaA02g34100D	AT5G41770	crooked neck protein, putative / cell cycle protein, putative	2.72	0.00	0.97	0.00	1.43	inf	0.00	1.95	-0.98	0.00	-1.84	-0.89	0.33	0.00	0.00	inf
4	BnaA02g34110D	AT5G64240	metacaspase 3 (MC3)	-0.27	-0.12	-0.64	inf	-0.26	0.42	-0.15	-0.66	0.34	-0.42	-0.56	0.45	-0.03	0.92	1.86	-0.38
4	BnaA02g34120D	AT5G64260	EXORDIUM like 2 (EXL2)	-0.13	-1.66	-0.80	0.09	-1.03	-0.32	-0.27	-1.24	-0.77	0.29	1.09	1.05	1.52	1.00	0.87	1.26
4	BnaA02g34130D	AT5G64280	dicarboxylate transporter 2.2 (DiT2.2)	-0.18	-0.12	-0.35	0.07	0.36	-0.14	-0.13	-0.33	0.43	0.31	0.10	0.56	0.38	0.21	0.16	0.15
4	BnaA02g34140D	AT5G64310	arabinogalactan protein 1 (AGP1)	0.17	1.37	-0.06	-2.53	-0.07	0.95	-2.66	0.15	-0.74	0.08	-0.35	0.30	0.07	0.15	-0.85	1.48
4	BnaA02g34150D	AT5G64320	Pentatricopeptide repeat (PPR) superfamily protein	-0.14	0.12	0.09	0.10	0.29	-0.09	0.12	-0.09	-0.42	0.14	0.03	0.05	-0.05	0.34	0.22	0.25
4	BnaA02g34160D	AT5G64340	SUPPRESSOR OF ACAULIS 51 (SAC51)	0.19	-0.31	-0.01	-0.42	-0.36	-0.85	-0.74	-0.56	0.35	-0.19	0.05	-0.66	-0.16	-0.49	-0.24	-0.42
4	BnaA02g34170D	AT5G64341	conserved peptide upstream open reading frame 40 (CPuORF40)	0.25	0.00	inf	0.00	0.00	0.00	0.00	0.00	-0.87	0.00	0.00	0.00	0.00	0.31	0.00	0.00
4	BnaA02g34180D	AT5G64350	FK506-binding protein 12 (FKBP12)	0.00	0.00	0.00	0.00	0.00	0.00	0.00	0.00	0.00	0.00	0.00	0.00	0.00	0.00	0.00	0.00
4	BnaA02g34190D	AT5G64360	Chaperone DnaJ-domain superfamily protein	-0.19	-0.22	-0.25	-0.33	-0.03	0.05	-0.87	0.09	-0.65	0.59	0.28	0.37	0.57	0.61	0.50	0.55
4	BnaA02g34200D	AT5G64410	oligopeptide transporter 4 (OPT4)	-0.23	-0.69	-0.20	-0.43	0.17	-0.52	-0.51	-0.32	0.95	-0.15	-0.38	-0.35	-0.53	0.36	0.02	-0.08
4	BnaA02g34210D	AT5G64430	Octicosapeptide/Phox/Bem1p family protein	0.09	-0.44	0.07	0.27	-1.16	0.88	-0.07	0.17	-0.41	1.16	0.40	0.09	0.41	0.17	0.28	-0.22
4	BnaA02g34220D	AT5G64430	Octicosapeptide/Phox/Bem1p family protein	-0.32	-1.32	-0.33	0.10	-1.97	1.23	1.21	-0.16	0.29	-0.31	0.22	0.33	0.51	-0.75	0.46	0.18
4	BnaA02g34230D	AT5G64440	fatty acid amide hydrolase (FAAH)	-0.80	0.96	-0.10	-0.87	0.00	-0.38	-0.76	0.03	-0.03	inf	0.18	-0.17	0.00	0.00	0.74	-0.94
4	BnaA02g34240D	AT5G61720	Protein of unknown function (DUF1216)	0.00	0.00	-7.73	0.00	0.00	0.00	0.00	-6.68	inf	0.00	2.71	3.21	0.00	0.00	inf	0.00
4	BnaA02g34250D	AT5G64530	xylem NAC domain 1 (XND1)	0.00	inf	0.00	0.00	0.00	0.00	0.00	0.83	-1.42	0.00	0.20	0.00	1.22	2.87	-0.26	-1.39
4	BnaA02g34260D	AT5G64550	loricrin-related	-2.79	-1.14	-2.51	-1.00	0.91	0.36	-1.27	-2.27	1.03	-0.21	-0.07	-0.23	0.49	0.55	0.89	1.11
4	BnaA02g34270D	AT5G64560	magnesium transporter 9 (MGT9)	0.00	0.00	0.25	-0.33	0.00	0.91	-1.63	0.19	0.00	0.00	0.34	0.06	0.00	0.00	inf	inf
4	BnaA02g34280D	AT5G64570	beta-D-xylosidase 4 (XYL4)	1.05	1.49	-0.25	-1.52	-0.20	-0.80	-2.56	-0.19	-0.88	0.00	-0.11	0.42	-0.35	0.68	1.03	-0.24
4	BnaA02g34290D			0.00	0.00	0.00	0.00	0.00	0.00	0.00	0.00	0.00	0.00	0.00	0.00	0.00	0.00	0.00	0.00
4	BnaA02g34300D	AT2G02610	Cysteine/Histidine-rich C1 domain family protein	-0.01	0.75	-0.68	0.79	0.06	-0.92	0.44	-0.26	-0.22	-0.20	-0.72	0.29	1.10	inf	-0.31	-0.48
4	BnaA02g34310D	AT5G64680	unknown protein	-0.30	-0.82	0.39	0.49	-0.16	-0.42	0.82	-0.07	-0.12	0.04	-0.01	0.00	0.39	0.20	-0.03	0.29
4	BnaA02g34320D	AT5G64690	neurofilament triplet H protein-related	-1.40	0.00	0.00	0.00	0.00	0.00	0.00	-2.13	0.00	inf	1.05	inf	0.00	0.00	0.00	0.00
4	BnaA02g34330D	AT5G64710	Putative endonuclease or glycosyl hydrolase	0.14	-0.70	0.36	0.84	0.16	0.17	-0.33	0.18	0.13	-0.19	-0.21	0.23	-0.84	-0.94	0.15	0.63
4	BnaA02g34340D	AT4G19590	Chaperone DnaJ-domain superfamily protein	0.00	0.00	0.00	0.00	0.00	0.00	0.00	0.00	0.00	0.00	-0.34	0.23	0.00	0.00	0.00	-1.43
4	BnaA02g34350D	AT5G65260	RNA-binding (RRM/RBD/RNP motifs) family protein	0.15	0.23	-0.07	-0.60	0.34	0.16	-0.63	-0.12	0.00	-0.59	-0.16	0.31	-0.77	0.22	0.27	-0.53
4	BnaA02g34360D	AT5G64740	cellulose synthase 6 (CESA6)	0.47	0.08	-0.34	-0.44	-0.30	-0.13	-0.64	-0.29	0.19	-0.01	-0.17	0.09	-0.23	0.85	-0.08	-0.30
4	BnaA02g34370D	AT5G64750	ABA REPRESSOR1 (ABR1)	0.10	0.00	2.63	2.90	1.04	1.97	2.80	0.00	inf	0.98	0.00	0.39	1.76	-0.19	0.91	-1.04

5	BnaA03g27360D	AT3G01670	unknown protein	0.38	1.00	-0.48	1.59	-1.71	-1.67	0.28	-0.49	-0.19	0.30	-0.12	0.47	-0.18	-0.08	0.11	0.61
5	BnaA03g27370D	AT3G01650	RING domain ligase1 (RGLG1)	-0.17	-0.99	-0.38	1.19	-0.17	0.36	1.02	-0.09	-0.06	-0.06	0.13	0.75	0.78	0.07	-0.21	-0.22
5	BnaA03g27380D	AT3G01640	glucuronokinase G (GLCAK)	0.06	0.05	-0.29	-0.21	-0.05	-0.06	-0.17	-0.08	-0.34	-0.13	0.25	0.47	0.18	0.13	-0.20	0.10
5	BnaA03g27390D	AT3G01610	cell division cycle 48C (CDC48C)	0.11	0.38	0.15	0.29	0.07	-0.12	0.40	0.27	-0.18	-0.25	-0.19	0.07	-0.12	-0.19	-0.59	-0.20
5	BnaA03g27400D	AT3G01590	Galactose mutarotase-like superfamily protein	-0.41	-0.63	-0.24	0.43	-0.28	0.20	0.54	-0.10	0.40	0.16	0.37	-0.23	0.19	-0.67	0.22	-0.42
5	BnaA03g27410D	AT3G01580	Tetratricopeptide repeat (TPR)-like superfamily protein	-4.41	0.25	-0.16	0.72	-1.39	-0.14	0.37	-0.19	-4.19	-1.43	3.62	4.68	8.49	-0.78	0.15	0.17
5	BnaA03g27420D	AT3G01550	phosphoenolpyruvate (pep)/phosphate translocator 2 (PPT2)	0.54	0.90	0.46	1.82	-0.07	0.57	2.30	0.35	1.23	0.76	0.19	0.40	-0.14	-0.06	-0.47	0.73
5	BnaA03g27430D	AT5G14610	DEAD box RNA helicase family protein	-0.27	0.05	-0.16	0.06	0.49	0.22	-0.26	-0.25	0.03	0.20	-0.24	0.11	0.64	0.35	0.11	-0.19
5	BnaA03g27440D	AT3G01530	myb domain protein 57 (MYB57)	0.41	-0.64	-1.84	-0.09	-0.32	-0.86	0.00	-1.68	0.33	0.56	0.77	-0.45	-1.07	-0.09	0.32	-1.50
5	BnaA03g27450D	AT3G01510	like SEX4 1 (LSF1)	0.18	0.84	0.07	0.37	0.32	-0.49	0.53	-0.54	0.45	-0.01	0.15	0.35	0.13	0.43	-0.02	0.34
5	BnaA03g27460D	AT3G01410	Polynucleotidyl transferase, ribonuclease H-like superfamily protein	-0.19	2.23	0.13	-0.60	0.45	-0.59	-0.10	0.26	-1.32	-1.25	-0.48	-0.40	-1.05	-1.02	-0.27	-1.01
5	BnaA03g27470D	AT3G01510	like SEX4 1 (LSF1)	0.00	0.00	inf	0.00	0.00	0.00	0.00	0.00	inf	-0.11	0.00	0.00	0.00	0.00	0.00	0.00
5	BnaA03g27480D	AT3G01410	Polynucleotidyl transferase, ribonuclease H-like superfamily protein	0.00	inf	1.60	-0.97	-0.22	0.83	-2.36	1.59	0.83	1.05	-0.26	0.16	2.28	2.01	-1.49	0.47
5	BnaA03g27490D	AT3G01400	ARM repeat superfamily protein	-0.03	-0.26	-0.33	-0.24	-0.20	-0.05	-0.53	0.07	0.14	0.19	-0.16	1.01	0.05	0.18	0.57	0.35
5	BnaA03g27500D	AT3G01390	vacuolar membrane ATPase 10 (VMA10)	0.64	-0.85	0.81	0.78	1.43	0.84	0.86	0.37	-0.41	1.38	-0.06	3.38	2.46	1.43	1.23	0.97
5	BnaA03g27510D	AT3G01310	Phosphoglycerate mutase-like family protein	0.16	-1.53	-0.14	inf	3.36	0.74	inf	inf	-0.54	0.33	2.11	1.78	2.18	-0.11	1.07	-2.07
5	BnaA03g27520D	AT3G01290	SPFH/Band 7/PHB domain-containing membrane-associated protein family	-2.50	-0.58	-0.68	-1.12	-0.17	-0.21	-1.53	-0.26	1.04	0.97	0.71	2.24	0.53	-0.84	1.28	-1.85
5	BnaA03g27530D	AT3G01280	voltage dependent anion channel 1 (VDAC1)	0.28	0.18	0.18	0.23	0.16	0.08	0.46	0.28	-0.20	0.23	-0.10	0.30	0.28	-0.01	-0.09	-0.12
5	BnaA03g27540D	AT3G01270	Pectate lyase 7	-1.87	-1.51	-1.84	0.00	0.76	inf	0.00	-4.68	-0.79	0.75	1.37	8.53	0.00	0.00	inf	inf
5	BnaA03g27550D	AT5G15140	Galactose mutarotase-like superfamily protein	0.00	inf	0.00	0.00	0.00	0.00	0.00	inf	0.00	0.00	-1.12	0.00	0.00	0.00	0.00	0.00
5	BnaA03g27560D	AT3G01240	unknown protein	-0.45	-1.35	0.07	0.00	inf	0.00	0.00	-3.28	inf	0.00	1.03	inf	0.00	0.00	0.00	inf
5	BnaA03g27570D	AT4G15755	Calcium-dependent lipid-binding (CaLB domain) family protein	0.00	0.00	inf	0.00	0.00	0.00	0.00	0.00	0.00	0.00	inf	-0.38	0.00	0.00	0.00	0.00
5	BnaA03g27580D	AT3G01230	unknown protein	inf	-1.25	-0.42	0.00	0.00	0.00	0.00	-2.41	inf	0.00	1.57	inf	0.00	0.00	0.00	0.00
5	BnaA03g27590D	AT3G01220	homeobox protein 20 (HB20)	0.00	0.00	0.00	0.00	0.00	0.00	0.00	0.00	0.00	0.00	0.00	0.00	0.00	0.00	0.00	0.00
5	BnaA03g27600D	AT3G01210	RNA-binding (RRM/RBD/RNP motifs) family protein	0.37	0.43	0.45	0.21	-0.32	-0.59	0.60	0.52	0.18	0.33	-0.02	0.18	0.16	-0.65	0.07	0.04
5	BnaA03g27610D			0.00	0.00	0.00	0.00	0.00	0.00	0.00	0.00	0.00	0.00	0.00	0.00	0.00	0.00	0.00	0.00
5	BnaA03g27620D	AT3G01120	METHIONINE OVERACCUMULATION 1 (MTO1)	0.35	0.70	0.00	0.36	0.03	0.01	0.27	0.12	-0.03	0.14	-0.13	0.16	0.13	-0.03	-0.12	0.04
5	BnaA03g27630D	AT3G01100	hypothetical protein 1 (HYP1)	0.44	0.36	0.26	0.90	0.12	0.03	0.14	-0.02	0.25	0.13	-0.48	0.42	0.57	-0.25	-0.14	-0.01
5	BnaA03g27640D	AT3G01090	SNF1 kinase homolog 10 (KIN10)	0.20	0.04	0.13	0.61	0.26	-0.19	-0.16	0.25	0.01	0.18	-0.17	-0.22	0.93	-0.28	0.07	-0.35
5	BnaA03g27650D	AT3G01085	Protein kinase superfamily protein	0.17	0.06	-2.15	0.00	inf	0.00	0.00	-1.20	inf	inf	1.49	3.11	0.00	0.00	0.00	0.00
6	BnaA03g43820D	AT4G18960	AGAMOUS (AG)	0.00	0.44	-0.99	-2.14	0.31	-0.19	-2.34	-0.45	0.00	2.48	0.37	0.19	-0.21	0.59	0.89	-0.29
6	BnaA03g43830D	AT4G18970	GDSL-like Lipase/Acylhydrolase superfamily protein	0.10	inf	0.15	0.00	-2.19	-0.41	-3.00	0.39	-2.21	-0.66	-0.07	0.06	-0.97	2.33	1.43	-2.40
6	BnaA03g43840D			-0.98	-2.84	-0.23	-3.55	-2.00	-1.31	-1.66	0.76	-0.22	-0.11	-0.16	0.40	0.61	0.98	-0.24	0.86
6	BnaA03g43850D			0.00	-4.59	0.00	inf	0.00	0.00	inf	0.00	0.00	1.27	0.00	3.27	inf	2.33	0.49	0.55
6	BnaA03g43860D	AT4G18980	AtS40-3 (AtS40-3)	inf	0.00	-0.65	0.00	2.44	2.11	-1.04	0.40	0.51	0.21	-0.21	-0.74	0.05	0.51	inf	inf
6	BnaA03g43870D	AT4G18990	xyloglucan endotransglucosylase/hydrolase 29 (XTH29)	0.00	-1.15	-1.15	0.00	0.00	-0.03	0.00	-0.97	0.00	0.00	0.60	0.17	0.98	-0.14	-0.85	0.00
6	BnaA03g43880D	AT4G19040	ENHANCED DISEASE RESISTANCE 2 (EDR2)	-0.29	-1.05	-0.16	-0.28	0.04	0.12	-0.95	-0.57	-0.44	0.15	0.00	0.31	-0.07	0.10	-0.15	-0.53
6	BnaA03g43890D	AT4G19070	Putative membrane lipoprotein	-0.52	-0.02	-0.17	-0.42	0.30	0.83	-1.35	1.02	-0.67	-0.37	0.56	0.13	-0.56	0.31	-0.12	-0.94
6	BnaA03g43900D	AT4G19110	Protein kinase superfamily protein	0.04	-0.23	-0.33	0.04	0.01	0.00	-0.69	-0.34	-0.28	-0.07	-0.28	0.27	0.38	0.09	0.24	0.04
6	BnaA03g43910D	AT4G19120	early-responsive to dehydration 3 (ERD3)	0.69	-0.30	-0.26	-0.12	-0.32	-0.68	-0.05	-0.25	0.23	0.01	-0.25	0.16	-1.29	0.58	1.79	-0.01
6	BnaA03g43920D			0.00	0.00	0.00	0.00	0.00	0.00	0.00	0.00	0.00	0.00	0.00	0.00	0.00	0.00	0.00	0.00
6	BnaA03g43930D	AT4G19130	Replication factor-A protein 1-related	-0.07	0.10	0.06	-0.61	-0.25	-0.34	-0.40	-0.27	-0.08	-0.44	-0.10	-0.05	0.30	-0.33	-0.25	-0.19
6	BnaA03g43940D	AT4G19210	RNAse l inhibitor protein 2 (RLI2)	-0.38	-0.34	0.01	0.41	0.03	-0.11	0.74	-0.16	-0.03	-0.30	0.14	0.28	0.21	-0.21	-0.11	0.00
6	BnaA03g43950D	AT4G19220	Tetratricopeptide repeat (TPR)-like superfamily protein	0.00	-0.33	1.30	0.05	-0.13	-0.99	2.34	-0.03	1.03	-0.26	-0.45	-0.55	-2.72	-1.97	-1.33	1.14
6	BnaA03g43960D	AT4G19230	cytochrome P450, family 707, subfamily A, polypeptide 1 (CYP707A1)	-0.04	0.12	-0.28	0.32	0.30	0.41	0.06	-0.08	-0.36	-0.71	-0.21	0.20	-0.14	0.16	-0.27	0.78

6	BnaA03g43970D			0.00	0.00	0.00	0.00	0.00	0.00	0.00	0.00	0.00	0.00	0.00	0.00	0.00	0.00	0.00	0.00
6	BnaA03g43980D	AT4G19360	SCD6 protein-related	0.00	0.00	inf	0.00	0.00	0.00	inf	0.55	0.00	0.00	0.64	-0.68	inf	0.00	0.00	0.00
6	BnaA03g43990D	AT4G19380	Long-chain fatty alcohol dehydrogenase family protein	0.00	0.00	-0.41	-2.63	0.00	0.00	-0.05	0.23	0.00	0.00	1.09	-0.43	inf	0.00	0.14	-0.97
6	BnaA03g44000D	AT4G19380	Long-chain fatty alcohol dehydrogenase family protein	1.09	inf	0.23	-0.72	-0.65	-1.10	-0.31	-0.06	0.00	0.00	0.44	0.00	inf	-0.14	-0.05	-0.09
6	BnaA03g44010D	AT4G19430	unknown protein	0.00	0.00	-0.73	inf	0.00	-0.98	2.73	0.45	0.74	0.00	0.85	0.71	0.05	-0.33	inf	0.00
6	BnaA03g44020D	AT4G19440	Tetratricopeptide repeat (TPR)-like superfamily protein	0.00	0.00	0.00	0.00	0.00	inf	0.00	0.00	0.00	0.00	0.00	0.00	0.00	0.00	0.00	0.00
6	BnaA03g44030D			0.00	0.00	0.00	0.00	0.00	0.00	0.00	0.00	0.00	0.00	inf	0.00	0.00	0.00	0.00	0.00
6	BnaA03g44040D	AT4G23230	cysteine-rich RLK (RECEPTOR-like protein kinase) 15 (CRK15)	0.30	-0.87	0.12	0.00	1.98	0.60	0.00	0.19	1.16	-0.23	0.95	-0.71	-0.88	-0.53	-1.17	0.00
6	BnaA03g44050D	AT4G19450	Major facilitator superfamily protein	-0.54	-0.25	0.00	0.44	0.29	0.03	-1.25	-0.23	0.21	0.60	-0.14	0.02	0.61	-0.61	1.95	-0.89
6	BnaA03g44060D	AT4G19460	UDP-Glycosyltransferase superfamily protein	-0.92	0.00	-0.41	0.00	0.12	0.96	0.00	0.00	-2.24	0.00	0.00	inf	0.20	2.34	-1.94	0.00
6	BnaA03g44070D	AT4G19470	Leucine-rich repeat (LRR) family protein	0.17	-0.90	-0.36	0.66	-0.56	-0.12	-0.93	0.02	-0.28	0.96	0.51	-0.16	-0.72	-0.95	0.08	0.18
6	BnaA03g44080D	AT4G12010	Disease resistance protein (TIR-NBS-LRR class) family	0.02	-1.89	-0.27	-0.68	-0.87	-0.79	-0.76	0.08	0.38	0.27	0.01	-0.58	-0.81	-0.27	-0.11	0.54
6	BnaA03g44090D	AT4G19500	nucleoside-triphosphatases	inf	inf	inf	0.00	inf	inf	0.00	inf	0.00	0.00	-0.37	inf	inf	0.00	2.79	0.00
6	BnaA03g44100D	AT4G19490	VPS54	0.14	-0.12	-0.02	0.13	0.11	0.27	-0.28	-0.14	0.02	0.09	-0.08	-0.07	0.08	0.19	-0.08	-0.11
6	BnaA03g44110D	AT4G19520	disease resistance protein (TIR-NBS-LRR class) family	0.00	0.00	0.00	0.00	0.00	0.00	0.00	0.00	0.00	0.00	0.00	0.00	0.00	0.00	0.00	0.00
6	BnaA03g44120D	AT4G35120	Galactose oxidase/kelch repeat superfamily protein	0.00	0.00	0.00	0.00	0.00	0.00	0.00	0.00	0.00	0.00	0.00	0.00	0.00	0.00	0.00	0.00
6	BnaA03g44130D	AT4G19930	F-box and associated interaction domains-containing protein	0.00	0.00	inf	0.00	0.00	0.00	0.00	0.07	0.00	0.00	2.01	0.73	0.00	0.00	0.00	0.00
6	BnaA03g44140D	AT4G19670	RING/U-box superfamily protein	-0.29	3.28	0.34	0.93	-0.38	1.39	-2.11	inf	0.36	0.00	inf	0.62	3.40	inf	0.00	0.70
6	BnaA03g44150D	AT4G19680	iron regulated transporter 2 (IRT2)	0.00	0.00	0.00	0.00	0.00	0.00	0.00	0.00	0.00	0.00	0.00	0.00	0.00	0.00	inf	0.00
6	BnaA03g44160D	AT4G19690	iron-regulated transporter 1 (IRT1)	0.00	0.00	0.00	0.00	0.00	0.00	0.00	0.00	0.00	0.00	0.00	0.00	0.00	0.00	0.00	0.00
6	BnaA03g44170D	AT4G19690	iron-regulated transporter 1 (IRT1)	0.00	0.00	0.00	0.00	0.00	0.00	0.00	0.00	0.00	0.00	0.00	0.00	0.00	0.00	0.00	0.00
6	BnaA03g44180D	AT4G19690	iron-regulated transporter 1 (IRT1)	0.00	0.00	0.00	inf	0.00	0.00	0.00	0.00	0.00	0.00	0.61	0.00	0.00	0.00	inf	0.00
6	BnaA03g44190D	AT4G19860	alpha/beta-Hydrolases superfamily protein	0.73	-0.42	0.10	-0.25	0.27	0.35	-0.61	-0.23	0.62	0.54	0.03	0.48	0.42	-0.24	0.16	-0.16
6	BnaA03g44200D	AT3G20180	Copper transport protein family	0.00	0.00	0.00	0.00	0.00	0.00	0.00	0.00	0.00	0.00	0.00	0.00	0.00	0.00	0.00	0.00
6	BnaA03g44210D	AT5G57990	ubiquitin-specific protease 23 (UBP23)	0.00	0.00	0.00	0.00	0.00	0.00	0.00	0.00	0.00	0.00	0.00	0.00	0.00	0.00	0.00	0.00
6	BnaA03g44220D			0.00	0.00	0.00	0.00	0.00	0.00	0.00	0.00	0.00	0.00	0.00	0.00	0.00	0.00	0.00	inf
6	BnaA03g44230D	AT3G15710	Peptidase S24/S26A/S26B/S26C family protein	0.00	0.00	0.00	0.00	0.00	0.00	0.00	0.00	0.00	0.00	0.00	0.00	0.00	0.00	0.00	0.00
6	BnaA03g44240D	AT3G54070	Ankyrin repeat family protein	0.00	0.00	0.00	0.00	0.00	0.00	0.00	0.00	0.00	0.00	2.12	0.00	0.00	0.00	0.00	0.00
6	BnaA03g44250D			0.00	0.00	0.00	0.00	0.00	0.00	0.00	0.00	0.00	0.00	0.00	0.00	0.00	0.00	0.00	0.00
6	BnaA03g44260D	AT2G07760	Zinc knuckle (CCHC-type) family protein	0.00	0.00	0.00	0.00	0.00	0.00	0.00	0.00	0.00	0.00	0.00	0.00	0.00	0.00	0.00	0.00
6	BnaA03g44270D	AT4G19860	alpha/beta-Hydrolases superfamily protein	-1.58	-0.61	-0.95	0.30	-1.47	-1.07	-1.00	0.56	2.04	-1.83	0.73	1.04	0.30	-0.61	0.45	-0.21
6	BnaA03g44280D	AT4G19880	Glutathione S-transferase family protein	-0.43	-0.07	-0.50	1.01	0.23	0.23	1.59	-0.01	0.10	-0.36	0.66	-0.37	0.13	-0.23	0.01	-0.26
6	BnaA03g44290D			0.00	0.00	0.00	0.00	0.00	0.00	0.00	0.00	0.00	0.00	0.00	0.00	0.00	0.00	0.00	0.00
6	BnaA03g44300D	AT4G19940	F-box and associated interaction domains-containing protein	0.00	0.00	inf	0.00	0.00	0.00	0.00	0.00	0.00	0.00	0.00	0.00	0.00	0.00	0.00	0.00
6	BnaA03g44310D	AT4G19880	Glutathione S-transferase family protein	0.00	0.00	0.00	0.00	0.00	0.00	0.00	0.00	0.00	0.00	0.00	0.00	0.00	0.00	0.00	0.00
6	BnaA03g44320D	AT4G19960	K+ uptake permease 9 (KUP9)	-1.01	-0.90	0.00	2.51	0.18	-1.58	-0.10	0.00	inf	0.00	0.00	0.00	inf	0.39	-0.92	0.77
6	BnaA03g44330D	AT4G19960	K+ uptake permease 9 (KUP9)	0.00	0.00	0.00	0.00	0.00	0.00	0.00	-0.31	0.00	inf	0.00	-0.39	0.00	0.00	0.00	0.00
6	BnaA03g44340D	AT4G19970	CONTAINS InterPro DOMAIN/s: Nucleotide-diphospho-sugar transferase, predicted (InterPro:IPR005069)	0.11	0.10	-0.12	-0.08	-0.15	0.72	-0.35	0.22	0.01	1.48	-0.61	0.18	-0.19	-0.33	-0.67	0.27
6	BnaA03g44350D	AT4G20010	plastid transcriptionally active 9 (PTAC9)	-0.41	-0.13	0.11	-0.27	0.11	-0.22	-0.18	-0.04	0.52	-0.31	-0.24	0.06	-0.52	-0.19	0.43	-0.22
6	BnaA03g44360D	AT4G20020	unknown protein	-0.23	-1.70	0.02	0.96	0.92	-0.08	1.54	-0.46	-1.10	-0.21	-0.25	-0.35	-1.05	0.73	0.31	1.15
6	BnaA03g44370D	AT4G20030	RNA-binding (RRM/RBD/RNP motifs) family protein	0.00	1.69	0.00	0.00	0.00	0.47	inf	0.00	inf	0.86	inf	2.30	0.49	-1.04	0.00	0.00
6	BnaA03g44380D	AT4G20040	Pectin lyase-like superfamily protein	1.19	0.00	-1.08	0.06	-0.45	-2.31	-0.05	0.60	-1.78	2.50	0.84	0.45	1.36	0.00	0.65	-0.31
6	BnaA03g44390D			0.00	0.00	0.00	0.00	0.00	0.00	0.00	0.00	0.00	0.00	0.00	0.00	0.00	0.00	0.00	0.00
6	BnaA03g44400D	AT4G20070	allantoate amidohydrolase (AAH)	0.56	-0.37	-0.61	0.05	-0.13	-0.17	0.03	-0.30	-0.21	0.20	0.03	-0.05	0.43	-0.61	-0.19	-0.33
6	BnaA03g44410D	AT2G30770	cytochrome P450, family 71, subfamily A, polypeptide 13 (CYP71A13)	0.00	0.00	0.00	0.59	0.00	-2.59	1.67	inf	0.00	0.00	0.95	inf	2.61	-1.64	-0.86	-3.08
6	BnaA03g44420D	AT4G20150	unknown protein	-0.76	-0.43	-0.13	-0.79	-0.77	-0.35	-0.61	-0.40	-0.95	0.27	-0.02	-0.07	-0.20	0.20	-0.43	0.41
6	BnaA03g44430D			0.00	0.00	0.00	2.98	inf	inf	1.17	0.00	0.00	0.00	0.00	inf	inf	0.00	1.88	0.44

6	BnaA03g44440D	AT4G20200	Terpenoid cyclases/Protein prenyltransferases superfamily protein	0.00	inf	-0.65	0.24	-2.59	0.99	0.22	0.29	0.00	0.00	inf	4.20	8.57	-1.31	-0.60	0.06
6	BnaA03g44450D	AT2G30770	cytochrome P450, family 71, subfamily A, polypeptide 13 (CYP71A13)	0.00	inf	0.21	-0.82	0.00	-0.70	-0.08	0.00	0.00	0.00	-0.20	0.00	inf	0.00	-0.42	-0.11
6	BnaA03g44460D	AT4G20200	Terpenoid cyclases/Protein prenyltransferases superfamily protein	1.13	-1.61	0.87	0.00	-1.26	-1.26	-1.03	0.55	-0.56	2.88	0.09	0.54	0.48	-0.25	inf	0.00
6	BnaA03g44470D			0.00	0.00	0.00	0.00	0.00	0.00	0.00	0.00	0.00	0.00	0.00	0.00	0.00	0.00	0.00	0.00
6	BnaA03g44480D	AT4G20260	plasma-membrane associated cation-binding protein 1 (PCAP1)	-1.20	-0.66	-1.41	-1.44	-0.13	0.59	-0.67	-1.28	-0.93	0.12	0.66	2.55	0.38	-0.07	1.68	0.57
6	BnaA03g44490D			0.00	0.00	0.00	0.00	0.00	0.00	0.00	0.00	0.00	0.00	0.00	0.00	0.00	0.00	0.00	0.00
6	BnaA03g44500D	AT4G20270	BARELY ANY MERISTEM 3 (BAM3)	-0.01	1.10	-0.06	0.02	-0.26	-0.51	-0.68	-0.06	0.46	0.00	0.02	-0.18	-0.64	-0.15	0.02	-0.24
6	BnaA03g44510D	AT4G20320	CTP synthase family protein	1.70	-0.55	0.40	-1.22	0.31	0.51	-1.50	-0.28	-2.17	1.19	-0.19	-0.23	2.18	0.93	-0.22	-0.11
6	BnaA03g44520D	AT4G20325	FUNCTIONS IN: molecular_function unknown	1.15	1.15	0.96	0.19	0.09	0.15	1.75	0.77	-0.13	-0.24	-0.03	-0.48	-2.53	-0.68	-1.03	-1.26
6	BnaA03g44530D	AT4G20360	RAB GTPase homolog E1B (RABE1b)	0.08	0.48	-0.05	-0.55	-0.10	-0.35	-0.26	0.09	1.02	-0.51	-0.30	0.02	-0.55	-0.29	0.23	-0.04
6	BnaA03g44540D	AT4G20380	LSD1 zinc finger family protein	0.18	0.40	-0.55	-0.84	0.39	0.31	-0.98	-0.01	0.22	0.37	0.53	-0.51	-0.46	0.20	0.26	-0.35
6	BnaA03g44550D	AT4G20420	Tapetum specific protein TAP35/TAP44	0.59	1.59	1.23	-0.93	-0.59	0.32	-1.16	1.36	0.00	0.00	1.34	-0.22	inf	0.00	inf	inf
6	BnaA03g44560D			0.00	0.00	0.00	0.00	0.00	0.00	0.00	0.00	0.00	0.00	0.00	0.00	0.00	0.00	0.00	0.00
6	BnaA03g44570D	AT4G20780	calmodulin like 42 (CML42)	-0.11	0.25	0.08	-0.76	-0.65	-1.20	2.49	-0.02	1.41	0.36	0.13	0.11	-0.95	0.64	inf	-0.18
6	BnaA03g44580D	AT4G20790	Leucine-rich repeat protein kinase family protein	0.00	0.00	-0.96	0.00	0.00	0.00	0.00	0.33	0.00	0.00	-0.47	-0.37	inf	0.00	0.00	inf
6	BnaA03g44590D			0.00	0.00	0.00	0.00	0.00	0.00	0.00	0.00	0.00	0.00	0.00	0.00	0.00	0.00	0.00	0.00
6	BnaA03g44600D	AT4G20830	FAD-binding Berberine family protein	-0.95	-0.89	-0.58	1.10	-0.51	-0.26	2.12	-0.60	0.57	-0.47	-0.06	0.50	-0.32	0.85	-0.19	-1.24
6	BnaA03g44610D	AT4G20840	FAD-binding Berberine family protein	-0.39	-1.41	0.00	-0.54	-1.48	-0.40	-1.14	-0.42	-0.95	-1.11	0.46	-0.16	1.38	0.19	0.27	-0.40
6	BnaA03g44620D	AT4G20850	tripeptidyl peptidase ii (TPP2)	-0.27	-0.46	-0.58	-0.62	-0.63	-0.08	-0.90	-0.26	0.60	-0.60	-0.10	-0.30	0.08	-0.46	-0.24	0.54
6	BnaA03g44630D	AT4G20880	ethylene-responsive nuclear protein / ethylene-regulated nuclear protein (ERT2)	-1.06	-1.06	-0.82	-0.23	-1.03	2.13	-0.69	-0.78	0.32	-1.60	1.24	0.94	-0.86	0.20	-1.62	-0.03
6	BnaA03g44640D	AT4G20890	tubulin beta-9 chain (TUB9)	-0.37	0.10	-0.38	0.08	1.66	0.68	1.68	-0.33	-0.31	-1.38	0.37	-0.57	-0.55	-0.80	-0.39	0.23
6	BnaA03g44650D	AT4G20900	MALE-STERILE 5 (MS5)	0.00	0.00	0.54	0.26	-0.99	0.17	0.00	0.72	-2.30	0.00	-0.03	-0.23	-1.04	-0.76	-1.12	inf
7	BnaA03g54460D	AT4G39780	Integrase-type DNA-binding superfamily protein	0.61	0.67	-0.27	-1.69	-0.08	-0.09	-0.87	-0.21	0.42	-0.12	-0.92	0.47	1.47	-0.05	-0.37	-0.43
7	BnaA03g54470D			0.00	0.00	0.00	0.00	0.00	0.00	0.00	inf	0.33	0.44	-0.31	inf	-2.29	0.00	0.04	0.00
7	BnaA03g54480D			0.00	1.44	-0.19	-1.34	0.00	-2.73	-3.72	-0.65	-0.87	-1.52	-1.14	-4.58	-1.28	-1.52	-0.78	-2.20
8	BnaA05g29380D	AT3G08947	ARM repeat superfamily protein	0.54	0.02	0.15	-0.26	-0.18	0.01	-0.40	0.14	0.42	-0.46	-0.40	-0.79	-0.17	0.24	-0.70	0.52
8	BnaA05g29390D	AT3G08940	light harvesting complex photosystem II (LHCB4.2)	-0.10	0.43	0.18	-1.82	0.11	-0.39	-2.28	0.51	0.81	1.12	-0.72	-0.40	-0.25	0.03	0.03	0.05
8	BnaA05g29400D	AT3G08820	Pentatricopeptide repeat (PPR) superfamily protein	-0.48	-0.62	-0.30	1.18	-0.56	0.04	1.27	0.44	-0.19	-0.55	-0.06	-0.70	-0.80	-0.40	-0.37	0.99
8	BnaA05g29410D	AT3G08800	ARM repeat superfamily protein	-0.94	-0.71	-0.06	0.42	-0.14	-0.51	0.30	-0.12	0.82	-0.36	0.12	-0.58	-0.07	-0.06	-0.91	-0.07
8	BnaA05g29420D	AT3G08780	unknown protein	0.39	0.38	0.10	-0.26	-0.06	-0.10	-0.95	0.60	0.39	-0.31	-0.24	-0.59	0.19	0.37	0.05	0.51
8	BnaA05g29430D	AT3G08770	lipid transfer protein 6 (LTP6)	-1.92	-0.29	0.16	-1.89	-0.15	0.34	-1.34	0.19	-1.35	0.00	-0.15	-0.31	2.46	1.89	-1.88	0.24
8	BnaA05g29440D	AT5G38670	Galactose oxidase/kelch repeat superfamily protein	-0.04	-0.50	-0.04	0.02	-0.24	-0.10	0.34	0.54	-0.01	-0.20	-0.51	-0.44	-0.08	-0.51	-0.38	-0.07
8	BnaA05g29450D	AT5G48980	Galactose oxidase/kelch repeat superfamily protein	-0.11	-0.62	0.16	-1.14	-0.01	0.31	0.46	0.70	0.63	-0.43	-0.65	-0.46	-0.24	-0.13	-0.66	0.84
8	BnaA05g29460D	AT3G08760	ATSIK	0.35	-0.44	0.02	-0.06	-0.16	0.21	-0.11	-0.44	0.44	0.02	-0.13	-0.70	0.18	0.30	-0.50	0.28
8	BnaA05g29470D	AT3G08740	elongation factor P (EF-P) family protein	-0.02	1.09	-0.19	0.00	-0.05	-0.22	0.04	-0.15	0.63	-0.48	-0.07	-0.44	-1.00	-0.20	0.19	-0.59
8	BnaA05g29480D			0.00	0.00	0.00	0.00	0.00	0.00	0.00	0.00	0.00	0.00	0.00	0.00	0.00	0.00	0.00	0.00
8	BnaA05g29490D	AT3G08730	protein-serine kinase 1 (PK1)	0.62	0.68	0.14	-0.51	0.00	0.10	-1.32	0.19	0.80	-0.78	-0.33	-0.63	0.13	0.37	-0.36	0.07
8	BnaA05g29500D	AT3G08720	Arabidopsis thaliana protein kinase 19 (ATPK19)	0.22	-0.40	-0.26	0.05	-0.75	0.51	-0.71	-0.47	0.88	-0.74	-0.04	-0.32	0.40	-0.40	-0.66	-0.30
8	BnaA05g29510D	AT3G08710	thioredoxin H-type 9 (TH9)	-0.12	-0.09	0.00	-0.95	-0.08	-0.08	-2.63	-0.22	0.76	-0.33	-0.23	-0.91	-0.49	-0.51	-0.32	-0.68
8	BnaA05g29520D			0.00	0.00	-1.68	0.00	0.00	1.61	0.00	0.04	0.00	0.06	0.00	-0.91	0.00	0.00	0.90	0.00
8	BnaA05g29530D	AT3G08690	ubiquitin-conjugating enzyme 11 (UBC11)	0.24	0.03	-0.13	-0.35	0.07	0.20	0.02	0.07	0.04	0.01	-0.35	-0.68	0.05	-0.15	-0.27	-0.54
8	BnaA05g29540D	AT3G08680	Leucine-rich repeat protein kinase family protein	0.11	0.70	0.00	-2.18	-0.44	-0.34	-2.12	-0.16	0.13	-0.29	-0.44	-0.66	0.09	0.45	-0.60	-0.06
8	BnaA05g29550D	AT3G08670	unknown protein	0.72	-0.29	-0.15	-1.04	0.20	0.50	-0.76	-0.25	0.15	0.27	-0.55	-0.55	0.29	1.02	-0.50	0.36
8	BnaA05g29560D			0.00	0.00	0.00	0.00	0.00	0.00	0.00	0.00	0.00	0.00	0.00	0.00	0.00	0.00	0.00	0.00
8	BnaA05g29570D	AT3G08650	ZIP metal ion transporter family	0.50	0.11	0.26	-0.81	0.32	-0.12	-1.30	0.08	0.43	-0.01	-0.62	-0.39	-0.07	0.30	-0.34	0.03
8	BnaA05g29580D	AT1G17950	myb domain protein 52 (MYB52)	0.00	0.00	0.00	0.00	0.00	0.00	0.00	0.00	0.00	0.00	0.00	0.00	0.00	0.00	0.00	0.00

8	BnaA05g29590D	AT1G07440	NAD(P)-binding Rossmann-fold superfamily protein	0.00	0.00	0.00	0.00	0.00	0.00	0.00	inf	0.00	0.00	0.00	0.00	0.00	0.00	0.00	0.00
8	BnaA05g29600D	AT3G08630	Protein of unknown function (DUF3411)	0.53	0.33	0.24	-0.69	-0.37	0.22	-0.64	0.24	0.21	-0.46	-0.24	-0.20	1.95	0.35	0.15	0.43
8	BnaA05g29610D	AT3G08620	RNA-binding KH domain-containing protein	-0.46	0.25	0.41	-0.46	0.11	-0.13	-0.51	0.10	0.55	-0.34	0.00	-0.75	0.41	0.14	0.18	-0.44
8	BnaA05g29620D	AT3G08610	unknown protein	0.65	-1.24	0.74	-1.54	0.60	0.27	-1.01	0.72	1.45	-0.98	-0.30	-1.09	-0.07	0.22	-0.85	0.00
8	BnaA05g29630D	AT3G08600	Protein of unknown function (DUF1191)	-0.44	1.91	0.87	-0.49	0.66	-0.05	-1.98	0.38	0.17	-0.06	0.26	-1.12	0.24	-0.20	-0.28	-0.79
8	BnaA05g29640D	AT3G08590	Phosphoglycerate mutase, 2,3-bisphosphoglycerate-independent	0.57	0.23	0.05	-1.06	0.08	0.68	-0.92	0.35	1.09	-1.03	-0.55	-0.56	0.06	0.37	0.05	0.21
8	BnaA05g29650D	AT3G08550	KOBITO (KOB1)	0.62	-0.67	-0.22	-1.08	-0.07	0.46	-1.26	0.07	0.70	-0.79	-0.02	-1.09	0.16	-0.36	-1.00	-1.03
8	BnaA05g29660D	AT3G11130	Clathrin, heavy chain	-2.46	1.29	2.80	-2.80	-0.02	0.77	-1.11	2.46	-1.91	inf	-1.05	-1.56	1.92	inf	-0.85	-0.27
8	BnaA05g29670D	AT3G08510	phospholipase C 2	0.01	-0.19	0.23	-0.69	0.02	0.03	-0.65	0.00	0.41	-0.41	0.02	-0.35	0.21	-0.14	-0.22	-0.17
8	BnaA05g29680D	AT3G08500	myb domain protein 83 (MYB83)	0.00	0.00	-0.73	0.00	0.00	1.59	0.00	2.14	-0.20	0.12	0.27	-2.02	inf	1.11	0.00	0.00
8	BnaA05g29690D	AT3G08490	BEST Arabidopsis thaliana protein match is: Late embryogenesis abundant protein, group 2 (TAIR:AT3G24600.1)	-0.02	inf	0.00	0.00	0.00	0.00	0.00	-2.14	-0.67	0.70	0.54	0.00	0.00	inf	0.00	0.00
8	BnaA05g29700D	AT3G08040	FERRIC REDUCTASE DEFECTIVE 3 (FRD3)	0.70	-0.26	0.85	2.80	0.74	0.66	-1.62	0.67	2.18	-0.94	-0.31	-0.39	-0.74	-0.70	0.65	-0.34
8	BnaA05g29710D	AT3G08030	Protein of unknown function, DUF642	1.03	1.25	0.09	-2.31	1.04	1.13	-1.55	0.30	-0.07	0.18	-0.59	-0.92	0.19	1.70	1.10	0.87
8	BnaA05g29720D	AT3G08000	RNA-binding (RRM/RBD/RNP motifs) family protein	0.26	2.33	1.09	-0.73	0.40	0.09	0.04	0.13	1.88	-0.14	-0.59	-1.13	-1.21	1.28	-0.41	-0.70
8	BnaA05g29730D	AT3G07990	serine carboxypeptidase-like 27 (SCPL27)	0.69	1.18	0.25	0.00	0.22	-0.36	-0.62	0.06	0.22	-0.24	-0.21	-0.59	-0.61	0.15	-0.44	0.45
8	BnaA05g29740D	AT3G07970	QUARTET 2 (QRT2)	0.00	0.00	1.00	0.00	0.00	0.00	0.00	1.89	0.00	0.00	0.90	-1.82	0.00	0.00	0.00	0.00
8	BnaA05g29750D	AT3G07950	rhomboid protein-related	0.24	-0.36	0.16	-0.42	0.02	-0.05	-0.60	0.30	0.00	-0.16	-0.44	-0.68	-0.04	-0.34	-0.15	-0.47
8	BnaA05g29760D	AT3G07940	Calcium-dependent ARF-type GTPase activating protein family	0.31	0.50	0.08	0.12	0.07	0.58	-0.35	0.24	0.91	-0.51	-0.69	-1.18	0.07	0.27	-0.06	-0.25
8	BnaA05g29770D	AT3G07910	FUNCTIONS IN: molecular_function unknown	1.82	1.98	1.40	-0.15	2.80	-0.12	-0.94	0.79	1.46	-1.76	-1.95	-1.43	0.27	-0.26	0.77	1.48
8	BnaA05g29780D	AT3G07900	O-fucosyltransferase family protein	0.00	0.00	2.29	inf	0.00	0.00	0.00	1.67	0.00	inf	-0.24	-0.69	0.00	-1.50	0.00	0.00
8	BnaA05g29790D	AT3G07890	Ypt/Rab-GAP domain of gyp1p superfamily protein	0.97	0.31	0.35	-0.79	-0.11	0.68	-1.11	0.81	1.00	-0.63	-0.32	-0.64	-0.26	0.15	-0.26	0.34
8	BnaA05g29800D	AT3G07880	SUPERCENTIPEDE1 (SCN1)	0.05	0.49	-0.02	-0.83	0.15	0.16	-0.25	0.04	0.75	-0.26	0.11	-0.67	-0.34	-0.25	-0.17	-0.50
8	BnaA05g29810D	AT3G14040	Pectin lyase-like superfamily protein	-2.66	0.13	-0.81	-1.13	-0.12	-1.30	-0.45	-0.99	-0.22	-1.47	0.16	1.02	4.03	inf	5.04	inf
8	BnaA05g29820D	AT3G14040	Pectin lyase-like superfamily protein	0.31	-0.39	-0.64	-0.37	-0.54	-0.50	0.14	-1.03	-1.10	-3.26	0.02	0.18	-0.09	0.21	-0.84	-0.14
8	BnaA05g29830D	AT3G14040	Pectin lyase-like superfamily protein	0.31	-0.97	-0.46	-0.42	0.00	-0.37	-1.78	-0.87	2.45	-1.58	-0.30	-0.35	0.76	0.68	0.67	1.18
8	BnaA05g29840D	AT3G07810	RNA-binding (RRM/RBD/RNP motifs) family protein	0.69	0.84	0.10	-0.98	-0.41	0.17	-1.10	0.27	0.21	-0.68	-0.27	-0.74	0.43	0.26	-0.49	0.49
8	BnaA05g29850D	AT3G07800	Thymidine kinase	1.62	0.63	0.14	-2.13	2.74	0.00	-1.60	0.21	-0.38	-0.85	-0.13	-1.29	-2.12	-1.00	-0.52	-0.64
8	BnaA05g29860D	AT3G07790	DGCR14-related	0.00	0.07	-0.23	-0.76	0.35	0.39	-0.82	-0.02	0.76	-0.34	-0.26	-0.21	0.18	0.19	-0.23	0.22
8	BnaA05g29870D	AT3G07780	OBERON1 (OBE1)	inf	inf	0.00	2.14	0.29	-0.55	-0.47	0.87	2.33	inf	-0.28	-0.37	-0.48	-1.07	-0.09	0.40
8	BnaA05g29880D	AT3G07770	HEAT SHOCK PROTEIN 89.1 (Hsp89.1)	-0.24	-0.44	0.10	-0.14	-0.24	0.23	-0.36	-0.07	0.09	-0.30	-0.59	-0.99	-0.56	-0.30	-0.75	-0.38
8	BnaA05g29890D	AT3G07760	Sterile alpha motif (SAM) domain-containing protein	-0.29	-0.63	0.35	-0.90	-0.42	-0.35	0.25	-0.10	0.11	-0.52	-0.76	-0.66	0.33	-0.10	-0.15	-0.26
8	BnaA05g29900D	AT3G07750	3'-5'-exoribonuclease family protein	-0.09	0.70	1.13	0.02	-0.18	0.45	0.56	0.25	0.53	-2.49	-0.32	-1.00	0.49	-0.73	-0.20	1.17
8	BnaA05g29910D	AT3G07740	homolog of yeast ADA2 2A (ADA2A)	0.75	0.00	0.40	-1.11	0.23	0.26	-0.96	0.42	1.30	-1.06	-0.48	-1.42	0.19	-0.09	0.01	0.25
8	BnaA05g29920D	AT3G07730	unknown protein	0.00	-2.04	0.56	-1.45	0.14	0.04	-0.17	-1.08	0.00	-1.60	-0.79	-1.47	-0.94	-0.09	-0.90	inf
8	BnaA05g29930D	AT3G07720	Galactose oxidase/kelch repeat superfamily protein	-0.11	-0.40	-0.05	-0.69	-0.06	0.11	-0.82	-0.47	0.35	-0.80	0.09	-0.15	0.18	-0.08	-0.14	1.24
8	BnaA05g29940D	AT3G07700	Protein kinase superfamily protein	-0.43	0.02	0.08	-0.37	0.22	0.05	-0.03	0.07	1.38	-0.71	0.17	0.02	0.13	-0.02	0.24	-0.04
8	BnaA05g29950D	AT3G07690	6-phosphogluconate dehydrogenase family protein	0.56	0.04	0.32	-1.16	-0.12	-0.30	-0.46	0.50	0.37	-0.57	-0.60	-1.07	0.24	-0.02	-0.57	0.19
8	BnaA05g29960D	AT3G07680	emp24/gp25L/p24 family/GOLD family protein	0.12	-0.54	0.14	-0.25	-0.19	-0.04	-0.31	-0.08	-0.32	-0.40	-0.05	-0.77	0.05	-0.35	-0.56	-0.28
8	BnaA05g29970D	AT3G07660	Kinase-related protein of unknown function (DUF1296)	0.28	-0.33	0.05	-1.08	-0.29	0.26	-1.11	-0.07	0.36	-0.49	-0.41	-0.47	0.40	0.16	-0.01	-0.19
8	BnaA05g29980D	AT3G07650	CONSTANS-like 9 (COL9)	-0.34	-0.32	0.07	-0.75	0.20	-0.57	-0.17	-0.06	-0.24	-0.48	0.07	-0.79	-0.49	-1.32	-0.33	-0.39
8	BnaA05g29990D	AT3G07630	arogenate dehydratase 2 (ADT2)	-0.36	0.12	0.05	-0.23	-0.05	-0.34	0.20	0.10	0.53	-0.49	-0.30	-0.40	-0.01	-0.32	-0.13	-0.06
8	BnaA05g30000D	AT3G07610	increase in bonsai methylation 1 (IBM1)	0.54	-0.39	0.10	-0.96	-0.14	0.01	-1.48	-0.26	0.39	-0.66	-0.26	-0.74	0.10	-0.11	-0.60	-0.08
8	BnaA05g30010D	AT3G07600	Heavy metal transport/detoxification superfamily protein	-0.93	-0.76	0.00	-0.84	0.32	1.27	-0.55	inf	-1.13	0.00	0.00	0.40	0.40	-0.67	-0.63	-0.81
8	BnaA05g30020D	AT3G07590	Small nuclear ribonucleoprotein family protein	0.04	-0.43	0.24	-0.59	-0.05	-0.12	-0.51	0.35	0.24	0.12	-0.49	-0.71	-0.53	-0.32	-0.22	-0.03
8	BnaA05g30030D	AT3G07580	unknown protein	-0.23	-0.56	0.73	-1.74	-0.73	-0.80	-2.16	-0.07	-0.62	0.08	-0.44	-0.75	0.82	2.20	0.50	0.05
8	BnaA05g30040D	AT3G07570	Cytochrome b561/ferric reductase transmembrane with DOMON related domain	0.74	1.68	-0.12	-0.90	-1.70	0.66	-0.68	-0.20	1.30	0.52	-0.44	-1.08	0.51	0.83	0.98	1.41
8	BnaA05g30050D	AT3G07568	unknown protein	1.23	0.74	0.65	-0.26	-0.78	0.20	-0.19	1.26	1.29	-1.48	-0.60	-1.51	0.39	0.79	-0.28	0.36

8	BnaA05g30060D	AT3G07565	Protein of unknown function (DUF3755)	-0.54	0.13	0.48	-0.37	-0.11	-0.25	-0.39	-0.47	0.21	-0.79	-0.12	-0.43	0.23	0.08	-0.30	-0.58
8	BnaA05g30070D	AT3G07560	peroxin 13 (PEX13)	-0.12	-0.14	-0.13	-0.21	-0.06	0.01	-0.18	0.46	0.29	-0.34	0.16	-0.39	0.26	-0.42	-0.38	-0.14
8	BnaA05g30080D	AT3G07550	RNI-like superfamily protein	-0.44	-0.12	-0.11	-0.21	0.02	-0.77	-0.50	0.25	-0.02	0.10	0.05	-0.20	0.32	-0.39	-0.24	-0.38
8	BnaA05g30090D	AT3G07540	Actin-binding FH2 (formin homology 2) family protein	-0.23	inf	-0.32	-2.23	-2.45	1.24	-1.68	-0.22	-0.11	1.73	-0.57	-0.44	3.16	-0.05	0.90	-0.49
8	BnaA05g30100D	AT3G07530	CONTAINS InterPro DOMAIN/s: Beta-Casp domain (InterPro:IPR022712)	-0.89	0.98	0.10	-0.31	0.19	-0.52	-0.12	-0.04	0.55	0.51	0.79	-1.03	-0.29	-0.79	-1.37	-0.30
8	BnaA05g30110D	AT3G07525	autophagy 10 (ATG10)	-0.17	-0.49	0.27	-0.68	0.15	-0.23	-0.26	-0.43	-0.29	-0.03	0.20	-0.08	0.24	-0.88	-0.98	-0.40
8	BnaA05g30120D	AT3G07510	unknown protein	0.40	3.61	0.29	-0.44	0.44	-0.69	-1.35	0.16	-0.90	1.29	0.00	-0.62	-1.65	-0.92	0.04	-0.28
8	BnaA05g30130D	AT3G07500	Far-red impaired responsive (FAR1) family protein	-1.29	-0.40	-0.78	-0.81	0.95	0.94	0.00	-1.93	-0.90	2.92	2.59	-0.20	3.49	-0.08	1.08	-2.07
8	BnaA05g30140D	AT3G07490	ARF-GAP domain 11 (AGD11)	0.00	0.00	0.00	0.00	0.00	0.00	0.00	0.00	0.00	0.00	0.00	0.00	0.00	0.00	0.00	0.00
8	BnaA05g30150D			0.00	0.00	0.00	0.00	0.00	0.00	0.00	0.00	0.00	0.00	0.00	0.00	0.00	0.00	0.00	0.00
8	BnaA05g30160D	AT3G07480	2Fe-2S ferredoxin-like superfamily protein	-0.24	0.16	-0.27	-0.99	-0.01	0.10	-0.99	-0.32	-0.19	-0.02	-0.23	-0.01	0.34	0.33	0.44	0.38
8	BnaA05g30170D	AT3G07470	Protein of unknown function, DUF538	-0.15	0.76	-0.70	-0.84	-0.18	0.31	-1.06	-0.17	0.00	0.40	0.57	-0.54	-0.64	0.17	0.13	0.41
8	BnaA05g30180D	AT3G07450	Bifunctional inhibitor/lipid-transfer protein/seed storage 2S albumin superfamily protein	0.00	0.00	1.57	0.00	0.00	0.00	0.00	-0.42	0.00	0.00	0.62	-1.21	0.00	0.00	inf	0.00
8	BnaA05g30190D			0.00	0.00	0.00	0.00	0.00	0.00	0.00	0.00	0.00	0.00	inf	0.00	0.00	0.00	0.00	0.00
8	BnaA05g30200D	AT3G07420	asparaginyl-tRNA synthetase 2 (NS2)	-0.57	-1.95	-1.09	0.04	-1.04	-0.91	-2.24	0.03	-0.09	1.74	0.57	-0.01	-1.23	-0.38	-0.16	0.08
8	BnaA05g30210D	AT3G07400	lipase class 3 family protein	-0.30	-0.54	-0.58	-0.41	-0.03	-0.05	-0.90	-0.47	-0.06	-0.59	-0.27	-0.45	0.42	-0.49	-0.40	-0.20
8	BnaA05g30220D	AT1G07670	endomembrane-type CA-ATPase 4 (ECA4)	-0.16	0.13	-0.28	-0.65	0.37	-0.05	-1.03	-0.55	0.63	-0.79	-0.13	-0.49	0.42	-0.19	-0.26	0.01
8	BnaA05g30230D	AT3G07390	Auxin-Induced in Root cultures 12 (AIR12)	0.21	1.00	-0.66	-1.00	-1.15	-0.44	-0.02	-0.31	0.05	-0.22	-0.11	-0.49	0.36	-1.86	-0.83	-0.07
8	BnaA05g30240D	AT4G18100	Ribosomal protein L32e	0.00	0.00	0.00	0.00	0.00	0.00	0.00	0.00	0.00	0.00	0.00	0.00	0.00	0.00	0.00	0.00
8	BnaA05g30250D	AT3G07370	carboxyl terminus of HSC70-interacting protein (CHIP)	-0.67	-0.69	-0.24	-0.48	0.10	-0.07	-0.08	-0.19	0.15	-0.13	-0.02	-0.74	0.19	-0.61	0.15	0.04
8	BnaA05g30260D	AT3G07350	Protein of unknown function (DUF506)	-0.94	-0.16	-0.45	-1.00	0.52	0.58	-1.17	0.17	-0.29	0.50	0.52	1.00	0.57	-0.98	0.95	-0.03
8	BnaA05g30270D	AT3G07340	basic helix-loop-helix (bHLH) DNA-binding superfamily protein	-0.39	-0.97	-0.55	-0.45	-0.07	-0.81	-1.76	-0.13	0.81	-0.46	0.35	-0.46	0.29	0.56	2.60	1.69
8	BnaA05g30280D	AT3G07320	O-Glycosyl hydrolases family 17 protein	0.26	1.90	0.13	-1.99	-1.46	0.16	-2.01	-0.10	0.14	-0.28	-0.19	-0.15	-0.40	1.31	-0.05	-0.24
8	BnaA05g30290D	AT3G07310	Protein of unknown function (DUF760)	-0.20	-0.69	-0.34	-1.18	0.06	-0.16	-1.00	-0.35	0.50	-0.51	-0.30	-0.52	-0.64	-0.06	0.60	-0.37
8	BnaA05g30300D	AT3G07300	NagB/RpiA/CoA transferase-like superfamily protein	0.15	-0.42	0.05	-0.14	0.68	0.22	-0.01	-0.02	-0.07	-0.29	-0.16	-0.38	-0.13	-0.41	-0.02	-0.06
8	BnaA05g30310D	AT5G43490	unknown protein	-1.05	-1.08	0.41	-0.05	0.25	-0.60	0.76	-0.62	0.18	-0.63	0.56	-0.21	-0.22	-0.80	-0.49	-0.34
8	BnaA05g30320D	AT5G40260	Nodulin MtN3 family protein	0.00	0.00	0.48	0.00	1.05	0.39	0.00	2.83	0.02	0.00	-1.58	-0.71	-0.77	-2.69	0.00	0.00
8	BnaA05g30330D	AT3G07270	GTP cyclohydrolase I	-0.67	0.69	-0.46	-2.56	0.32	-0.49	0.29	-0.39	0.71	-0.12	0.78	0.24	-1.55	-0.32	1.17	-0.21
8	BnaA05g30340D	AT3G07220	SMAD/FHA domain-containing protein	-0.91	-0.34	-0.39	-0.61	-0.13	0.04	-0.15	-0.31	-0.41	0.16	-0.11	-0.81	0.25	-0.70	-0.46	-0.47
8	BnaA05g30350D	AT3G07210	unknown protein	0.14	-0.41	0.06	-1.22	-1.03	-0.52	0.03	-0.08	0.13	-0.26	-0.18	-0.48	-1.27	0.85	-0.44	-0.30
8	BnaA05g30360D	AT3G07200	RING/U-box superfamily protein	-0.11	-1.38	0.52	-0.04	-0.01	-1.14	-0.23	0.18	-0.29	0.11	-0.26	0.04	-1.12	-0.04	-0.44	-0.34
8	BnaA05g30370D	AT3G07195	RPM1-interacting protein 4 (RIN4) family protein	0.00	inf	0.00	0.00	0.00	0.00	0.00	0.00	0.00	0.00	0.00	0.00	0.00	0.00	0.00	0.00
8	BnaA05g30380D	AT3G07180	GPI transamidase component PIG-S-related	0.00	0.00	0.00	0.00	0.00	0.00	0.00	0.00	0.00	0.00	0.00	0.00	0.00	0.00	0.00	0.00
8	BnaA05g30390D	AT3G07180	GPI transamidase component PIG-S-related	-0.36	-0.51	-0.21	-0.79	-0.09	-0.22	-1.14	-0.48	0.11	-0.53	-0.14	-0.58	0.10	-0.17	-0.28	-0.31
8	BnaA05g30400D	AT3G07170	Sterile alpha motif (SAM) domain-containing protein	0.17	0.88	-0.10	-1.65	0.24	0.49	-0.30	-0.23	-0.02	0.67	-0.09	-0.69	0.16	0.44	-0.01	0.31
8	BnaA05g30410D	AT3G07160	glucan synthase-like 10 (GSL10)	-0.22	-0.48	-0.14	-0.61	-0.09	0.07	-0.88	-0.54	0.50	-0.70	-0.12	-0.70	0.27	-0.40	-0.45	-0.11
8	BnaA05g30420D	AT5G67290	AT3G07390	-0.33	0.07	-0.12	-0.23	-0.33	-0.64	-0.91	-0.33	0.23	0.31	-0.28	-0.30	0.39	-0.08	-0.14	-0.74
8	BnaA05g30430D	AT3G07150	unknown protein	-0.37	1.22	-0.37	-1.61	0.58	0.11	-0.04	-0.52	0.32	-2.12	-0.42	0.22	0.52	-0.77	-0.06	-0.08
8	BnaA05g30440D	AT3G07140	GPI transamidase component Gpi16 subunit family protein	-0.15	-0.51	-0.26	-0.36	0.02	-0.18	-0.50	-0.45	0.31	-0.57	-0.34	-0.68	-0.16	-0.49	-0.35	-0.33
8	BnaA05g30450D	AT3G07130	purple acid phosphatase 15 (PAP15)	-0.33	0.05	-0.35	-0.24	-0.15	-0.19	-0.22	-0.55	-0.74	-0.61	-0.87	-0.54	-0.10	-0.71	-0.45	0.00
8	BnaA05g30460D	AT3G07120	RING/U-box superfamily protein	-1.30	-2.41	-1.25	-1.36	-1.48	-2.35	-2.13	-1.24	1.02	-0.29	0.48	1.00	-0.50	1.17	-0.33	-0.48
8	BnaA05g30470D	AT3G07100	ENDOPLASMIC RETICULUM MORPHOLOGY 2 (ERMO2)	-0.39	-0.34	-0.38	-0.80	0.22	-0.03	-0.73	-0.53	0.19	-0.41	-0.30	-0.52	0.27	-0.40	-0.44	-0.13
8	BnaA05g30480D	AT3G07080	EamA-like transporter family	0.00	0.00	0.00	0.00	0.00	0.00	0.00	0.00	0.00	0.00	0.00	0.00	0.00	0.00	0.00	0.00
8	BnaA05g30490D	AT3G07060	embryo defective 1974 (emb1974)	-0.20	0.00	0.08	0.22	-0.47	-0.58	-0.36	-0.42	0.35	-0.59	0.42	-0.96	-0.17	-0.23	-0.28	0.03
8	BnaA05g30500D	AT3G07050	GTP-binding family protein	-0.75	-0.54	-0.09	-0.31	-0.07	-0.14	-0.16	-0.26	0.35	-0.63	0.13	-0.70	0.14	-0.26	-0.33	0.20
8	BnaA05g30510D			0.00	0.00	0.00	0.00	0.00	0.00	0.00	0.00	0.00	0.00	0.00	0.00	0.00	0.00	0.00	0.00

8	BnaA05g30520D	AT3G07020	UDP-Glycosyltransferase superfamily protein	-0.53	-0.25	-0.25	0.03	-0.16	-0.04	0.33	-0.48	0.23	-0.01	0.20	-0.20	0.21	-0.17	-0.51	0.01
8	BnaA05g30530D	AT3G07010	Pectin lyase-like superfamily protein	2.21	1.06	-0.78	-0.69	-0.27	-0.86	-1.56	-1.06	-0.56	0.94	0.75	0.22	0.24	1.45	0.38	-0.40
8	BnaA05g30540D	AT3G06985	low-molecular-weight cysteine-rich 44 (LCR44)	0.00	0.00	0.00	0.00	0.00	0.00	0.00	0.00	0.00	0.00	0.00	0.00	0.00	0.00	0.00	0.00
8	BnaA05g30550D	AT3G06980	DEA(D/H)-box RNA helicase family protein	-0.09	0.17	-0.14	-0.45	-0.17	0.33	-0.59	-0.31	1.03	-0.37	0.10	-0.84	-0.35	-0.44	-0.44	-0.47
8	BnaA05g30560D	AT3G06970	RNA-binding (RRM/RBD/RNP motifs) family protein	0.00	inf	1.09	0.00	0.00	inf	0.00	1.31	0.00	-1.52	-0.03	-0.45	0.00	0.00	inf	inf
8	BnaA05g30570D			0.00	0.00	0.00	0.00	0.00	0.00	0.00	0.00	0.00	0.00	0.96	-0.94	inf	0.00	0.00	inf
8	BnaA05g30580D	AT3G06930	protein arginine methyltransferase 4B (PRMT4B)	-0.32	-0.10	-0.02	-0.33	-0.18	-0.22	-0.09	-0.26	0.33	-0.38	0.13	-0.34	0.04	-0.16	-0.64	-0.13
8	BnaA05g30590D			-0.25	0.00	-1.10	0.00	0.00	0.00	0.00	-2.02	0.00	0.15	-0.86	-2.51	0.00	-1.44	0.00	inf
8	BnaA05g30600D	AT3G06868	unknown protein	0.00	1.30	-0.19	0.07	1.11	-0.79	-0.04	0.12	inf	-1.12	0.11	-0.80	1.05	1.48	-0.69	-0.23
8	BnaA05g30610D	AT3G06860	multifunctional protein 2 (MFP2)	0.05	-0.33	-0.39	-0.10	0.03	0.18	-0.44	-0.62	-0.12	0.22	0.26	0.10	0.09	-0.32	0.02	-0.23
8	BnaA05g30620D	AT3G06830	Plant invertase/pectin methylesterase inhibitor superfamily	0.00	0.00	-3.06	0.00	inf	0.00	0.00	-3.32	inf	0.00	2.95	3.00	0.00	0.00	0.00	0.00
8	BnaA05g30630D	AT3G06810	IBA-RESPONSE 3 (IBR3)	-0.21	-0.14	-0.28	-0.82	-0.37	-0.35	-0.51	-0.51	-0.14	0.04	-0.17	-0.06	0.52	-0.45	0.13	-0.24
8	BnaA05g30640D	AT3G06780	glycine-rich protein	-0.61	-0.26	-0.03	-0.44	-0.61	0.53	-1.57	-0.36	0.45	-0.49	-0.16	-0.33	0.61	0.13	0.11	1.02
8	BnaA05g30650D	AT3G06760	Drought-responsive family protein	-0.29	-0.59	0.41	0.05	-0.33	1.37	0.68	-1.52	-0.67	-0.86	0.42	-0.90	1.60	-1.29	0.13	0.11
8	BnaA05g30660D	AT5G49230	HYPERSENSITIVE TO RED AND BLUE (HRB1)	-0.02	0.32	0.19	-0.11	0.46	0.38	-0.94	0.06	-0.03	-0.43	0.09	-0.77	1.27	-1.44	0.21	-0.02
8	BnaA05g30670D			0.03	1.30	-0.48	0.00	-0.98	-0.26	-0.78	-0.53	-0.30	-0.24	-0.59	-0.90	1.63	1.40	-1.42	0.91
8	BnaA05g30680D	AT3G06730	Thioredoxin z (TRX z)	-0.71	0.81	-0.01	-0.62	-0.25	-0.04	0.40	0.05	1.58	0.05	-0.30	0.36	0.03	0.16	0.31	0.12
8	BnaA05g30690D	AT3G06680	Ribosomal L29e protein family	-1.02	0.34	-0.21	-0.95	0.86	-0.63	-0.35	0.02	0.02	-0.52	-0.40	-0.72	-0.22	-0.77	0.00	-0.47
8	BnaA05g30700D	AT3G06670	binding	-0.36	-0.34	0.15	-0.53	0.07	-0.13	-0.46	-0.13	0.65	-0.12	-0.10	-0.55	0.06	-0.09	-0.32	-0.04
8	BnaA05g30710D	AT3G06710	FUNCTIONS IN: molecular_function unknown	0.00	0.00	0.00	0.00	0.00	0.00	0.00	0.00	0.00	0.00	0.00	0.00	0.00	0.00	0.00	0.00
8	BnaA05g30720D	AT4G32670	RING/FYVE/PHD zinc finger superfamily protein	0.50	-0.62	-0.38	-1.36	-0.74	0.84	-0.92	-0.96	-0.36	0.62	-0.82	-0.88	2.08	-1.61	-0.94	0.28
8	BnaA05g30730D	AT3G66658	aldehyde dehydrogenase 22A1 (ALDH22A1)	-0.64	-0.03	-0.21	-1.04	-0.22	-0.18	-1.00	-0.35	0.28	-0.84	-0.14	-0.56	0.36	-0.15	0.00	-0.15
8	BnaA05g30740D	AT3G66654	Cyclophilin-like peptidyl-prolyl cis-trans isomerase family protein	-0.64	-0.16	-0.09	-0.34	0.52	0.30	-0.06	0.10	-0.10	0.06	-0.47	-0.96	-0.04	-0.91	-0.38	-0.82
8	BnaA05g30750D	AT3G06620	PAS domain-containing protein tyrosine kinase family protein	inf	0.00	0.00	inf	inf	0.00	4.27	0.00	0.00	0.00	0.00	-0.39	0.00	0.00	-1.25	0.00
8	BnaA05g30760D	AT3G06620	PAS domain-containing protein tyrosine kinase family protein	-0.49	-0.10	0.00	1.02	0.51	0.37	0.21	-0.05	0.39	-0.73	-0.15	-0.49	-0.21	-0.04	0.14	0.14
8	BnaA05g30770D	AT3G06610	DNA-binding enhancer protein-related	-0.51	0.60	-0.37	-0.73	-0.39	-0.54	0.39	-0.36	0.07	-0.93	-0.10	-0.47	-0.15	0.26	-0.04	-0.27
8	BnaA05g30780D	AT3G06600	unknown protein	0.00	0.00	-0.17	0.00	0.00	0.00	0.00	0.96	-2.35	0.00	-0.60	-0.79	0.00	0.00	inf	0.65
8	BnaA05g30790D	AT3G06590	basic helix-loop-helix (bHLH) DNA-binding superfamily protein	-0.55	-1.35	-0.46	-0.70	-0.20	-0.37	0.17	-1.08	-0.36	0.78	0.22	-0.45	-0.29	-0.21	1.29	-0.16
8	BnaA05g30800D	AT3G06580	GALK	0.13	-0.36	-0.40	-0.15	-0.12	-0.10	-0.51	-0.48	-0.24	0.06	-0.09	-0.58	0.02	-0.81	-0.48	-0.45
8	BnaA05g30810D	AT3G06560	poly(A) polymerase 3 (PAPS3)	-0.81	-1.39	0.54	-1.35	-0.46	0.86	-1.29	-0.23	1.17	-0.91	0.09	-0.77	1.21	0.31	0.18	-0.55
8	BnaA05g30820D	AT3G06550	O-acetyltransferase family protein	0.35	-0.40	0.12	-0.87	0.00	-0.26	-1.12	-0.15	0.39	-0.49	-0.18	-0.02	-1.41	0.42	-0.49	-0.68
8	BnaA05g30830D	AT3G06540	Rab escort protein (REP)	-0.32	-0.33	-0.44	-0.45	0.29	-0.05	-0.35	-0.59	-0.10	-0.16	-0.26	-0.54	0.19	0.07	-0.24	-0.06
8	BnaA05g30840D	AT3G06530	ARM repeat superfamily protein	-0.58	-0.65	-0.32	0.17	-0.47	-0.27	0.49	-0.91	0.63	-0.90	-0.23	-0.79	0.02	-0.33	-0.63	0.34
8	BnaA05g30850D	AT3G06510	SENSITIVE TO FREEZING 2 (SFR2)	-0.67	-0.38	-0.11	-0.73	0.04	0.15	-1.00	-0.18	0.58	-0.52	-0.52	-0.19	-0.40	-0.43	-0.36	-0.01
8	BnaA05g30860D	AT3G06500	Plant neutral invertase family protein	0.05	-0.37	-0.33	-0.33	-0.40	0.47	-0.65	-0.47	0.30	-0.59	-0.26	-0.83	-0.03	-0.84	-0.32	-0.02
8	BnaA05g30870D	AT3G06490	myb domain protein 108 (MYB108)	0.00	1.15	0.00	0.00	0.00	0.00	0.00	-3.00	inf	0.00	inf	inf	0.00	0.00	0.00	0.00
8	BnaA05g30880D	AT3G06483	pyruvate dehydrogenase kinase (PDK)	-0.73	-0.09	-0.37	0.11	0.38	0.19	0.14	-0.73	0.48	-0.34	0.09	1.15	0.92	0.74	-0.03	0.16
8	BnaA05g30890D	AT3G06470	GNS1/SUR4 membrane protein family	-0.06	0.27	-0.02	-1.57	-0.40	0.08	-1.58	-0.51	0.32	-0.11	-1.15	-0.89	-0.21	-0.01	-0.07	0.00
8	BnaA05g30900D			0.00	0.00	-1.18	0.00	0.00	0.00	0.00	-1.73	0.00	0.00	-1.96	-0.88	0.00	0.00	0.00	0.00
8	BnaA05g30910D	AT3G06430	embryo defective 2750 (EMB2750)	-0.44	0.20	-0.29	-0.50	-0.10	-0.26	-0.83	-0.59	0.60	-0.57	-0.25	-0.40	-1.17	-0.31	-0.49	0.07
8	BnaA05g30920D	AT3G06420	autophagy 8h (ATG8H)	-0.03	-1.46	0.00	0.69	-0.39	0.06	-0.41	0.00	0.27	0.68	-0.87	0.00	-1.36	-0.69	-0.74	-1.11
8	BnaA05g30930D	AT3G06410	Zinc finger C-x8-C-x5-C-x3-H type family protein	-0.15	-0.21	-0.32	-1.14	0.06	-0.10	-1.50	-0.52	0.29	-0.58	-0.52	-0.65	0.37	0.08	-0.13	-0.06
8	BnaA05g30940D	AT3G06400	chromatin-remodeling protein 11 (CHR11)	-0.37	-0.35	-0.11	-0.85	-0.04	0.01	-0.90	-0.22	0.29	-0.64	-0.29	-0.67	0.13	0.31	-0.31	0.15
8	BnaA05g30950D	AT3G06390	Uncharacterised protein family (UPF0497)	0.00	-1.31	0.00	0.30	0.00	-0.28	-0.76	0.00	0.00	0.00	0.00	inf	inf	0.00	-0.12	4.47
8	BnaA05g30960D	AT1G26540	Agenet domain-containing protein	-1.77	-2.18	0.12	-1.17	-3.16	-0.40	-1.64	-0.05	-0.51	0.40	0.01	-1.37	2.14	1.54	-0.07	-0.43
8	BnaA05g30970D	AT3G06380	tubby-like protein 9 (TLP9)	0.13	-0.04	0.41	-0.26	-0.01	0.09	0.02	-0.27	0.17	-0.48	-0.18	-1.23	-0.58	0.23	-0.91	0.19
8	BnaA05g30980D	AT3G06370	sodium hydrogen exchanger 4 (NHX4)	-0.47	-0.13	-0.20	-0.08	-0.41	0.29	-0.50	-0.84	-0.55	-0.74	0.52	0.17	2.74	0.25	-0.45	-0.55

8	BnaA05g30990D	AT3G06350	MATERNAL EFFECT EMBRYO ARREST 32 (MEE32)	0.00	inf	0.46	0.22	2.40	1.13	0.00	0.59	inf	-0.23	0.71	0.41	0.00	inf	-0.83	0.00
8	BnaA05g31000D	AT3G06340	DNAJ heat shock N-terminal domain-containing protein	-0.22	0.11	-0.08	-1.14	0.27	0.41	-1.04	0.08	0.49	-0.25	-0.50	-0.46	-0.06	-0.36	-0.48	0.64
8	BnaA05g31010D	AT3G06330	RING/U-box superfamily protein	-0.73	-0.49	-0.11	0.40	-0.04	-0.10	-0.13	-0.27	0.23	-0.05	-0.29	-0.65	0.35	-0.11	-0.59	-0.49
8	BnaA05g31020D	AT1G03710	Cystatin/monellin superfamily protein	0.00	inf	2.31	0.00	inf	0.00	0.00	0.70	0.00	0.00	1.40	1.37	0.00	0.00	-0.04	0.00
8	BnaA05g31030D	AT3G06300	P4H isoform 2 (AT-P4H-2)	-0.24	0.38	-0.43	-0.77	0.23	-0.23	-0.43	-0.11	0.04	0.23	-0.13	-0.44	-0.76	0.21	-0.68	0.04
8	BnaA05g31040D	AT3G06290	SAC3/GANP/Nin1/mts3/eIF-3 p25 family	-0.03	-1.32	-0.22	-1.26	-0.59	-0.39	-0.82	-0.99	0.62	-0.65	-1.00	-1.37	-0.10	-0.98	-0.87	-0.28
8	BnaA05g31050D	AT3G06290	SAC3/GANP/Nin1/mts3/eIF-3 p25 family	-0.50	-1.09	-0.43	-0.81	-0.24	-0.47	-0.81	-0.65	0.31	0.06	0.40	-0.90	-1.32	0.07	-1.13	-0.13
8	BnaA05g31060D	AT3G06170	Serinc-domain containing serine and sphingolipid biosynthesis protein	-0.37	-0.36	-0.30	-0.33	0.03	0.20	-0.85	-0.37	0.48	-0.57	-0.15	-0.51	-0.06	-0.47	-0.38	-0.09
8	BnaA05g31070D	AT3G06190	BTB-POZ and MATH domain 2 (BPM2)	-0.34	-0.03	0.15	-0.13	-0.04	0.32	-0.53	-0.14	0.47	0.03	-0.22	-0.35	0.18	-0.22	-0.17	0.14
8	BnaA05g31080D	AT3G06200	P-loop containing nucleoside triphosphate hydrolases superfamily protein	-0.62	0.21	-0.16	-0.62	0.05	-0.09	-0.98	-0.40	1.00	-0.47	-0.19	-0.57	0.06	-0.32	-1.08	-0.10
8	BnaA05g31090D	AT2G31470	DROUGHT TOLERANCE REPRESSOR (DOR)	0.00	0.00	0.00	-0.68	0.00	0.00	-1.56	inf	0.00	0.00	0.00	0.14	0.00	0.00	-0.76	-1.03
8	BnaA05g31100D	AT3G06210	ARM repeat superfamily protein	1.54	0.00	1.04	1.83	-1.18	0.10	-1.59	-2.80	3.48	0.00	1.13	-1.06	-0.50	-1.41	0.00	0.00
8	BnaA05g31110D	AT3G06230	MAP kinase kinase 8 (MKK8)	0.00	0.00	1.05	inf	0.00	0.00	0.00	2.94	0.00	0.00	0.82	-1.81	0.00	0.00	0.00	0.00
8	BnaA05g31120D	AT3G06150	unknown protein	-0.46	0.32	-0.31	0.24	-0.48	0.20	-1.37	-0.42	0.04	-0.33	-0.04	-0.63	-0.41	0.55	0.80	0.55
8	BnaA05g31130D	AT3G06145	unknown protein	-0.67	0.44	0.10	-0.72	0.61	-0.17	-0.38	-0.56	-0.69	-0.38	0.47	-0.33	-0.09	0.91	0.54	-1.61
8	BnaA05g31140D	AT3G06140	RING/U-box superfamily protein	-0.75	1.92	-0.25	-1.19	-1.75	-1.00	-1.04	-0.90	-0.41	-0.64	-0.43	0.26	0.81	1.31	-0.06	-0.90
8	BnaA05g31150D	AT3G06130	Heavy metal transport/detoxification superfamily protein	-0.30	0.38	-0.29	0.03	0.81	-0.62	-0.04	-0.34	0.52	-0.69	0.25	-0.65	-0.62	0.42	-0.08	-0.55
8	BnaA05g31160D	AT3G06120	MUTE (MUTE)	0.00	0.00	0.65	-1.10	0.00	0.00	-1.19	1.97	0.00	0.00	-0.36	-2.26	0.00	0.00	-0.28	-0.71
8	BnaA05g31170D	AT3G06110	MAPK phosphatase 2 (MKP2)	-0.24	-0.28	-0.70	-0.70	-0.05	-0.17	-0.86	-0.74	0.64	-1.06	-0.30	-0.94	-0.36	-0.82	-0.87	-0.31
8	BnaA05g31180D	AT3G06100	NOD26-like intrinsic protein 7	0.00	-0.69	0.72	0.00	-0.12	-0.62	-1.35	0.47	-0.44	0.00	0.07	-0.23	inf	0.86	3.53	inf
8	BnaA05g31190D			0.00	0.00	0.00	0.00	0.00	0.00	0.00	0.00	0.00	0.00	0.00	0.00	0.00	0.00	0.00	0.00
8	BnaA05g31200D	AT3G06080	CONTAINS InterPro DOMAIN/s: Protein of unknown function DUF231, plant (InterPro:IPR004253)	-0.19	-0.81	-0.06	-1.60	0.04	-0.39	-1.25	-0.19	0.50	-0.91	-0.20	-0.17	0.23	-0.11	-0.12	0.00
8	BnaA05g31210D	AT3G06070	unknown protein	-0.94	-0.92	-1.62	-1.14	-0.25	-0.72	-1.60	0.18	0.09	0.59	-0.18	1.95	-0.31	-1.52	-0.59	-0.80
8	BnaA05g31220D	AT3G06060	NAD(P)-binding Rossmann-fold superfamily protein	-0.29	-0.36	-0.35	-0.91	0.04	-0.38	-0.48	-0.04	0.42	-0.41	-0.36	-0.33	0.23	0.09	-0.02	-0.09
8	BnaA05g31230D	AT3G06050	peroxiredoxin IIF (PRXIIF)	-0.15	0.08	0.03	0.02	-0.22	-0.07	0.28	0.10	0.68	-0.35	0.04	-0.10	-0.21	-0.32	-0.18	0.04
8	BnaA05g31240D	AT3G06040	Ribosomal protein L12/ ATP-dependent Clp protease adaptor protein ClpS family protein	-0.69	0.31	-0.21	-0.29	-0.22	-0.11	-0.10	-0.31	0.04	-1.04	-0.55	-0.84	0.61	0.10	-0.16	-0.17
8	BnaA05g31250D	AT3G06035	Glycoprotein membrane precursor GPI-anchored	0.27	1.15	-0.55	-0.52	-0.86	0.20	-0.60	-0.31	-0.41	-0.09	0.15	-0.35	1.27	1.14	-0.67	-0.29
8	BnaA05g31260D	AT3G06030	NPK1-related protein kinase 3 (NP3)	-0.36	1.64	-0.14	-0.55	-0.50	0.89	-0.24	-0.08	-0.34	-1.07	-0.28	-0.85	1.10	2.49	-0.81	0.85
8	BnaA05g31270D	AT3G06020	Protein of unknown function (DUF3049)	-0.65	0.00	0.00	0.00	0.00	0.00	0.00	0.06	-0.66	0.00	0.95	0.49	0.00	0.00	inf	0.00
8	BnaA05g31280D			0.00	0.00	0.00	0.00	0.00	0.00	0.00	0.00	0.00	0.00	0.00	0.00	0.00	0.00	0.00	0.00
8	BnaA05g31290D	AT3G06000	RNI-like superfamily protein	-1.01	inf	0.39	0.00	0.00	0.00	0.00	-2.05	1.65	-0.35	2.51	0.00	0.00	0.00	0.00	0.00
8	BnaA05g31300D	AT3G05990	Leucine-rich repeat (LRR) family protein	-0.26	-0.32	-0.32	-1.12	-0.23	-0.15	-0.67	-0.09	0.45	-0.84	-0.53	-0.20	0.13	0.21	0.14	0.00
8	BnaA05g31310D	AT3G05980	unknown protein	-0.59	0.00	0.34	-2.68	0.27	2.41	-0.78	-0.05	0.11	0.00	-0.14	-0.49	-1.86	1.08	0.09	0.49
8	BnaA05g31320D			0.00	0.00	0.00	0.00	0.00	0.00	0.00	0.00	0.00	inf	0.00	0.00	0.00	0.00	0.00	0.00
8	BnaA05g31330D	AT3G05975	Late embryogenesis abundant (LEA) hydroxyproline-rich glycoprotein family	0.00	0.00	0.00	0.00	0.00	0.00	0.00	0.00	0.00	0.00	0.00	0.00	0.00	0.00	0.00	inf
8	BnaA05g31340D	AT3G05970	long-chain acyl-CoA synthetase 6 (LACS6)	-2.90	-1.34	-0.69	-0.44	-4.58	-3.95	-1.61	-1.39	-1.56	0.14	-0.66	0.20	1.74	-3.16	1.40	-1.21
8	BnaA05g31350D	AT3G05960	sugar transporter 6 (STP6)	0.00	0.00	-1.24	0.00	-2.66	-1.31	-1.68	-1.21	-0.76	0.00	0.68	1.45	0.00	0.00	0.71	-0.29
8	BnaA05g31360D	AT3G05950	RmlC-like cupins superfamily protein	0.00	0.00	0.00	0.00	0.00	0.00	0.00	0.00	0.00	0.00	inf	0.00	0.00	0.00	0.00	0.00
8	BnaA05g31370D	AT4G31570	CONTAINS InterPro DOMAIN/s: Prefoldin (InterPro:IPR009053)	0.49	0.14	1.25	-1.29	0.13	0.67	-0.49	-0.05	0.96	-1.01	0.44	-0.89	-0.73	0.25	-0.77	0.05
8	BnaA05g31380D	AT3G05940	Protein of unknown function (DUF300)	-0.10	0.08	0.48	-0.89	0.33	0.27	-0.57	0.26	0.63	-0.33	0.16	-0.70	0.21	-0.71	-0.58	-0.55
8	BnaA05g31390D	AT5G26731	unknown protein	0.00	-0.38	0.00	-1.76	1.45	-0.61	-1.80	0.71	2.15	-2.86	-0.31	-2.82	-1.21	-0.64	-1.19	-0.39
8	BnaA05g31400D	AT3G05920	Heavy metal transport/detoxification superfamily protein	1.01	inf	0.64	inf	0.00	0.00	0.00	-2.64	-1.85	0.00	0.49	1.34	1.52	0.00	0.00	0.00
8	BnaA05g31410D	AT3G05910	Pectinacetylesterase family protein	-0.07	-0.22	-0.51	-0.54	0.25	-0.35	-0.78	-0.74	0.33	0.11	-0.02	-0.44	-0.22	-0.24	-0.23	-1.01
8	BnaA05g31420D	AT3G05900	neurofilament protein-related	-0.50	-1.00	-0.63	-0.25	-0.43	-0.24	-0.44	-0.34	0.35	0.69	0.69	-0.49	0.68	-1.23	-0.59	-0.10
8	BnaA05g31430D	AT5G48670	AGAMOUS-like 80 (AGL80)	0.21	0.00	0.00	0.00	0.00	0.00	0.00	-0.04	inf	0.30	0.00	0.00	-1.38	0.00	0.00	inf

8	BnaA05g31440D	AT3G05860	MADS-box transcription factor family protein	-0.24	0.00	0.00	0.00	inf	inf	0.00	0.00	0.00	0.00	inf	-0.21	-1.33	0.00	-0.17	0.00
8	BnaA05g31450D	AT3G05858	unknown protein	0.00	0.00	0.00	0.00	0.00	0.00	inf	0.00	0.00	0.00	0.00	0.00	0.00	0.00	0.62	inf
8	BnaA05g31460D	AT3G05840	ATSK12	-0.06	-0.20	-0.08	-0.50	0.50	-0.05	-0.67	0.03	0.63	-0.43	-0.26	-0.69	-0.18	-0.27	-0.72	-0.71
8	BnaA05g31470D	AT3G05830	FUNCTIONS IN: molecular_function unknown	0.08	-1.13	0.55	-1.85	0.71	1.09	-0.52	0.57	0.88	-0.74	0.33	-1.14	0.45	-0.37	-0.34	0.03
8	BnaA05g31480D	AT3G05800	AtBS1(activation-tagged BRI1 suppressor 1)-interacting factor 1 (AIF1)	0.00	0.81	0.78	inf	0.00	0.00	0.00	-1.32	2.48	0.07	0.26	1.39	2.63	0.00	inf	-1.08
8	BnaA05g31490D	AT3G05770	unknown protein	0.00	0.00	2.00	0.00	0.00	-1.30	0.00	2.48	0.00	0.00	-0.34	-0.12	0.00	0.00	-1.02	0.00
8	BnaA05g31500D	AT3G05750	unknown protein	-0.21	0.03	0.15	-0.60	-0.07	-0.16	-0.09	0.14	0.45	0.03	0.13	-0.66	0.11	-0.31	-0.56	-0.31
8	BnaA05g31510D			0.00	0.00	0.00	0.00	0.00	0.00	0.00	0.00	0.00	0.00	0.00	0.00	0.00	0.00	0.00	0.00
8	BnaA05g31520D	AT3G05740	RECQ helicase l1 (RECQI1)	-1.19	-0.77	0.49	-0.99	1.50	inf	-1.63	0.19	0.12	-1.94	0.31	-0.56	0.00	0.76	-0.61	0.94
8	BnaA05g31530D	AT3G05727	S locus-related glycoprotein 1 (SLR1) binding pollen coat protein family	0.00	2.72	inf	-4.15	0.00	0.00	-2.98	0.00	1.79	-0.57	0.00	-1.87	1.67	0.00	-0.12	-0.95
8	BnaA05g31540D	AT3G05725	Protein of unknown function (DUF3511)	0.00	-0.56	-0.42	0.00	0.00	0.00	0.00	-1.58	0.00	0.00	0.44	inf	0.00	0.00	0.00	0.00
8	BnaA05g31550D	AT3G05720	importin alpha isoform 7 (IMPA-7)	0.64	0.46	-0.27	-0.34	-0.05	0.16	-0.35	-0.05	0.62	-1.69	0.05	-0.79	-0.24	-0.55	-0.48	-0.45
8	BnaA05g31560D	AT3G05710	syntaxin of plants 43 (SYP43)	-0.05	0.02	-0.27	-0.62	0.27	0.42	-0.39	-0.24	0.36	-0.31	-0.16	-0.92	-0.41	-0.29	-0.47	-0.72
8	BnaA05g31570D	AT3G05670	RING/U-box protein	-0.35	-0.80	-0.41	-0.22	-0.22	0.03	-0.52	-0.25	0.24	-0.21	0.16	-0.46	0.21	0.36	-0.23	0.27
8	BnaA05g31580D	AT3G05620	Plant invertase/pectin methylesterase inhibitor superfamily	0.04	1.15	-0.24	inf	-0.97	-0.79	0.00	0.04	0.35	-0.18	0.10	-0.17	-2.10	inf	-1.20	inf
8	BnaA05g31590D	AT4G02330	ATPMEPCRB	0.00	inf	0.00	0.00	0.00	0.00	inf	0.00	0.00	0.00	inf	0.00	0.00	0.00	0.00	0.00
8	BnaA05g31600D	AT3G05610	Plant invertase/pectin methylesterase inhibitor superfamily	0.00	0.00	-0.58	0.00	0.00	0.00	0.00	-0.35	0.00	0.00	0.03	-0.99	0.00	0.00	0.00	0.00
8	BnaA05g31610D	AT3G05600	alpha/beta-Hydrolases superfamily protein	0.00	2.16	0.10	-2.67	-1.43	-0.39	-2.17	0.27	-1.55	0.00	-0.23	-0.56	3.89	0.00	-0.78	0.01
8	BnaA05g31620D	AT3G05590	ribosomal protein L18 (RPL18)	0.00	0.00	0.00	0.00	0.00	0.00	0.00	0.00	0.00	0.00	0.00	0.00	0.00	0.00	0.00	0.00
8	BnaA05g31630D	AT3G05580	Calcineurin-like metallo-phosphoesterase superfamily protein	0.11	-0.82	-0.98	0.87	0.02	0.41	1.99	0.32	0.60	0.41	0.46	0.08	0.54	-0.11	0.52	-2.17
8	BnaA05g31640D	AT5G27830	FUNCTIONS IN: molecular_function unknown	-1.44	0.60	0.48	0.00	0.03	-2.51	1.43	0.46	0.00	-1.03	-0.28	-1.35	0.40	1.69	1.91	-1.42
8	BnaA05g31650D	AT3G46950	Mitochondrial transcription termination factor family protein	-0.17	-0.05	-0.08	-0.33	-0.33	-0.13	-0.04	-0.23	-0.10	-1.11	-0.09	-0.85	-0.18	-0.12	-0.18	0.21
8	BnaA05g31660D	AT3G05570	unknown protein	-0.38	-0.43	0.13	-0.41	0.99	0.02	-0.16	0.06	-0.45	-0.85	0.16	-1.18	-0.41	-0.41	-0.20	-0.57
8	BnaA05g31670D	AT3G05560	Ribosomal L22e protein family	-0.48	0.56	-0.03	-0.57	-0.15	-0.15	0.05	0.10	-0.21	-0.18	0.00	-0.74	-0.15	-0.44	-0.48	-0.41
8	BnaA05g31680D	AT3G05545	RING/U-box superfamily protein	-0.02	-0.27	-0.17	0.07	-0.14	-0.32	-0.29	0.07	0.22	-0.07	-0.16	-0.66	0.27	-0.15	-0.21	-0.09
8	BnaA05g31690D	AT3G05545	RING/U-box superfamily protein	-0.19	0.00	-0.17	-2.24	1.85	0.65	0.63	0.35	1.19	0.49	-0.59	-0.63	-0.97	1.36	0.54	-1.54
8	BnaA05g31700D	AT3G05530	regulatory particle triple-A ATPase 5A (RPT5A)	-0.09	-0.17	-0.11	0.25	0.01	-0.25	0.56	-0.29	-0.19	-0.23	-0.27	-0.31	0.02	0.07	0.02	0.05
8	BnaA05g31710D			0.00	0.00	0.00	0.00	0.00	0.00	0.00	0.00	0.00	0.00	0.00	0.00	inf	0.00	0.00	inf
8	BnaA05g31720D	AT3G05500	Rubber elongation factor protein (REF)	0.09	-0.42	-0.53	0.24	-0.12	1.58	-0.46	-1.01	-0.61	1.17	-0.27	1.35	3.22	-1.60	-0.03	0.30
8	BnaA05g31730D	AT3G05490	ralf-like 22 (RALFL22)	0.85	0.00	0.32	-2.32	0.34	0.81	-2.33	0.47	-1.86	0.37	0.10	-1.53	4.03	0.35	0.48	-0.78
8	BnaA05g31740D	AT3G05480	RAD9	-0.20	-3.31	0.26	-0.27	-0.13	1.01	-0.94	0.49	-0.73	0.76	-0.63	-0.95	-1.30	-0.29	-0.18	-0.13
8	BnaA05g31750D	AT3G05470	Actin-binding FH2 (formin homology 2) family protein	0.13	0.99	0.04	-1.06	-1.78	-0.63	-2.41	0.21	-1.48	1.33	-0.37	-0.51	-0.69	1.20	0.28	0.35
8	BnaA05g31760D	AT3G05460	sporozoite surface protein-related	0.00	0.00	0.00	0.00	0.00	0.00	0.00	0.00	0.00	0.00	0.00	0.00	0.00	0.00	0.00	0.00
8	BnaA05g31770D	AT3G05450	CONTAINS InterPro DOMAIN/s: Cystatin-related, plant (InterPro:IPR006525)	0.02	-0.09	-0.22	0.03	0.37	-0.15	0.09	-0.06	0.70	-0.27	0.28	0.59	0.30	-0.14	-0.03	-0.13
8	BnaA05g31780D	AT3G05420	acyl-CoA binding protein 4 (ACBP4)	0.12	-0.05	-0.19	-0.49	0.24	-0.11	-0.90	-0.14	0.21	-0.24	0.18	-0.48	0.16	0.18	-0.15	-0.01
8	BnaA05g31790D	AT3G05410	Photosystem II reaction center PsbP family protein	-0.57	0.39	0.10	0.37	0.32	-0.01	-0.77	0.31	0.25	-0.86	-0.35	-1.09	-0.23	-0.13	-0.15	0.29
8	BnaA05g31800D	AT3G05400	Major facilitator superfamily protein	0.48	1.30	-1.80	0.27	-0.91	0.13	-0.42	-1.55	0.46	-0.56	-1.25	-0.11	-1.31	-0.38	-0.52	-0.30
8	BnaA05g31810D	AT3G05390	FUNCTIONS IN: molecular_function unknown	0.00	0.00	-2.47	inf	-1.52	0.00	3.49	0.00	0.00	0.00	1.02	0.00	0.27	inf	0.00	0.59
8	BnaA05g31820D	AT3G05380	ALWAYS EARLY 2 (ALY2)	-0.14	-0.10	-0.12	-0.05	0.06	-0.08	-0.46	-0.08	0.33	-0.90	-0.14	-0.79	-0.04	-0.05	-0.58	0.26
8	BnaA05g31830D	AT3G05350	Metallopeptidase M24 family protein	-0.14	0.32	-0.01	-0.46	0.29	-0.11	-0.69	-0.12	1.01	-0.67	-0.15	-0.74	-0.52	-0.22	-0.30	-0.04
8	BnaA05g31840D	AT3G05340	Tetratricopeptide repeat (TPR)-like superfamily protein	0.18	-0.16	0.30	-0.08	-0.39	0.04	0.07	0.22	-0.06	-0.87	-0.35	-0.85	0.59	-0.43	-0.48	0.11
8	BnaA05g31850D	AT3G05330	TANGLED (ATN)	-0.57	1.92	0.04	-3.59	0.14	-0.51	-3.99	0.08	0.15	0.15	-0.12	-1.31	0.72	-0.22	-1.87	0.60
8	BnaA05g31860D	AT3G05327	Cyclin family protein	-0.05	3.24	1.57	-0.30	0.81	-0.57	0.84	0.69	0.16	0.08	-0.80	-3.24	0.42	-3.52	0.15	-2.22
8	BnaA05g31870D	AT3G05320	O-fucosyltransferase family protein	-0.72	-0.75	-0.77	-0.19	-0.12	-0.03	-0.03	-0.26	0.54	-2.10	0.44	-0.55	0.03	-0.43	-0.68	0.18
8	BnaA05g31880D	AT3G05280	Integral membrane Yip1 family protein	0.09	-0.90	-0.38	0.21	-0.28	-0.33	-0.84	-0.44	0.45	-0.70	-0.53	-1.11	-0.13	-0.62	-0.54	0.49

8	BnaA05g31890D	AT3G05270	Plant protein of unknown function (DUF869)	0.43	-0.51	-0.54	-0.55	-0.22	-0.30	-0.71	-0.50	0.23	-0.10	-0.23	-0.71	-0.03	0.31	-0.35	0.05
8	BnaA05g31900D	AT3G05260	NAD(P)-binding Rossmann-fold superfamily protein	0.00	-0.68	-0.66	1.45	-0.60	-0.40	1.24	-0.43	0.00	0.00	inf	4.79	7.81	-0.59	-0.23	-0.59
8	BnaA05g31910D	AT3G05250	RING/U-box superfamily protein	0.20	-0.13	-0.07	0.63	0.57	-0.07	0.10	0.65	0.28	0.41	0.25	-0.94	-0.04	-0.43	-0.46	-0.48
8	BnaA05g31920D	AT3G05240	mitochondrial editing factor 19 (MEF19)	-0.15	-1.08	0.48	-2.01	0.12	0.99	-1.71	-0.58	inf	-1.56	0.07	-1.26	0.25	-0.69	-0.42	0.16
8	BnaA05g31930D	AT3G05230	Signal peptidase subunit	0.18	0.26	-0.22	-0.40	0.00	-0.48	-0.08	0.08	-0.07	0.12	-0.06	-0.32	-0.03	-0.38	-0.23	-0.51
8	BnaA05g31940D	AT3G05220	Heavy metal transport/detoxification superfamily protein	-0.38	-0.08	-0.04	0.47	-0.31	0.37	0.75	0.09	0.30	0.13	-0.01	-0.66	-0.15	-0.47	-0.35	0.26
8	BnaA05g31950D	AT3G05210	ERCC1	-0.34	-0.37	-0.04	0.37	0.21	0.47	-0.54	0.02	0.51	-0.90	-0.25	-0.75	-0.12	-0.10	-0.41	0.18
8	BnaA05g31960D	AT3G05200	ATL6	-1.17	0.42	-0.69	-1.79	0.30	0.09	-0.70	-0.31	0.82	-0.34	1.63	0.86	0.86	0.31	0.64	0.32
8	BnaA05g31970D	AT3G05180	GDSL-like Lipase/Acylhydrolase superfamily protein	-0.77	0.39	-0.31	0.18	0.66	-0.02	0.47	-0.02	-0.93	0.48	0.25	-0.80	0.40	0.23	-0.21	-1.30
8	BnaA05g31980D	AT3G05170	Phosphoglycerate mutase family protein	-0.44	-0.51	-1.14	0.02	0.36	0.50	0.14	0.03	-0.14	-0.54	0.62	0.41	1.51	-0.03	-0.16	0.62
8	BnaA05g31990D	AT3G05165	Major facilitator superfamily protein	-0.18	-0.32	-0.30	0.20	0.27	-0.09	-0.15	-0.16	0.41	-0.34	-0.34	-0.62	-0.11	-0.03	-0.18	0.03
8	BnaA05g32000D	AT3G05155	Major facilitator superfamily protein	0.00	0.00	0.00	0.00	0.00	0.00	0.00	0.00	0.00	0.00	0.00	0.00	inf	0.00	inf	inf
8	BnaA05g32010D	AT3G05155	Major facilitator superfamily protein	0.00	0.00	0.00	0.00	0.00	0.00	0.00	0.00	0.00	0.00	0.00	0.00	0.00	0.00	inf	0.00
8	BnaA05g32020D	AT3G05140	ROP binding protein kinases 2 (RBK2)	-0.48	0.00	0.67	0.00	inf	0.00	0.00	0.00	0.00	0.00	1.83	-1.93	0.00	0.00	1.31	0.00
8	BnaA05g32030D	AT3G05130	BEST Arabidopsis thaliana protein match is: Prefoldin chaperone subunit family protein (TAIR:AT5G27330.1)	-0.47	-0.16	-0.35	-0.19	-0.47	0.87	0.50	-0.77	0.42	-2.19	-0.50	-0.99	-0.86	-0.57	-0.28	0.31
8	BnaA05g32040D	AT3G05120	GA INSENSITIVE DWARF1A (GID1A)	-0.39	-0.61	-0.18	-0.08	-0.32	0.23	-0.04	-0.07	0.26	-0.55	-0.44	-0.80	-0.50	-0.06	-0.17	0.20
8	BnaA05g32050D	AT3G04960	FUNCTIONS IN: molecular_function unknown	-1.02	0.00	0.27	0.24	-0.28	-0.72	-2.01	-0.32	-0.31	-0.69	0.56	-0.48	-0.25	-0.76	-0.53	-0.53
8	BnaA05g32060D	AT3G42950	Pectin lyase-like superfamily protein	0.04	-0.05	0.12	-0.26	-0.65	0.11	-0.68	-0.02	0.04	0.10	0.07	-0.47	0.13	0.86	-0.09	0.24
8	BnaA05g32070D	AT3G05100	S-adenosyl-L-methionine-dependent methyltransferases superfamily protein	-0.35	-0.08	-0.38	-0.19	0.05	0.10	-0.52	-0.35	0.23	-0.68	-0.05	-0.40	0.03	0.18	-0.18	0.08
8	BnaA05g32080D	AT3G05090	LATERAL ROOT STIMULATOR 1 (LRS1)	-0.48	-0.17	-0.23	-0.09	0.24	0.09	-0.12	-0.46	0.47	-0.38	-0.64	-0.56	-0.02	-0.09	-0.43	0.00
8	BnaA05g32090D	AT3G05070	CONTAINS InterPro DOMAIN/s: mRNA splicing factor, Cwf18 (InterPro:IPR013169)	-0.66	-0.15	-0.14	-0.19	-0.04	-0.01	-0.01	-0.34	-0.37	-0.74	-0.56	-0.74	-0.26	-0.20	0.04	-0.06
8	BnaA05g32100D	AT3G05050	Protein kinase superfamily protein	-0.26	-0.60	0.12	-0.39	-0.02	0.24	-0.52	-0.39	0.20	-0.84	-0.11	-0.80	0.11	-0.47	0.00	-0.40
8	BnaA05g32110D	AT4G38940	Galactose oxidase/kelch repeat superfamily protein	0.00	0.00	0.00	1.28	0.28	-2.79	0.98	0.00	0.00	0.00	0.00	-1.92	4.90	0.00	-1.48	2.22
8	BnaA05g32120D	AT3G05040	HASTY (HST)	0.00	0.00	0.00	0.00	0.00	0.00	0.00	0.00	0.00	0.00	0.00	0.00	0.00	0.00	0.00	0.00
8	BnaA05g32130D	AT3G05030	sodium hydrogen exchanger 2 (NHX2)	-0.11	0.50	-0.28	-0.27	0.19	0.03	-0.34	-0.24	0.36	-1.34	-0.23	-0.44	-0.72	0.33	-0.81	-0.66
8	BnaA05g32140D	AT5G58340	myb-like HTH transcriptional regulator family protein	-0.77	-0.26	0.02	0.37	0.03	0.10	-0.23	-0.14	0.28	-0.38	-0.13	-0.67	-0.03	-0.04	-0.61	0.06
8	BnaA05g32150D	AT4G08630	unknown protein	0.00	0.00	-0.78	inf	0.00	0.00	0.00	-0.96	0.00	0.00	0.21	-0.11	0.00	0.00	0.00	1.12
8	BnaA05g32160D	AT3G05000	Transport protein particle (TRAPP) component	-0.35	-0.33	-0.39	-0.28	0.15	0.01	-0.19	-0.30	0.36	-0.85	0.02	-1.01	0.27	-0.19	-0.47	0.23
8	BnaA05g32170D	AT5G27220	Frigida-like protein	0.17	-0.28	0.29	0.56	-2.42	-3.33	-0.39	-0.21	-2.08	2.52	1.05	-0.31	1.25	-0.15	-0.26	-0.18
8	BnaA05g32180D	AT3G04980	DNAJ heat shock N-terminal domain-containing protein	-0.28	-0.38	-0.14	-0.30	-0.34	-0.16	-0.55	-0.21	0.88	-0.03	0.34	-0.97	0.58	-0.37	-0.78	0.01
8	BnaA05g32190D	AT3G04970	DHHC-type zinc finger family protein	-1.39	-1.22	0.27	-0.39	0.42	0.30	0.46	-0.06	-0.27	-0.61	-0.60	-0.90	-1.11	-0.52	-1.52	-2.05
8	BnaA05g32200D	AT3G04960	FUNCTIONS IN: molecular_function unknown	0.00	0.00	0.10	-2.09	-0.24	0.43	-2.09	-0.39	0.00	0.00	-2.45	-1.81	-0.95	-0.22	-0.50	-0.62
8	BnaA05g32210D	AT3G04960	FUNCTIONS IN: molecular_function unknown	0.00	0.00	inf	0.00	0.00	0.00	0.00	0.00	0.00	0.00	0.00	0.00	0.00	0.00	0.00	inf
8	BnaA05g32220D	AT3G04945	low-molecular-weight cysteine-rich 18 (LCR18)	0.00	0.00	0.00	0.00	0.00	0.00	0.00	0.00	0.00	0.00	0.00	0.00	0.00	0.00	0.00	0.00
8	BnaA05g32230D	AT3G04945	low-molecular-weight cysteine-rich 18 (LCR18)	0.00	0.00	0.00	0.00	0.00	0.00	0.00	0.00	0.00	0.00	0.00	0.00	0.00	0.00	0.00	0.00
8	BnaA05g32240D	AT3G02940	myb domain protein 107 (MYB107)	0.00	inf	2.42	-1.17	-0.83	1.17	-1.50	-0.41	0.00	0.00	0.00	inf	inf	0.00	0.41	-0.71
8	BnaA05g32250D	AT3G02950	THO7	-0.27	-0.14	-0.16	-1.40	-0.03	-0.29	-1.03	-0.24	-0.71	-0.23	0.00	0.97	0.48	0.05	0.27	-0.19
8	BnaA05g32260D	AT3G02970	EXORDIUM like 6 (EXL6)	-0.64	-1.70	-0.53	0.66	-0.65	0.79	0.11	-0.95	0.18	-0.61	0.47	0.25	-0.10	-0.76	0.07	-0.68
8	BnaA05g32270D			0.00	0.00	0.00	0.00	0.00	0.00	0.00	0.00	0.00	0.00	0.00	0.00	0.00	0.00	0.00	0.00
8	BnaA05g32280D	AT3G02990	heat shock transcription factor A1E (HSFA1E)	-0.57	-0.54	-0.41	-1.10	-0.51	0.31	-0.22	-0.42	0.25	0.48	0.32	-0.15	0.80	-0.47	0.53	1.43
8	BnaA05g32290D	AT3G02990	heat shock transcription factor A1E (HSFA1E)	-0.47	-0.36	-0.39	0.00	-0.74	-0.17	0.15	-0.16	0.92	0.64	1.13	-1.18	1.68	0.28	2.10	-0.16
8	BnaA05g32300D	AT3G03050	cellulose synthase-like D3 (CSLD3)	0.86	-0.77	-0.76	-0.30	-0.55	0.12	-0.86	-0.42	-0.60	-0.33	-0.35	0.11	1.06	0.52	0.46	-0.22
8	BnaA05g32310D	AT3G03060	P-loop containing nucleoside triphosphate hydrolases superfamily protein	-0.03	0.28	-0.07	0.01	-0.97	-0.65	0.02	-0.14	-0.56	-0.46	0.40	0.63	-0.45	-0.22	-0.04	0.85
8	BnaA05g32320D	AT3G03070	NADH-ubiquinone oxidoreductase-related	-0.12	-0.37	-0.95	-0.27	-0.73	-0.07	0.39	-0.43	-0.62	-0.07	0.12	0.15	0.40	-0.22	0.50	-0.32
8	BnaA05g32330D	AT3G03080	Zinc-binding dehydrogenase family protein	-0.64	-0.25	-0.70	-0.58	-0.08	0.37	-0.41	-0.17	-0.57	-0.18	0.00	0.62	-0.26	-0.07	0.60	-0.70
8	BnaA05g32340D	AT3G03090	vacuolar glucose transporter 1 (VGT1)	-0.50	0.19	-0.49	-0.57	0.77	0.72	0.64	-0.62	0.00	-0.29	-0.20	0.81	0.34	-0.57	0.47	-1.99
8	BnaA05g32350D	AT3G03100	NADH:ubiquinone oxidoreductase, 17.2kDa subunit	-0.18	0.15	-0.68	0.07	-0.04	0.28	0.19	-0.41	-1.13	-0.27	-0.16	0.20	0.28	-0.33	0.26	-0.47

8	BnaA05g32360D	AT3G03110	exportin 1B (XPO1B)	0.00	0.00	-1.02	-1.84	0.00	0.00	0.00	0.36	0.00	0.00	-0.19	0.79	inf	inf	-0.26	-1.55
8	BnaA05g32370D	AT3G03120	ADP-ribosylation factor B1C (ARFB1C)	-0.26	-0.61	-0.56	-0.09	-0.08	0.46	0.15	-0.56	-0.59	-0.72	-0.12	0.38	0.79	-0.74	0.47	-0.80
8	BnaA05g32380D	AT3G03140	Tudor/PWWP/MBT superfamily protein	-0.35	-0.31	-0.35	0.07	-0.62	0.44	-0.13	-0.24	-0.37	0.09	0.10	0.30	0.57	-0.30	-0.01	-0.64
8	BnaA05g32390D	AT3G03150	unknown protein	-0.09	-0.47	-0.37	-1.18	-0.24	-0.11	-1.45	-0.26	-0.49	-0.19	0.26	0.70	0.29	-0.15	0.59	-1.09
8	BnaA05g32400D	AT3G03160	FUNCTIONS IN: molecular_function unknown	-0.13	0.26	-0.40	-0.57	-0.56	-0.37	-0.32	0.06	-1.56	0.16	0.85	1.59	0.20	0.67	0.83	-0.16
9	BnaA07g02520D	AT2G17260	glutamate receptor 2 (GLR2)	-0.02	-0.58	-0.78	0.93	0.02	0.33	0.04	-0.78	0.60	-0.04	-0.13	0.51	-0.01	0.63	-1.24	-0.17
9	BnaA07g02530D	AT1G63670	FUNCTIONS IN: molecular_function unknown	0.00	0.00	-3.56	3.81	0.00	0.00	4.88	-2.70	0.00	inf	0.77	2.82	4.30	0.62	2.76	0.21
9	BnaA07g02540D	AT2G17370	3-hydroxy-3-methylglutaryl-CoA reductase 2 (HMG2)	-0.59	-0.65	-0.68	0.32	-0.08	-0.03	-0.26	-1.06	-2.83	0.35	0.50	1.46	4.78	0.66	0.87	0.19
9	BnaA07g02550D	AT5G65850	F-box and associated interaction domains-containing protein	0.00	0.00	0.00	0.00	0.00	0.00	0.00	inf	0.00	0.00	0.00	0.00	0.00	0.00	0.00	0.00
9	BnaA07g02560D	AT4G14790	ATSUV3	0.00	0.00	0.00	0.00	0.00	0.00	0.00	0.00	0.00	0.00	0.00	0.00	0.00	0.00	0.00	0.00
9	BnaA07g02570D			0.00	0.00	0.00	0.00	0.00	0.00	0.00	0.00	0.00	0.00	0.00	0.00	0.00	0.00	0.00	0.00
9	BnaA07g02580D	AT2G17390	ankyrin repeat-containing 2B (AKR2B)	0.27	0.58	0.09	0.44	0.45	0.16	0.43	0.17	-0.02	-0.05	-0.11	-0.10	0.01	-0.08	-0.14	0.13
9	BnaA07g02590D	AT5G16830	syntaxin of plants 21 (SYP21)	0.34	1.83	0.79	1.29	1.14	1.26	1.35	1.05	-2.08	-0.83	-0.01	1.03	1.36	0.17	-1.00	-0.87
9	BnaA07g02600D	AT2G21045	Rhodanese/Cell cycle control phosphatase superfamily protein	inf	inf	0.00	0.00	-0.72	inf	0.00	0.00	0.00	0.00	0.00	0.00	0.00	-0.19	0.00	0.00
9	BnaA07g02610D			-0.55	-0.58	-0.29	2.09	-0.31	-0.73	1.35	-1.30	inf	1.19	0.05	-0.38	2.56	2.74	-0.62	0.71
9	BnaA07g02620D	AT2G17450	RING-H2 finger A3A (RHA3A)	-0.04	-0.64	0.26	-0.09	-0.42	-0.27	-0.25	0.98	-0.60	1.39	0.25	0.15	0.97	-0.19	0.63	0.00
9	BnaA07g02630D	AT2G17470	Aluminium activated malate transporter family protein	0.00	-2.01	-0.52	0.00	2.31	0.00	0.00	-1.67	-0.72	1.84	-1.43	0.00	0.00	0.00	0.00	0.00
9	BnaA07g02640D	AT2G17480	MILDEW RESISTANCE LOCUS O 8 (MLO8)	1.80	0.58	-0.65	-0.53	0.97	1.07	-0.11	-1.22	-0.09	-1.44	1.19	-0.41	-0.12	-0.49	-0.01	2.02
9	BnaA07g02650D	AT3G45410	Concanavalin A-like lectin protein kinase family protein	0.00	0.00	0.00	0.00	0.00	0.00	0.00	0.00	0.00	0.00	0.00	0.00	0.00	0.00	0.00	0.00
9	BnaA07g02660D	AT1G36970	CONTAINS InterPro DOMAIN/s: Domain of unknown function DUF1985 (InterPro:IPR015410)	0.00	0.00	0.00	0.00	0.00	0.00	0.00	0.00	0.00	0.00	0.00	0.00	0.00	0.00	0.00	0.00
9	BnaA07g02670D	AT2G17500	Auxin efflux carrier family protein	-0.86	0.00	0.70	inf	0.18	1.60	inf	1.72	0.62	0.00	1.30	-0.12	-0.04	0.95	-0.54	0.58
9	BnaA07g02680D	AT2G17550	unknown protein	0.00	0.00	0.00	0.00	0.00	0.00	0.00	0.00	inf	0.00	0.00	0.00	inf	0.00	0.00	0.00
9	BnaA07g02690D	AT2G17525	Pentatricopeptide repeat (PPR) superfamily protein	-0.18	-0.44	0.16	0.70	-0.40	0.58	0.43	0.05	1.72	-1.04	1.01	0.21	1.18	0.06	-0.02	1.07
9	BnaA07g02700D	AT2G17530	Protein kinase superfamily protein	0.29	0.13	-0.04	0.63	-0.37	-0.01	0.15	-0.40	-0.04	0.03	0.35	0.44	0.41	0.50	-0.20	-0.12
9	BnaA07g02710D			0.00	0.00	0.00	0.00	0.00	0.00	0.00	inf	0.00	0.00	0.00	0.00	0.00	0.00	0.00	0.00
9	BnaA07g02720D	AT2G17540	unknown protein	0.43	0.44	0.00	-0.19	-0.59	0.26	0.02	0.36	0.66	0.20	-0.16	0.44	-0.13	0.02	-0.51	0.30
9	BnaA07g02730D	AT2G17550	unknown protein	0.28	-0.46	-0.21	0.21	-1.57	-1.19	1.32	-0.55	-0.09	0.12	-0.20	-0.12	-0.83	0.43	0.10	inf
9	BnaA07g02740D	AT2G17560	high mobility group B4 (HMGB4)	0.01	0.17	1.13	0.37	0.36	0.41	-0.31	0.88	0.68	0.60	-0.35	0.07	0.13	0.48	0.38	0.77
9	BnaA07g02750D			0.00	0.00	0.00	0.00	0.00	0.00	0.00	0.00	0.00	0.00	0.00	0.00	0.00	0.00	0.00	0.00
9	BnaA07g02760D	AT2G17650	AMP-dependent synthetase and ligase family protein	0.11	-0.16	0.65	-0.28	0.42	0.11	-0.18	0.47	-0.05	-0.32	0.16	0.19	-0.77	0.08	0.01	-0.39
9	BnaA07g02770D	AT2G17660	RPM1-interacting protein 4 (RIN4) family protein	0.00	0.00	0.00	inf	0.00	0.00	0.00	0.00	0.00	0.00	0.00	0.00	0.14	0.00	0.00	0.00
9	BnaA07g02780D			inf	0.00	0.00	0.00	0.00	0.00	0.00	0.00	0.00	0.00	0.00	0.00	0.00	0.00	0.00	0.00
9	BnaA07g02790D			0.00	0.00	0.00	0.00	0.00	0.00	0.00	0.00	0.00	0.00	0.00	inf	0.00	0.00	0.00	0.00
9	BnaA07g02800D			0.00	0.00	0.00	inf	0.00	0.00	0.00	0.00	0.00	0.00	0.00	0.00	0.00	0.00	0.00	0.00
9	BnaA07g02810D	AT1G25270	nodulin MtN21 /EamA-like transporter family protein	0.00	0.00	inf	0.00	0.00	0.00	0.00	-0.31	0.00	0.00	-0.08	0.00	0.00	0.00	0.00	0.00
9	BnaA07g02820D	AT1G47790	F-box and associated interaction domains-containing protein	0.00	0.00	0.11	0.00	0.00	0.00	0.00	0.00	0.00	0.00	0.00	0.00	0.00	0.00	0.00	0.00
9	BnaA07g02830D	AT1G47790	F-box and associated interaction domains-containing protein	0.00	0.00	0.00	0.00	0.00	0.00	0.00	0.00	0.00	0.00	0.00	0.00	0.00	0.00	0.00	0.00
9	BnaA07g02840D	AT1G47765	F-box and associated interaction domains-containing protein	0.00	0.00	inf	0.00	inf	0.00	0.00	0.00	0.00	0.00	0.00	0.00	0.00	inf	0.00	0.00
9	BnaA07g02850D			0.00	0.00	0.00	0.00	0.00	0.00	0.00	0.00	0.00	0.00	0.00	0.00	0.00	0.00	0.00	0.00
9	BnaA07g02860D	AT2G17700	ACT-like protein tyrosine kinase family protein	0.05	-0.70	1.04	0.48	-0.99	-0.36	0.86	0.29	0.38	0.66	-0.01	0.16	1.95	-0.43	-0.37	0.55
9	BnaA07g02870D	AT2G17705	unknown protein	-0.04	-1.08	-0.06	-0.17	-0.22	0.13	0.64	0.47	0.23	0.07	-0.21	0.67	0.62	-0.53	0.42	-0.23
9	BnaA07g02880D	AT2G17710	unknown protein	0.44	-0.42	-0.68	-0.23	0.22	0.14	-0.30	-0.26	0.10	0.21	0.18	0.35	0.57	-0.34	0.38	-0.72
9	BnaA07g02890D	AT2G17720	2-oxoglutarate (2OG) and Fe(II)-dependent oxygenase superfamily protein	0.18	0.14	-0.38	0.74	-0.46	-0.25	0.39	-0.46	-0.41	0.66	0.31	0.23	0.27	0.73	0.52	0.89
9	BnaA07g02900D	AT4G15430	ERD (early-responsive to dehydration stress) family protein	0.00	1.14	0.00	-0.75	inf	0.68	2.40	-1.67	0.00	0.00	0.00	0.00	0.00	0.30	-1.71	-2.91

9	BnaA07g02910D			0.00	0.00	0.00	0.00	0.00	0.00	0.00	0.00	0.00	0.00	0.00	0.00	0.00	0.00	0.00	0.00
9	BnaA07g02920D	AT4G35840	RING/U-box superfamily protein	0.26	-1.60	-1.97	1.89	-1.80	-0.76	0.61	-1.63	0.31	0.27	0.31	0.43	-0.19	0.80	-0.29	0.13
9	BnaA07g02930D	AT1G45616	receptor like protein 6 (RLP6)	0.00	0.00	0.00	0.00	0.00	0.00	0.00	0.00	0.00	0.00	inf	0.00	0.00	0.00	0.00	inf
9	BnaA07g02940D	AT1G45616	receptor like protein 6 (RLP6)	0.00	0.00	0.00	0.31	0.00	0.00	0.00	0.00	0.00	0.00	0.00	0.00	0.00	0.00	inf	0.00
9	BnaA07g02950D	AT2G35795	Chaperone DnaJ-domain superfamily protein	0.00	0.00	inf	inf	0.00	0.00	0.00	inf	0.00	inf	0.00	0.09	0.00	inf	0.00	inf
9	BnaA07g02960D	AT2G17760	Eukaryotic aspartyl protease family protein	0.20	-0.18	-0.06	0.65	0.45	0.57	0.23	-0.22	0.37	0.11	0.00	0.61	0.03	0.44	0.30	0.38
9	BnaA07g02970D			0.00	0.00	0.00	0.00	0.00	0.00	0.00	0.00	0.00	0.00	0.00	0.00	0.00	0.00	0.00	0.00
9	BnaA07g02980D			0.00	0.00	0.00	0.00	0.00	0.00	0.00	0.00	0.00	0.00	0.00	0.00	0.00	0.00	0.00	0.00
9	BnaA07g02990D	AT1G64870	unknown protein	0.00	0.00	0.00	0.00	0.00	0.00	0.00	0.00	0.00	0.00	0.00	0.00	0.00	0.00	-1.91	1.09
9	BnaA07g03000D	AT2G17770	basic region/leucine zipper motif 27 (BZIP27)	-0.44	0.00	-1.27	0.00	0.87	-0.09	1.25	-0.72	0.16	1.25	-0.27	-1.42	-0.07	-0.92	0.00	-0.42
9	BnaA07g03010D	AT2G17787	unknown protein	3.08	0.28	0.02	0.93	1.07	0.66	-0.15	2.21	0.83	0.28	1.56	0.87	0.16	0.15	-0.91	-1.01
9	BnaA07g03020D	AT2G17790	VPS35 homolog A (VPS35A)	0.33	-0.45	0.29	0.18	0.21	0.12	-0.21	0.19	-0.01	-0.17	0.09	-0.16	-0.05	-0.21	-0.30	-0.24
9	BnaA07g03030D	AT2G17800	Arabidopsis RAC-like 1 (ARAC1)	-0.53	-1.03	-1.75	-0.04	-0.43	-1.25	0.06	-1.30	-0.23	0.37	0.78	1.01	-0.32	0.29	0.07	-0.55
9	BnaA07g03040D	AT4G01830	P-glycoprotein 5 (PGP5)	0.00	0.00	3.42	0.00	0.00	0.00	0.00	3.08	0.00	inf	-1.54	-0.16	inf	0.00	inf	0.00
9	BnaA07g03050D	AT2G17820	histidine kinase 1 (HK1)	-0.20	0.69	0.11	-0.01	0.26	-0.49	-0.69	-0.45	0.48	0.16	-0.57	0.22	-0.17	0.08	0.31	0.56
9	BnaA07g03060D	AT2G17840	EARLY-RESPONSIVE TO DEHYDRATION 7 (ERD7)	1.17	0.90	0.51	0.31	-0.01	0.70	-0.17	0.58	-0.24	-0.16	-0.26	0.30	-0.41	0.15	-0.79	0.03
9	BnaA07g03070D	AT2G17220	Protein kinase superfamily protein	-0.74	-0.19	0.31	1.32	-0.36	0.20	-0.45	-0.54	0.14	-0.01	0.12	-0.44	-0.63	-0.32	1.91	0.20
9	BnaA07g03080D			0.00	0.00	0.00	0.00	0.00	0.00	0.00	0.00	0.00	0.00	0.00	0.00	0.00	0.00	0.00	0.00
9	BnaA07g03090D	AT2G17200	DSK2	-0.11	-0.27	-0.06	0.00	0.17	0.04	-0.32	-0.14	0.14	0.01	-0.45	0.30	0.26	-0.08	0.07	0.13
9	BnaA07g03100D			0.00	0.00	0.00	0.00	0.00	0.00	0.00	0.00	0.00	0.00	0.00	0.00	0.00	0.00	0.00	0.00
9	BnaA07g03110D	AT2G17180	C2H2-like zinc finger protein, DAZ1, DUO1-ACTIVATED ZINC FINGER 1	0.00	0.00	-2.35	0.00	0.00	-0.49	0.00	-0.55	0.00	0.00	0.79	4.13	0.00	0.00	0.00	0.00
9	BnaA07g03120D	AT1G65450	HXXXD-type acyl-transferase family protein	0.00	0.00	0.00	0.00	0.00	0.00	0.00	0.00	0.00	0.00	0.00	0.00	0.00	0.00	0.00	0.00
9	BnaA07g03130D	AT2G17150	Plant regulator RWP-RK family protein	0.17	-0.13	0.41	0.07	-0.22	-0.04	-0.21	-0.13	0.24	-0.27	-0.07	-0.07	-0.09	-0.12	-0.02	0.13
9	BnaA07g03140D	AT2G17140	Pentatricopeptide repeat (PPR) superfamily protein	-1.07	-0.71	0.18	0.14	0.32	0.60	0.38	-0.17	0.33	-0.06	0.37	0.06	-0.72	0.01	0.46	0.29
9	BnaA07g03150D	AT2G17130	isocitrate dehydrogenase subunit 2 (IDH2)	0.21	-0.05	-0.03	-0.17	0.44	0.03	0.18	-0.26	-0.01	0.08	0.20	0.40	-0.19	0.05	0.02	-0.28
9	BnaA07g03160D	AT2G13100	Major facilitator superfamily protein	0.67	0.36	0.16	0.97	0.69	-0.28	0.63	0.00	0.47	1.94	0.17	0.61	0.78	1.96	-1.20	0.73
9	BnaA07g03170D	AT2G17120	lysm domain GPI-anchored protein 2 precursor (LYM2)	0.21	0.65	0.41	0.12	0.44	0.64	-0.22	0.55	-0.17	0.39	-0.53	0.40	0.20	0.44	0.24	0.23
9	BnaA07g03180D	AT2G17080	Arabidopsis protein of unknown function (DUF241)	0.00	0.00	0.00	0.00	inf	0.00	0.00	0.00	0.00	0.00	0.00	0.00	0.00	0.00	0.00	0.00
9	BnaA07g03190D	AT2G17070	Arabidopsis protein of unknown function (DUF241)	0.00	0.00	0.00	0.00	0.00	0.00	0.00	0.00	0.00	0.00	0.00	0.00	0.00	0.00	inf	0.00
9	BnaA07g03200D	AT2G17040	NAC domain containing protein 36 (NAC036)	-0.76	-0.28	0.67	1.69	0.57	0.56	1.88	0.37	1.12	-1.46	0.20	0.74	0.05	-0.45	-0.15	1.14
9	BnaA07g03210D			0.00	0.00	0.00	0.00	0.00	0.00	0.00	0.00	0.00	0.00	0.00	0.00	0.00	0.00	0.00	0.00
9	BnaA07g03220D	AT2G17030	CONTAINS InterPro DOMAIN/s: F-box domain, cyclin-like (InterPro:IPR001810), Protein of unknown function DUF295 (InterPro:IPR005174)	0.00	0.00	0.00	0.00	0.00	0.00	0.00	0.00	0.00	0.00	0.00	0.00	0.00	0.00	0.00	0.00
9	BnaA07g03230D			0.00	0.00	0.00	0.00	0.00	0.00	0.00	0.00	0.00	0.00	0.00	0.00	0.00	0.00	0.00	0.00
9	BnaA07g03240D	AT2G17010	Mechanosensitive ion channel family protein	0.00	0.00	0.03	-2.39	-0.98	0.00	-1.30	-0.78	0.00	0.00	0.27	0.26	0.00	0.00	0.61	0.42
9	BnaA07g03250D	AT2G16990	Major facilitator superfamily protein	-1.39	-1.00	-0.35	3.35	-0.76	1.11	3.27	-0.58	-0.33	1.29	0.22	-0.18	1.07	-0.48	0.40	-0.52
10	BnaA08g16720D	AT4G39110	Malectin/receptor-like protein kinase family protein	-0.96	-0.58	-0.24	0.44	-0.77	-1.47	0.07	-0.96	-0.41	-0.46	0.84	1.35	0.04	0.46	-0.60	-0.85
10	BnaA08g16730D	AT4G39100	short life (SHL1)	-0.14	0.38	0.44	-0.14	0.12	-0.13	-0.29	-0.13	0.26	-0.38	0.12	0.26	0.24	0.59	0.10	-0.16
10	BnaA08g16740D	AT4G39090	RESPONSIVE TO DEHYDRATION 19 (RD19)	0.53	0.06	1.01	-0.74	0.07	-0.03	-1.23	0.61	0.19	-0.11	-0.02	0.50	0.67	-0.43	0.81	0.33
10	BnaA08g16750D	AT5G61160	anthocyanin 5-aromatic acyltransferase 1 (AACT1)	inf	-2.42	0.80	0.00	-0.23	inf	1.54	inf	0.00	inf	1.66	-0.92	1.42	-0.87	0.00	0.00
10	BnaA08g16760D	AT3G29680	HXXXD-type acyl-transferase family protein	inf	0.83	-1.28	1.19	0.77	inf	0.20	inf	-1.53	2.15	0.72	0.48	0.00	-0.01	inf	inf
10	BnaA08g16770D	AT4G39080	vacuolar proton ATPase A3 (VHA-A3)	0.50	0.17	-0.12	-0.06	0.33	-0.02	-0.20	-0.07	0.04	-0.08	0.23	0.35	-0.46	-0.07	-0.16	-0.04
10	BnaA08g16780D	AT4G39070	B-BOX DOMAIN PROTEIN 20, BBX20	0.68	0.65	3.28	0.59	0.64	-0.70	-0.40	4.13	0.89	-0.42	0.87	-0.28	-0.62	-0.54	-0.68	-1.31
10	BnaA08g16790D	AT4G39050	Kinesin motor family protein	0.76	0.57	0.63	0.27	0.79	0.28	0.15	0.61	0.37	-0.24	-0.30	0.10	0.31	0.42	-0.37	-0.03
10	BnaA08g16800D	AT4G34660	SH3 domain-containing protein	0.18	0.74	0.75	-0.16	0.46	0.05	0.50	0.54	0.17	-0.60	-0.34	-0.01	-0.25	-0.41	-0.21	0.23
10	BnaA08g16810D	AT4G38980	unknown protein	-0.18	-0.20	0.13	0.31	-0.01	0.43	1.03	-0.05	0.20	0.11	-0.26	-0.38	0.60	0.01	0.02	-0.25
10	BnaA08g16820D	AT4G38970	fructose-bisphosphate aldolase 2 (FBA2)	0.04	0.71	0.09	-1.58	0.17	-0.48	-1.27	0.24	1.23	-0.63	-0.18	0.46	-0.48	0.39	1.06	-0.35
10	BnaA08g16830D	AT4G38960	B-box type zinc finger family protein	-1.06	-0.56	-0.32	1.82	inf	0.00	1.23	-2.81	1.75	2.01	0.77	0.69	0.84	0.08	-0.49	-0.86
10	BnaA08g16840D	AT4G38910	basic pentacysteine 5 (BPC5)	-0.06	-0.71	0.61	-0.55	-0.58	-0.12	-0.73	0.42	-0.12	0.49	-0.04	-0.12	-0.18	0.27	-0.01	0.27

10	BnaA08g16850D	AT2G21237	unknown protein	0.00	0.00	0.31	inf	0.00	0.00	0.00	0.48	0.00	0.00	0.20	0.41	-0.70	0.00	0.00	0.00
10	BnaA08g16860D	AT4G38900	Basic-leucine zipper (bZIP) transcription factor family protein	0.75	1.43	0.43	-0.26	-0.99	2.44	-0.48	0.08	-0.47	0.22	-0.29	0.98	0.15	0.75	-0.16	1.06
10	BnaA08g16870D	AT4G38870	F-box and associated interaction domains-containing protein	0.00	0.00	inf	0.00	0.00	0.00	0.00	0.00	0.00	0.00	0.00	0.00	0.00	0.00	0.00	0.00
10	BnaA08g16880D	AT1G73490	RNA-binding (RRM/RBD/RNP motifs) family protein	-1.82	-5.66	-1.45	0.67	-3.48	-3.83	1.43	-1.44	-0.56	-1.00	-1.19	-1.60	-0.42	-0.07	-1.06	-1.48
10	BnaA08g16890D			0.00	0.00	0.00	0.00	0.00	0.00	0.00	0.00	0.00	0.00	0.00	0.00	0.00	0.00	0.00	0.00
10	BnaA08g16900D	AT4G38800	methylthioadenosine nucleosidase 1 (MTN1)	0.35	0.38	0.39	0.17	1.73	1.19	0.09	0.62	-0.15	0.30	0.04	0.33	0.65	-0.57	0.29	-0.18
10	BnaA08g16910D	AT4G38790	ER lumen protein retaining receptor family protein	0.17	0.07	0.34	0.10	0.26	-0.21	0.10	0.33	0.02	-0.14	-0.24	-0.07	0.18	-0.23	-0.08	-0.36
11	BnaA08g19200D	AT1G24706	THO2	-0.01	-0.29	0.01	0.37	0.03	-0.09	-0.09	-0.29	0.38	-0.34	-0.42	-0.05	0.14	0.03	-0.07	-0.02
11	BnaA08g19210D	AT1G67980	caffeoyl-CoA 3-O-methyltransferase (CCOAMT)	0.56	0.83	0.06	-0.59	-0.13	-0.45	0.27	0.27	0.05	-0.36	-0.15	-0.16	2.92	1.21	0.04	-0.13
11	BnaA08g19220D	AT1G24764	microtubule-associated proteins 70-2 (MAP70-2)	0.22	1.89	-0.83	-0.47	-0.48	1.38	-1.12	-1.08	0.61	-0.77	-1.11	0.44	2.67	1.21	-0.13	0.08
11	BnaA08g19230D	AT1G25145	UDP-3-O-acyl N-acetylglycosamine deacetylase family protein	-0.28	0.27	0.39	0.77	0.25	0.33	0.72	-0.33	0.39	0.36	0.55	-0.09	0.01	-0.21	-0.45	-0.59
11	BnaA08g19240D	AT1G25220	anthranilate synthase beta subunit 1 (ASB1)	0.28	-0.02	0.21	1.22	0.27	-0.01	1.09	0.37	0.70	0.31	-0.06	0.07	-0.17	0.17	-0.22	0.10
11	BnaA08g19250D	AT1G25250	indeterminate(ID)-domain 16 (IDD16)	0.52	-0.42	0.05	-1.02	-0.37	-0.45	-0.12	0.04	-0.61	0.44	-0.48	-0.06	0.07	0.58	-0.41	0.28
11	BnaA08g19260D	AT1G25260	Ribosomal protein L10 family protein	-0.81	-0.34	-0.04	0.24	-0.39	-0.40	0.67	0.17	-0.75	-0.27	0.14	-0.32	0.05	-0.38	-0.09	-0.24
11	BnaA08g19270D	AT1G68140	Protein of unknown function (DUF1644)	0.02	-0.09	-0.05	0.59	-0.04	0.01	0.18	-0.13	0.18	-0.22	0.21	0.30	0.46	-0.15	0.11	0.13
11	BnaA08g19280D	AT1G25275	unknown protein	-0.90	0.66	0.01	-0.18	-1.65	-0.70	-0.26	0.08	-1.15	0.23	0.33	0.09	-0.30	-0.48	1.08	-0.57
11	BnaA08g19290D	AT1G25280	tubby like protein 10 (TLP10)	0.20	0.08	-0.38	0.17	-0.05	0.16	-0.32	0.23	0.59	-0.21	-0.55	0.10	-0.19	0.05	-0.05	-0.20
11	BnaA08g19300D	AT5G05020	Pollen Ole e 1 allergen and extensin family protein	0.00	0.00	0.00	0.00	0.00	0.00	0.00	0.00	0.00	0.00	0.00	0.00	0.00	0.00	0.00	0.00
11	BnaA08g19310D	AT1G25290	RHOMBOID-like protein 10 (RBL10)	0.58	0.67	0.42	0.09	0.85	0.68	0.91	0.54	0.44	-0.23	0.42	-0.20	-0.25	-0.14	-0.16	0.12
11	BnaA08g19320D	AT3G43570	GDSL-like Lipase/Acylhydrolase superfamily protein	inf	0.00	1.09	inf	0.00	0.00	0.00	0.00	0.00	0.00	0.39	1.66	0.00	0.00	0.00	0.00
11	BnaA08g19330D	AT1G25300	Octicosapeptide/Phox/Bem1p family protein	0.00	0.00	inf	0.00	0.00	0.00	0.00	1.86	0.00	0.00	-1.52	inf	0.00	0.00	0.00	0.00
11	BnaA08g19340D	AT1G25380	NAD+ transporter 2 (NDT2)	0.25	0.24	-0.19	0.06	-0.32	0.17	0.19	-0.23	0.43	0.12	-0.41	0.01	0.05	-0.12	0.17	0.19
11	BnaA08g19350D	AT5G11780	unknown protein	0.00	0.00	0.46	-0.68	0.28	-1.27	-0.21	-1.26	-2.23	1.52	inf	0.00	0.00	inf	0.00	-2.88
11	BnaA08g19360D			0.00	0.00	-0.89	0.00	-0.50	0.00	inf	-1.22	inf	0.00	0.33	2.64	inf	0.00	-3.02	2.59
11	BnaA08g19370D	AT1G25370	Protein of unknown function (DUF1639)	0.22	-1.80	-1.00	0.84	-0.01	0.37	0.55	-0.07	-0.23	-0.97	0.81	-0.12	-0.03	0.20	0.28	0.13
11	BnaA08g19380D	AT1G25360	Pentatricopeptide repeat (PPR) superfamily protein	0.14	0.62	0.34	0.33	-0.30	-0.58	0.05	0.57	-0.35	-0.88	0.43	-0.58	-0.45	0.26	1.35	-0.04
11	BnaA08g19390D	AT1G25350	ovule abortion 9 (OVA9)	0.00	0.00	0.00	0.00	0.00	0.00	0.00	0.00	0.00	0.00	0.00	0.00	0.00	0.00	0.00	0.00
11	BnaA08g19400D	AT1G25350	ovule abortion 9 (OVA9)	0.00	0.00	0.00	0.00	0.00	0.00	0.00	0.00	0.00	0.00	0.00	0.00	0.00	0.00	0.00	0.00
11	BnaA08g19410D	AT1G25330	basic helix-loop-helix (bHLH) DNA-binding superfamily protein	0.00	0.00	-0.61	0.00	0.74	inf	0.00	-0.46	0.00	0.00	-0.59	-0.25	0.00	0.00	inf	0.00
11	BnaA08g19420D	AT1G25440	B-BOX DOMAIN PROTEIN 15, BBX15	-1.14	0.99	inf	-0.11	0.00	inf	0.62	1.07	0.00	-1.82	0.00	1.78	1.26	0.00	2.55	-0.23
11	BnaA08g19430D	AT1G25480	Aluminium activated malate transporter family protein	-1.27	-1.67	-0.36	-0.09	0.39	-0.31	-0.95	-0.17	-1.07	1.33	0.24	0.33	1.54	-1.51	0.21	-0.61
11	BnaA08g19440D	AT1G25490	ROOTS CURL IN NPA (RCN1)	0.69	0.42	0.19	0.56	0.44	0.01	0.91	0.12	0.16	-0.11	0.09	-0.04	0.45	0.14	-0.19	-0.35
11	BnaA08g19450D	AT1G25500	Plasma-membrane choline transporter family protein	0.38	-0.36	0.73	0.82	0.42	0.32	-0.17	0.71	0.50	0.51	0.40	-0.05	-0.10	-0.56	-0.22	-0.01
11	BnaA08g19460D	AT1G25520	Uncharacterized protein family (UPF0016)	0.18	0.07	0.44	0.32	0.37	0.14	-0.08	0.46	-0.04	-0.13	0.13	0.06	-0.03	-0.23	0.18	-0.33
11	BnaA08g19470D	AT1G25530	Transmembrane amino acid transporter family protein	-0.05	0.23	0.55	-0.37	0.18	0.33	-0.61	0.58	0.66	-0.78	-0.42	0.36	-0.41	0.50	0.53	0.08
11	BnaA08g19480D	AT1G25540	PHYTOCHROME AND FLOWERING TIME 1 (PFT1)	0.06	0.11	0.43	-0.08	0.22	-0.05	-0.34	0.16	0.36	-0.15	-0.18	0.07	0.20	0.33	0.01	0.21
11	BnaA08g19490D	AT1G25560	TEMPRANILLO 1 (TEM1)	-2.06	0.52	0.19	0.48	-2.67	1.20	1.71	-0.90	1.65	-0.24	0.57	-0.78	0.47	0.40	1.15	-0.32
11	BnaA08g19500D	AT1G22570	Major facilitator superfamily protein	0.00	inf	1.65	-0.35	-0.27	0.38	-0.46	1.25	0.00	0.00	0.32	inf	6.87	0.00	0.60	-0.65
11	BnaA08g19510D	AT1G72120	Major facilitator superfamily protein	1.37	0.94	-0.26	1.42	0.49	0.39	0.63	-0.46	1.91	-0.82	-0.25	0.23	-0.06	0.39	1.88	0.05
11	BnaA08g19520D	AT5G37460	Family of unknown function (DUF577)	0.00	0.00	2.82	0.00	0.00	inf	0.00	inf	0.00	0.00	-1.14	0.58	0.00	0.00	0.00	0.00
11	BnaA08g19530D	AT5G37470	Family of unknown function (DUF577)	0.00	0.00	0.64	0.00	0.00	inf	0.00	-0.32	0.00	0.00	1.15	0.12	inf	0.00	0.00	0.00
11	BnaA08g19540D	AT1G25682	Family of unknown function (DUF572)	-0.15	-0.07	0.20	0.28	0.17	0.31	0.71	0.52	0.14	0.12	0.29	-0.18	0.14	-0.02	-0.13	0.59
11	BnaA08g19550D	AT1G26090	P-loop containing nucleoside triphosphate hydrolases superfamily protein	0.48	0.55	1.31	-0.37	-0.19	-0.18	0.47	1.05	0.05	-0.05	0.21	0.12	-0.84	0.23	0.13	0.29
12	BnaA09g42360D			0.00	0.00	0.00	0.00	0.00	0.00	0.00	0.00	0.00	0.00	0.00	0.00	0.00	0.00	0.00	0.00
12	BnaA09g42370D	AT2G22760	basic helix-loop-helix (bHLH) DNA-binding superfamily protein	0.00	0.00	0.00	0.00	0.00	0.00	0.00	0.00	inf	0.00	0.00	inf	-1.21	inf	0.00	0.00
12	BnaA09g42380D	AT5G43745	Protein of unknown function (DUF1012)	-0.56	-0.81	-0.43	-2.19	-1.17	-1.79	-1.04	-0.80	0.86	-0.99	0.10	-0.51	-1.99	-0.48	-0.22	-0.57

12	BnaA09g42390D	AT2G22750	basic helix-loop-helix (bHLH) DNA-binding superfamily protein	0.00	0.90	1.51	-0.77	-1.07	0.06	-2.15	-1.28	-0.01	-1.03	1.22	2.14	-0.42	2.39	-1.60	0.22
12	BnaA09g42400D	AT2G22720	SPT2 chromatin protein	0.05	0.05	0.14	0.01	0.09	0.00	-0.17	0.11	0.09	0.10	-0.17	0.35	0.09	0.36	-0.16	0.41
12	BnaA09g42410D	AT2G22640	BRICK1 (BRK1)	0.38	inf	0.00	0.00	0.46	0.66	0.00	1.11	1.49	0.00	0.00	0.00	inf	-0.09	0.00	0.00
12	BnaA09g42420D	AT2G22620	Rhamnogalacturonate lyase family protein	0.90	1.08	0.83	-0.24	-0.20	-0.40	-1.01	0.43	-0.03	0.58	-0.08	0.05	3.50	1.30	0.32	-0.19
12	BnaA09g42430D	AT2G22610	Di-glucose binding protein with Kinesin motor domain	0.38	1.25	-0.12	-1.14	-1.03	-0.90	-0.78	-0.39	-0.02	-0.01	0.27	-0.11	0.71	1.29	-0.49	-0.02
12	BnaA09g42440D	AT2G22600	RNA-binding KH domain-containing protein	-0.57	-0.66	0.41	-0.84	0.18	-0.23	-1.26	-0.09	0.70	-0.09	0.21	0.01	0.91	0.05	0.30	0.56
12	BnaA09g42450D	AT2G22570	nicotinamidase 1 (NIC1)	-0.48	-0.11	-0.29	0.76	-0.17	0.30	0.21	-0.02	-1.02	0.61	0.04	1.02	-0.38	-0.18	-0.53	0.86
12	BnaA09g42460D	AT2G22560	Kinase interacting (KIP1-like) family protein	0.00	0.00	0.98	0.00	0.00	0.00	0.00	-0.22	-1.80	1.03	inf	-1.68	0.00	2.41	0.00	inf
12	BnaA09g42470D	AT2G22560	Kinase interacting (KIP1-like) family protein	-1.88	-2.10	-0.70	-0.83	-0.94	-1.22	1.83	-1.29	-0.19	1.52	0.22	-0.52	-0.36	0.44	-1.58	2.40
12	BnaA09g42480D	AT2G22540	SHORT VEGETATIVE PHASE (SVP)	0.24	0.48	-0.39	-0.23	-0.04	-0.85	-0.34	-0.26	0.09	0.32	-0.12	0.00	-0.73	-0.51	inf	1.83
12	BnaA09g42490D	AT2G22530	Alkaline-phosphatase-like family protein	-0.29	-0.12	-0.33	0.00	-0.47	-0.07	-0.37	0.02	0.32	-0.19	0.13	-0.10	0.62	-0.25	0.28	-0.01
13	BnaA10g00920D	AT1G02250	NAC domain containing protein 5 (NAC005)	0.00	inf	0.00	0.00	inf	inf	0.00	0.00	0.00	0.00	-1.14	0.00	0.30	inf	0.00	0.00
13	BnaA10g00930D	AT1G02270	Calcium-binding endonuclease/exonuclease/phosphatase family	0.67	0.68	0.46	0.31	0.93	0.57	0.28	0.70	-0.07	-0.19	-0.10	0.26	0.15	-0.06	0.22	-0.46
13	BnaA10g00940D	AT2G41610	unknown protein	-0.12	2.00	-1.77	0.39	-1.46	-0.51	-0.29	0.09	-0.06	-2.11	0.27	-0.81	-0.10	1.23	0.01	-0.16
13	BnaA10g00950D	AT1G02280	translocon at the outer envelope membrane of chloroplasts 33 (TOC33)	-0.03	1.74	0.51	-0.34	-0.04	0.19	0.47	0.42	-0.08	-0.48	-0.27	0.00	-0.06	0.19	-0.34	-0.22
13	BnaA10g00960D	AT4G36840	Galactose oxidase/kelch repeat superfamily protein	0.10	-0.75	0.83	0.62	-1.49	1.49	0.00	1.40	2.08	0.00	-1.06	inf	-1.34	inf	inf	inf
13	BnaA10g00970D	AT1G02310	Glycosyl hydrolase superfamily protein	0.40	-0.48	-4.32	1.19	-2.56	2.50	0.89	-1.23	-0.35	-0.35	0.14	1.99	1.76	0.00	0.63	-0.63
13	BnaA10g00980D	AT1G02330	CONTAINS InterPro DOMAIN/s: Hepatocellular carcinoma-associated antigen 59 (InterPro:IPR010756)	-0.16	-0.28	-0.20	-0.06	-0.39	-0.36	0.48	-0.18	0.40	-0.09	0.23	-0.57	-0.29	-0.22	-0.06	-0.32
13	BnaA10g00990D	AT1G02335	germin-like protein subfamily 2 member 2 precursor (GL22)	1.02	-0.62	-0.70	0.21	-1.32	-0.46	1.30	-0.60	-0.34	0.54	-0.32	0.14	-2.38	0.43	0.38	0.06
13	BnaA10g01000D	AT1G02340	LONG HYPOCOTYL IN FAR-RED (HFR1)	0.95	-1.83	0.00	-0.82	-1.34	-2.64	0.00	0.00	-0.22	1.54	-0.05	-0.06	0.08	-0.99	1.11	-0.40
13	BnaA10g01010D	AT4G01690	PPOX	0.24	0.69	-0.08	-0.62	0.89	0.29	-0.42	0.04	0.48	-0.35	-0.09	-0.05	-0.19	0.01	0.22	-0.36
13	BnaA10g01020D	AT1G02360	Chitinase family protein	1.65	-1.03	0.00	0.00	-0.64	1.87	0.00	inf	0.00	0.00	0.00	inf	0.37	-1.72	0.00	0.00
13	BnaA10g01030D	AT1G02360	Chitinase family protein	0.00	0.00	0.00	0.00	0.00	0.00	0.00	0.00	0.00	0.00	0.00	0.00	0.00	0.00	0.00	0.00
13	BnaA10g01040D	AT4G21240	F-box and associated interaction domains-containing protein	0.00	0.00	0.00	0.00	0.00	0.00	0.00	0.00	0.00	0.00	-1.48	0.00	0.00	0.00	0.00	0.00
13	BnaA10g01050D	AT2G46500	phosphoinositide 4-kinase gamma 4 (PI4K GAMMA 4)	0.00	0.00	0.00	0.00	0.00	0.00	0.00	0.00	0.00	0.00	0.00	0.00	inf	0.00	0.00	0.00
13	BnaA10g01060D	AT1G02370	Tetratricopeptide repeat (TPR)-like superfamily protein	-0.62	-0.99	0.02	-0.17	-0.57	-0.91	0.00	-0.33	0.07	0.18	0.15	-0.35	0.34	-0.07	-0.07	-0.21
13	BnaA10g01070D	AT1G02380	unknown protein	-0.83	-0.52	0.25	0.04	-0.14	-0.09	-0.06	0.21	0.38	0.21	-0.28	-0.51	0.17	-0.38	-0.04	-0.50
13	BnaA10g01080D			0.00	0.00	0.00	0.00	0.00	0.00	0.00	0.00	0.00	0.00	0.00	0.00	0.00	0.00	0.00	0.00
13	BnaA10g01090D	AT1G02390	glycerol-3-phosphate acyltransferase 2 (GPAT2)	-1.96	-1.29	-1.00	-1.17	0.13	0.84	-1.88	-1.93	0.36	-0.26	0.08	0.29	-0.29	0.39	0.83	-0.50
13	BnaA10g01100D	AT1G02400	gibberellin 2-oxidase 6 (GA2OX6)	-0.99	-3.09	0.00	0.75	-1.31	0.58	0.34	-1.12	-0.48	-2.27	-1.71	0.59	0.18	1.40	0.04	-0.17
13	BnaA10g01110D	AT1G02410	cytochrome c oxidase assembly protein CtaG / Cox11 family	0.00	0.00	-0.13	1.99	0.00	inf	inf	0.02	0.00	0.00	-0.07	0.84	0.36	0.00	0.62	0.00
13	BnaA10g01120D	AT1G45063	copper ion binding	0.00	0.00	0.21	0.00	0.00	0.00	0.00	2.98	0.00	0.00	1.13	-0.76	inf	0.00	0.00	0.00
13	BnaA10g01130D	AT1G02440	ADP-ribosylation factor D1A (ARFD1A)	0.00	0.00	0.00	0.00	0.00	0.00	0.00	0.00	0.00	0.00	0.00	0.00	0.00	0.00	0.00	0.00
13	BnaA10g01140D	AT1G02430	ADP-ribosylation factor D1B (ARFD1B)	0.00	0.00	0.00	0.00	0.00	0.00	0.00	0.00	0.00	0.00	0.00	0.00	0.00	0.00	0.00	0.00
13	BnaA10g01150D	AT1G02450	NIM1-interacting 1 (NIMIN1)	-1.59	-1.38	-2.39	0.00	-0.63	-0.74	0.00	-0.20	0.77	-1.23	-0.18	-1.65	-1.76	-0.27	inf	-1.82
13	BnaA10g01160D	AT1G02460	Pectin lyase-like superfamily protein	0.00	0.73	2.07	0.00	0.00	0.00	0.00	1.29	0.24	1.31	-1.00	-0.78	0.00	0.00	-0.85	0.00
13	BnaA10g01170D	AT1G02470	Polyketide cyclase/dehydrase and lipid transport superfamily protein	-0.34	-1.04	-1.01	0.44	-1.12	-0.49	0.33	-1.02	0.34	-1.16	0.95	0.79	-0.51	-1.05	-0.06	-0.17
13	BnaA10g01180D	AT1G02475	Polyketide cyclase/dehydrase and lipid transport superfamily protein	-0.39	-0.18	0.01	-0.05	-0.26	-0.22	-0.66	-0.24	0.27	0.33	0.41	-0.01	-0.46	0.25	0.34	0.15
13	BnaA10g01190D	AT1G02520	P-glycoprotein 11 (PGP11)	0.96	-0.68	1.34	-2.86	0.30	-0.10	-1.85	1.24	-0.25	-1.37	-0.23	-1.01	-0.64	-0.36	0.54	-0.48
13	BnaA10g01200D	AT1G02560	nuclear encoded CLP protease 5 (CLPP5)	0.24	0.72	0.25	-0.14	0.51	0.09	-0.37	0.32	0.26	0.17	0.19	0.19	0.04	0.51	0.13	-0.25
13	BnaA10g01210D	AT1G02570	unknown protein	0.00	0.00	0.00	0.00	0.00	0.00	inf	0.00	0.00	0.00	0.00	inf	0.00	0.00	0.00	0.00
13	BnaA10g01220D	AT1G02580	MEDEA (MEA)	0.00	0.00	0.00	0.00	0.00	0.00	0.00	0.00	0.00	0.00	inf	0.00	0.00	0.00	0.00	0.00

13	BnaA10g01230D	AT1G02610	RING/FYVE/PHD zinc finger superfamily protein	0.13	-3.06	1.38	0.22	-0.15	-0.39	0.86	-1.22	-0.07	0.69	-0.89	0.00	-0.54	-0.04	0.01	0.06
13	BnaA10g01240D	AT1G52730	Transducin/WD40 repeat-like superfamily protein	0.00	0.00	inf	0.00	0.00	0.00	0.00	0.00	0.00	0.00	0.00	0.00	inf	0.00	inf	0.00
13	BnaA10g01250D			0.00	inf	0.00	0.00	0.00	0.00	0.00	0.00	0.00	0.00	0.00	0.00	0.00	inf	0.00	0.00
13	BnaA10g01260D	AT1G02630	Nucleoside transporter family protein	0.00	0.00	0.00	0.00	0.00	0.00	0.00	0.00	0.00	0.00	0.00	0.00	0.00	0.00	0.00	0.00
13	BnaA10g01270D	AT1G02630	Nucleoside transporter family protein	0.00	0.00	0.00	0.00	0.00	0.00	0.00	0.00	0.00	inf	0.00	0.00	0.00	0.00	0.00	0.00
13	BnaA10g01280D	AT1G02640	beta-xylosidase 2 (BXL2)	0.71	0.49	-0.37	-0.39	-0.61	-0.91	0.11	-0.29	-0.68	0.23	0.44	0.39	1.36	1.15	1.73	-0.65
13	BnaA10g01290D	AT1G02680	TBP-associated factor 13 (TAF13)	0.01	-0.11	0.21	-0.47	0.24	-0.10	0.72	0.21	0.62	0.31	0.34	-0.08	0.17	-0.05	0.40	0.20
13	BnaA10g01300D	AT5G38700	unknown protein	2.35	0.00	inf	-1.93	4.26	4.81	-3.31	-2.13	0.41	0.00	-0.38	-0.37	-0.38	-0.38	0.00	0.00
13	BnaA10g01310D	AT5G61710	unknown protein	0.00	0.00	0.00	0.00	0.00	0.00	0.00	0.00	0.00	0.00	0.00	0.00	0.00	0.00	0.00	0.00
13	BnaA10g01320D			0.00	0.00	0.00	0.00	0.00	0.00	inf	0.00	0.00	inf	0.00	0.00	0.00	0.00	inf	0.00
13	BnaA10g01330D			0.00	0.00	0.00	0.00	0.00	0.00	0.00	0.00	0.00	0.00	0.00	0.00	0.00	0.00	0.00	0.00
13	BnaA10g01340D	AT1G02690	importin alpha isoform 6 (IMPA-6)	0.07	0.00	2.48	0.00	0.00	0.00	0.00	0.89	0.00	0.00	0.07	-0.67	0.00	0.00	0.00	-0.29
13	BnaA10g01350D	AT1G02700	unknown protein	0.00	-1.22	-0.14	1.17	-0.92	-1.60	1.42	0.39	-0.26	-0.29	-0.28	0.79	3.21	-0.70	0.04	1.09
13	BnaA10g01360D	AT1G02730	cellulose synthase-like D5 (CSLD5)	0.11	1.72	0.09	-3.44	-0.34	0.81	-2.45	-0.09	-0.35	-0.12	-0.20	-0.30	1.02	1.01	-0.50	0.39
13	BnaA10g01370D	AT1G02750	Drought-responsive family protein	1.41	0.95	1.12	0.79	2.10	1.81	1.20	1.81	0.45	-0.78	0.38	-0.68	-0.12	-1.41	-0.13	-0.77
13	BnaA10g01380D	AT3G16780	Ribosomal protein L19e family protein	-0.12	0.21	-0.19	0.08	0.26	-0.29	0.52	-0.24	-0.37	-0.06	-0.18	-0.17	-0.07	0.02	-0.01	-0.21
13	BnaA10g01390D	AT1G02790	polygalacturonase 4 (PGA4)	-1.12	-1.22	-1.19	-1.60	-0.29	-1.19	-1.36	-1.28	1.27	-0.37	0.31	1.38	4.12	1.10	5.22	inf
13	BnaA10g01400D	AT1G02810	Plant invertase/pectin methylesterase inhibitor superfamily	0.36	0.40	1.33	1.06	0.11	0.75	0.74	0.96	-0.43	-0.41	0.38	0.30	0.33	0.61	-0.11	0.73
13	BnaA10g01410D	AT1G02820	Late embryogenesis abundant 3 (LEA3) family protein	0.77	2.11	0.96	0.34	1.62	1.44	1.92	3.09	0.70	-1.09	-0.73	1.81	1.17	0.93	inf	1.28
13	BnaA10g01420D	AT3G05560	Ribosomal L22e protein family	-2.33	0.91	-0.63	0.26	-0.46	-2.17	0.82	-0.89	0.22	-0.17	0.35	-0.80	-0.72	-0.19	-0.87	0.42
13	BnaA10g01430D	AT1G02850	beta glucosidase 11 (BGLU11)	0.00	-1.48	-0.31	1.92	-0.64	-0.68	1.02	-0.39	0.00	0.00	0.84	0.26	1.17	-1.19	-0.71	0.35
13	BnaA10g01440D	AT1G02850	beta glucosidase 11 (BGLU11)	0.73	inf	1.51	-0.72	0.70	1.56	-0.21	0.34	0.00	0.00	0.37	-0.66	-2.11	-0.36	-0.34	-0.48
13	BnaA10g01450D	AT1G02860	nitrogen limitation adaptation (NLA)	0.24	inf	0.98	0.05	-1.38	-0.58	0.76	-1.37	-0.86	-0.25	-1.30	0.39	-0.95	0.47	-0.23	0.73
13	BnaA10g01460D	AT1G02870	FUNCTIONS IN: molecular_function unknown	0.53	0.75	0.42	0.70	0.48	0.19	0.46	0.25	-0.17	0.11	-0.01	0.05	0.44	0.34	0.43	0.56
13	BnaA10g01470D	AT4G02450	HSP20-like chaperones superfamily protein	-0.14	0.51	0.24	-0.29	0.28	0.51	-0.31	0.10	0.00	-0.31	0.01	-0.36	0.20	0.25	-0.40	0.23
13	BnaA10g01480D	AT1G02880	thiamin pyrophosphokinase1 (TPK1)	-0.52	-0.22	-0.45	-0.20	0.50	0.10	0.04	-0.04	0.62	0.22	-0.23	0.06	0.02	0.11	0.32	-0.64
13	BnaA10g01490D	AT1G02890	AAA-type ATPase family protein	0.12	-0.59	0.10	0.09	-0.01	-0.22	-0.18	-0.11	-0.14	0.09	0.27	-0.04	0.46	0.12	-0.16	0.23
13	BnaA10g01500D	AT1G02900	rapid alkalinization factor 1 (RALF1)	0.37	0.54	0.60	-0.30	0.27	inf	-1.00	1.36	-0.93	-0.72	-0.10	-0.21	-0.83	inf	0.11	-0.53
13	BnaA10g01510D	AT1G02930	glutathione S-transferase 6 (GSTF6)	-1.16	-1.54	-1.40	-1.07	1.45	-1.44	-1.25	-0.72	1.07	inf	-1.25	0.93	6.72	0.00	0.29	-0.25
13	BnaA10g01520D	AT1G02940	glutathione S-transferase (class phi) 5 (GSTF5)	0.00	0.00	0.00	0.00	0.00	0.00	0.00	0.00	0.00	0.00	0.00	0.00	0.00	0.00	inf	inf
13	BnaA10g01530D	AT1G02940	glutathione S-transferase (class phi) 5 (GSTF5)	0.00	0.00	0.00	0.00	0.00	0.00	0.00	0.00	0.00	0.00	0.00	0.00	0.00	0.00	0.00	0.00
13	BnaA10g01540D	AT1G02940	glutathione S-transferase (class phi) 5 (GSTF5)	0.00	0.00	0.00	0.00	0.00	0.00	0.00	0.00	0.00	0.00	0.00	0.00	0.00	0.00	inf	0.00
14	BnaA10g20290D	AT5G12410	THUMP domain-containing protein	-0.48	-0.68	-0.24	0.10	-0.32	-0.32	0.12	-0.26	0.18	-0.11	0.18	-0.35	-0.34	-0.67	-0.06	-0.07
14	BnaA10g20300D	AT5G12400	DNA binding	-4.73	-6.28	-0.30	0.39	-8.95	-7.73	0.16	0.41	0.04	1.19	1.60	0.61	5.57	-4.80	-0.03	-0.38
14	BnaA10g20310D	AT5G12390	FISSION 1B (FIS1B)	-0.10	-0.50	-0.03	-0.35	0.58	-0.42	-0.12	0.34	0.37	-0.37	0.35	0.02	-0.42	-0.99	0.11	-0.25
14	BnaA10g20320D	AT5G12380	annexin 8 (ANNAT8)	-1.47	inf	0.97	-2.58	-0.98	inf	-0.41	0.86	inf	1.06	1.45	-0.14	-0.76	-0.21	-1.10	0.92
14	BnaA10g20330D	AT5G12370	exocyst complex component sec10 (SEC10)	0.36	-0.43	-0.18	-0.03	-0.44	-0.01	-0.44	-0.16	0.06	-0.23	0.17	0.00	0.17	0.09	0.06	-0.20
14	BnaA10g20340D	AT5G12360	unknown protein	0.06	0.00	-0.17	0.00	inf	0.00	0.00	0.48	1.42	0.05	-0.90	-0.32	0.00	0.00	inf	-0.66
14	BnaA10g20350D	AT5G12350	Regulator of chromosome condensation (RCC1) family with FYVE zinc finger domain	0.17	-0.05	0.24	-0.10	0.16	0.22	-0.27	0.01	0.09	-0.27	-0.19	0.23	-0.45	0.05	-0.27	-0.20
14	BnaA10g20360D	AT5G12340	unknown protein	0.00	0.00	0.00	0.00	0.00	0.00	0.00	0.00	0.00	0.00	0.00	0.00	0.00	0.00	0.00	0.00
14	BnaA10g20370D	AT5G12330	LATERAL ROOT PRIMORDIUM 1 (LRP1)	-0.04	-1.26	0.08	-1.75	0.08	inf	1.06	0.77	-0.53	-1.09	-0.16	0.45	-0.80	inf	-0.89	inf
14	BnaA10g20380D	AT5G12320	ankyrin repeat family protein	0.10	-0.79	-0.13	-0.11	0.28	-0.56	1.01	0.56	0.16	0.08	0.05	-0.52	0.51	-0.56	-0.49	0.54
14	BnaA10g20390D	AT5G12310	RING/U-box superfamily protein	-1.52	-1.70	-0.13	0.22	-0.63	-0.85	-0.02	-0.42	1.11	-0.48	0.23	-0.43	0.25	0.48	0.15	0.37
14	BnaA10g20400D	AT5G12300	Calcium-dependent lipid-binding (CaLB domain) family protein	-0.54	0.22	-0.51	-1.31	-1.36	-1.21	inf	-0.27	-0.03	-0.35	-0.95	-0.26	0.28	0.28	-1.06	-0.94
14	BnaA10g20410D	AT5G12260	BEST Arabidopsis thaliana protein match is: glycosyltransferase family protein 2 (TAIR:AT5G60700.1)	0.45	0.06	-0.12	0.40	-0.22	0.25	-0.62	-0.02	0.00	0.29	-0.32	0.28	0.43	0.11	0.23	-0.47
14	BnaA10g20420D	AT5G12250	beta-6 tubulin (TUB6)	-0.04	0.32	-0.31	-0.74	-0.17	-0.39	-0.88	-0.12	0.17	-0.05	-0.25	0.03	-0.37	0.61	-0.08	-0.34
14	BnaA10g20430D	AT5G12240	unknown protein	-0.34	0.06	0.55	0.19	0.29	-0.32	0.19	0.51	-1.29	-0.06	-1.41	0.12	0.00	-0.45	-0.53	-0.93

14	BnaA10g20440D	AT5G12235	CLAVATA3/ESR-RELATED 22 (CLE22)	1.35	inf	inf	0.00	1.39	0.00	0.00	inf	-1.43	0.00	inf	0.00	0.00	0.00	0.00	0.00
14	BnaA10g20450D	AT5G12230	MED19A	-0.19	-0.40	0.73	-0.31	-0.25	-0.12	0.30	-0.28	0.21	-0.71	0.02	-0.40	-0.21	-0.27	-0.49	-0.23
14	BnaA10g20460D	AT5G12210	RAB geranylgeranyl transferase beta subunit 1	0.33	0.19	0.21	-0.47	0.60	0.10	-0.24	0.62	-1.06	0.18	-0.12	0.21	-0.38	-0.32	-0.38	-0.13
14	BnaA10g20470D	AT5G12200	pyrimidine 2 (PYD2)	-0.08	-0.17	-0.41	-0.49	0.16	0.09	-0.19	-0.48	-0.45	0.00	0.03	-0.07	-0.09	-0.16	-0.34	-0.46
14	BnaA10g20480D	AT5G12190	RNA-binding (RRM/RBD/RNP motifs) family protein	-0.10	0.61	0.52	-0.29	0.53	0.08	0.58	0.20	-0.69	-0.10	-0.09	0.20	0.53	0.20	-0.07	0.40
14	BnaA10g20490D	AT5G12180	calcium-dependent protein kinase 17 (CPK17)	0.11	-1.12	-1.42	-0.78	2.55	-0.10	0.50	-3.74	0.00	0.00	-0.63	inf	inf	1.31	0.00	0.00
14	BnaA10g20500D	AT5G12150	Rho GTPase activation protein (RhoGAP) with PH domain	0.23	0.44	0.17	0.26	0.11	-0.34	0.29	-0.16	-0.28	0.16	-0.01	-0.22	-0.13	0.15	-0.06	-0.24
14	BnaA10g20510D			0.00	0.00	0.00	0.00	0.00	0.00	0.00	0.00	0.00	0.00	0.00	0.00	0.00	0.00	0.00	0.00
14	BnaA10g20520D			0.00	0.00	0.00	0.00	0.00	0.00	0.00	0.00	0.00	0.00	0.00	0.00	0.00	0.00	0.00	0.00
14	BnaA10g20530D	AT5G12140	cystatin-1 (CYS1)	-0.16	0.08	-0.17	0.15	0.35	0.34	-0.20	-0.02	-0.25	-0.25	-0.17	0.20	0.00	-0.35	0.01	-0.45
14	BnaA10g20540D	AT5G12130	PIGMENT DEFECTIVE 149 (PDE149)	0.14	1.02	0.29	0.15	0.45	0.02	-0.13	0.36	0.19	0.07	0.22	0.09	-0.18	-0.26	0.02	-0.26
15	BnaC01g22420D	AT5G56500	TCP-1/cpn60 chaperonin family protein	-0.19	0.85	-0.14	-0.27	0.86	0.67	0.72	0.39	0.02	0.98	1.06	1.22	-1.14	0.21	-3.20	1.12
15	BnaC01g22430D	AT4G15480	UGT84A1	inf	0.09	0.00	0.00	0.00	0.00	0.00	0.00	0.00	inf	0.00	0.00	0.00	0.00	inf	-0.21
15	BnaC01g22440D			0.00	0.00	0.00	0.00	0.00	0.00	0.00	0.00	0.00	0.00	0.00	0.00	0.00	0.00	0.00	0.00
15	BnaC01g22450D	AT4G15450	Senescence/dehydration-associated protein-related	-0.03	0.65	-0.32	0.29	-0.42	-0.46	0.18	-0.21	-3.14	2.49	-0.05	1.58	-0.79	0.88	-0.94	-0.03
15	BnaC01g22460D	AT1G63400	Pentatricopeptide repeat (PPR) superfamily protein	1.22	0.39	-0.18	0.22	2.06	1.65	-2.00	-0.97	inf	inf	0.00	inf	0.00	0.81	inf	0.57
15	BnaC01g22470D	AT1G62670	rna processing factor 2 (RPF2)	0.05	1.89	0.61	1.08	-0.37	0.06	1.47	-0.48	0.62	inf	1.07	0.34	-1.53	inf	-0.17	0.89
15	BnaC01g22480D	AT4G15430	ERD (early-responsive to dehydration stress) family protein	-1.30	-1.09	-0.56	1.67	0.18	1.80	inf	-0.74	-0.62	-3.72	-0.01	-0.02	0.81	1.44	-0.27	-0.16
15	BnaC01g22490D	AT4G15420	Ubiquitin fusion degradation UFD1 family protein	0.00	0.00	0.00	0.00	0.00	0.00	0.00	0.00	0.00	0.00	0.00	0.00	0.00	0.00	0.00	0.00
15	BnaC01g22500D	AT4G15160	Bifunctional inhibitor/lipid-transfer protein/seed storage 2S albumin superfamily protein	0.29	0.40	-0.02	1.98	0.16	-0.57	0.69	-0.60	-1.85	-1.59	-0.31	0.62	1.49	0.60	0.33	2.38
15	BnaC01g22510D	AT4G15130	phosphorylcholine cytidylyltransferase2 (CCT2)	-0.08	-0.64	0.71	-0.89	0.32	-1.46	0.00	1.43	-0.74	0.73	-0.92	0.62	0.28	-1.59	inf	0.20
15	BnaC01g22520D	AT4G15120	VQ motif-containing protein	-1.04	1.40	-0.95	0.90	-0.60	0.74	-0.19	0.10	inf	0.00	-1.47	1.25	0.58	inf	0.44	0.72
15	BnaC01g22530D	AT4G15080	DHHC-type zinc finger family protein	0.17	0.05	-0.22	-0.19	-0.02	0.01	0.03	0.27	0.24	-0.14	-0.25	0.04	-0.11	-0.31	-0.11	0.13
15	BnaC01g22540D	AT4G00315	F-box/RNI-like/FBD-like domains-containing protein	0.00	0.11	0.60	0.00	0.00	inf	0.00	1.47	0.00	0.00	-0.10	inf	0.00	0.00	0.64	0.00
15	BnaC01g22550D	AT2G07240	cysteine-type peptidases	0.00	0.00	0.00	0.00	0.00	0.00	0.00	0.00	0.00	0.00	inf	0.00	0.00	0.00	0.00	inf
15	BnaC01g22560D	AT1G10190	Protein of Unknown Function (DUF239)	0.00	0.00	0.00	-2.38	-0.66	-1.98	-1.99	0.00	0.00	0.00	inf	1.66	2.86	0.00	-1.83	0.28
15	BnaC01g22570D	AT4G15040	Subtilisin-like serine endopeptidase family protein	0.00	inf	0.00	-0.78	0.00	inf	0.00	-0.90	1.20	inf	-0.47	0.46	0.00	0.00	inf	-0.43
15	BnaC01g22580D	AT4G15030	FUNCTIONS IN: molecular_function unknown	-0.19	0.43	0.04	0.21	0.40	0.30	0.81	0.11	0.19	0.00	-0.12	-0.62	0.05	-0.09	0.58	-0.56
15	BnaC01g22590D	AT4G15020	hAT transposon superfamily	0.18	0.03	-0.19	-0.28	-0.04	-0.09	-0.15	-0.01	-0.33	-0.16	-0.33	0.02	0.26	-0.15	-0.16	-0.16
15	BnaC01g22600D	AT4G15020	hAT transposon superfamily	0.52	0.04	-0.01	-0.32	-0.47	0.68	-0.76	0.05	-0.32	-0.49	0.39	0.13	-0.05	-0.43	-0.20	-0.13
15	BnaC01g22610D	AT4G15010	Mitochondrial substrate carrier family protein	-0.31	-0.14	-0.27	0.00	0.43	-0.92	0.18	-0.03	0.05	-0.13	0.34	0.00	-0.12	-0.10	0.20	-0.43
15	BnaC01g22620D	AT4G15000	Ribosomal L27e protein family	-1.97	-0.95	-0.25	-0.33	-1.26	0.36	0.47	-0.13	-1.07	1.48	-1.07	-0.92	-0.32	-0.04	-0.32	0.00
15	BnaC01g22630D	AT4G14990	Topoisomerase II-associated protein PAT1	-0.04	-0.46	-0.19	0.15	-0.02	-0.10	-0.54	-0.08	0.25	0.16	0.01	0.62	0.64	-0.30	0.01	-0.15
15	BnaC01g22640D	AT4G14950	SNARE associated Golgi protein family	0.06	-0.19	0.03	-0.14	0.22	0.38	0.20	0.18	0.05	0.23	0.49	-0.18	0.09	-0.31	-0.12	-0.36
15	BnaC01g22650D	AT4G14940	amine oxidase 1 (AO1)	0.18	1.48	inf	0.00	-0.29	-0.54	0.00	-1.08	-0.35	-0.18	0.79	-0.92	0.25	-0.04	0.00	0.00
15	BnaC01g22660D	AT4G14930	Survival protein SurE-like phosphatase/nucleotidase	0.00	0.00	0.00	0.00	0.00	0.00	0.00	0.00	0.00	0.00	0.00	0.00	0.00	0.00	0.00	0.00
15	BnaC01g22670D	AT2G45550	cytochrome P450, family 76, subfamily C, polypeptide 4 (CYP76C4)	0.00	0.00	0.00	0.00	0.00	0.00	0.00	0.00	0.00	0.00	0.00	0.00	0.00	0.00	0.00	inf
15	BnaC01g22680D	AT2G03750	P-loop containing nucleoside triphosphate hydrolases superfamily protein	0.00	0.13	inf	0.29	-1.55	-2.02	0.70	-0.36	0.00	0.00	inf	inf	6.15	0.00	-0.63	-0.28
15	BnaC01g22690D	AT4G14920	Acyl-CoA N-acyltransferase with RING/FYVE/PHD-type zinc finger protein	0.01	-0.38	0.22	-0.26	-0.04	0.30	0.04	0.04	-0.41	0.14	-0.05	-0.21	0.07	-0.19	-0.08	0.05
15	BnaC01g22700D	AT4G14890	2Fe-2S ferredoxin-like superfamily protein	0.07	0.48	-0.03	-0.86	0.15	-0.23	-1.17	0.20	0.18	-0.21	-0.12	0.22	-0.42	-0.01	0.34	0.14
15	BnaC01g22710D	AT4G14880	O-acetylserine (thiol) lyase (OAS-TL) isoform A1 (OASA1)	0.36	0.18	-0.19	0.53	0.16	-0.03	0.80	-0.06	-0.20	-0.13	0.13	0.17	-0.11	-0.21	0.15	-0.18
15	BnaC01g22720D	AT4G14860	ovate family protein 11 (OFP11)	0.00	0.00	inf	0.00	-1.22	inf	0.00	inf	0.00	inf	1.28	0.00	inf	-2.06	0.00	0.00
15	BnaC01g22730D	AT4G14820	Pentatricopeptide repeat (PPR) superfamily protein	0.01	-0.69	-0.10	0.35	-0.07	-0.26	0.60	-0.36	0.56	-0.68	-0.38	-0.12	0.07	0.02	0.08	-0.36
15	BnaC01g22740D	AT4G14819	Protein of unknown function (DUF1677)	0.00	0.00	0.00	0.00	0.00	0.00	0.00	0.00	0.00	0.00	-1.29	0.00	0.00	0.00	0.00	0.00

15	BnaC01g22750D	AT3G22630	20S proteasome beta subunit D1 (PBD1)	-0.13	-0.16	-0.03	-0.22	0.37	0.13	-0.34	0.05	0.55	-0.16	-0.18	0.23	0.70	0.05	0.50	0.14
15	BnaC01g22760D	AT4G14790	ATSUV3	0.12	0.74	0.09	0.35	0.53	0.34	0.24	-0.13	-0.13	-1.03	0.29	-0.37	0.27	-0.55	-0.17	0.03
15	BnaC01g22770D	AT5G65850	F-box and associated interaction domains-containing protein	0.00	0.00	0.00	0.00	0.00	0.00	0.00	0.00	0.00	0.00	0.00	0.00	0.00	0.00	0.00	0.00
15	BnaC01g22780D	AT2G31470	DROUGHT TOLERANCE REPRESSOR (DOR)	-0.43	-1.23	-0.46	-0.69	0.25	0.35	1.17	-0.51	-0.80	-0.08	-1.23	1.15	-0.88	-0.17	-0.58	-0.78
15	BnaC01g22790D	AT5G65850	F-box and associated interaction domains-containing protein	-0.66	-0.55	-0.07	1.23	1.11	-0.56	2.73	-0.59	0.67	0.34	-0.83	0.46	-0.73	0.80	-0.03	-0.45
15	BnaC01g22800D			inf	0.00	inf	inf	0.00	inf	0.14	0.00	0.00	0.00	0.00	0.00	0.00	0.00	0.00	inf
15	BnaC01g22810D	AT4G14770	TESMIN/TSO1-like CXC 2 (TCX2)	0.15	-0.07	-0.39	-1.98	-0.73	0.43	-1.87	0.26	-0.06	-0.86	0.11	-0.38	2.32	-0.30	-0.37	-0.42
15	BnaC01g22820D	AT4G14760	kinase interacting (KIP1-like) family protein	0.48	-0.75	0.13	-0.40	0.01	0.30	-0.84	-0.05	0.06	0.33	0.23	-0.12	-0.19	-0.02	0.18	-0.16
16	BnaC04g41700D	AT2G31010	Protein kinase superfamily protein	-0.16	0.26	0.12	-1.57	1.23	0.03	-0.32	-0.07	0.19	-0.61	0.23	-0.30	-1.44	1.29	-0.79	0.83
16	BnaC04g41710D	AT2G31040	ATP synthase protein I -related	-0.14	0.43	0.15	0.16	0.26	-0.17	0.23	0.28	0.46	-0.01	-0.15	0.28	-0.89	-0.25	0.00	-0.14
16	BnaC04g41720D			0.00	0.00	0.00	0.00	0.00	0.00	0.00	0.00	0.00	0.00	inf	0.00	0.00	0.00	0.00	0.00
16	BnaC04g41730D	AT3G29740	BTB/POZ domain-containing protein	0.00	0.00	0.00	0.00	0.00	0.00	0.00	0.00	0.00	0.00	0.00	0.00	0.00	0.00	0.00	0.00
16	BnaC04g41740D	AT2G40450	BTB/POZ domain-containing protein	1.32	-1.39	-0.93	inf	0.00	0.00	0.00	0.05	-0.57	0.18	0.65	-0.33	0.00	-1.47	0.00	inf
16	BnaC04g41750D			0.94	-3.26	0.84	0.60	2.01	0.82	0.52	-0.30	-3.05	1.88	-1.34	-1.97	0.33	1.55	0.99	1.16
16	BnaC04g41760D	AT3G13280	Putative endonuclease or glycosyl hydrolase	0.11	0.11	0.13	-0.04	0.20	0.21	-0.13	0.15	0.12	0.15	0.03	0.35	-0.03	0.30	0.02	0.10
16	BnaC04g41770D	AT5G39440	SNF1-related protein kinase 1.3 (SnRK1.3)	0.00	0.00	0.00	0.00	0.00	0.00	0.00	0.00	0.00	0.00	0.00	0.00	0.00	0.00	0.00	1.90
16	BnaC04g41780D	AT1G09780	Phosphoglycerate mutase, 2,3-bisphosphoglycerate-independent	0.00	0.00	0.00	0.00	0.00	0.00	0.00	0.00	0.00	0.00	0.00	0.00	0.00	0.00	0.00	0.00
16	BnaC04g41790D	AT5G39430	Protein of unknown function (DUF1336)	0.00	0.00	0.05	0.00	0.00	0.00	0.00	-1.05	0.00	0.00	0.79	-0.40	0.00	0.00	0.00	0.00
16	BnaC04g41800D	AT2G31082	CLAVATA3/ESR-RELATED 7 (CLE7)	0.00	0.00	0.00	0.00	0.00	0.00	0.00	0.00	0.00	0.00	0.00	0.00	0.00	0.00	0.00	0.00
16	BnaC04g41810D	AT4G09490	Polynucleotidyl transferase, ribonuclease H-like superfamily protein	0.00	0.00	0.00	0.00	0.00	0.00	0.00	0.00	0.00	0.00	0.00	0.00	0.00	0.00	0.00	0.00
16	BnaC04g41820D	AT2G31085	CLAVATA3/ESR-RELATED 6 (CLE6)	0.00	0.00	0.00	0.00	0.00	0.00	0.00	0.00	0.00	0.00	0.00	0.00	0.00	0.00	0.00	0.00
16	BnaC04g41830D			inf	0.00	0.57	-1.39	0.00	0.00	-3.02	0.95	0.00	0.00	-0.03	-0.67	1.85	0.00	-0.51	0.61
16	BnaC04g41840D			0.00	0.00	0.00	0.00	0.00	0.00	0.00	0.00	0.00	0.00	0.00	0.00	0.00	0.00	0.00	0.00
16	BnaC04g41850D	AT2G31090	unknown protein	1.31	-0.46	-0.82	0.56	-0.29	0.87	0.41	-3.16	2.93	0.13	-0.77	0.14	1.07	0.29	0.23	0.84
16	BnaC04g41860D			0.00	0.00	0.00	0.00	0.00	0.00	0.00	0.00	0.00	0.00	0.00	0.00	0.00	0.00	0.00	0.00
16	BnaC04g41870D	AT1G76490	hydroxy methylglutaryl CoA reductase 1 (HMG1)	0.00	0.00	0.00	0.00	0.00	0.00	0.00	0.00	0.00	0.00	0.00	0.00	0.00	0.00	0.00	0.00
16	BnaC04g41880D			0.00	0.00	0.00	0.00	0.00	0.00	0.00	0.00	0.00	0.00	0.00	0.00	0.00	0.00	0.00	0.00
16	BnaC04g41890D	AT5G08640	flavonol synthase 1 (FLS1)	0.00	0.00	0.00	0.00	0.00	0.00	0.00	0.00	0.00	0.00	inf	inf	0.00	0.00	0.00	0.00
16	BnaC04g41900D	AT2G31110	INVOLVED IN: biological_process unknown	-1.68	-1.41	-0.62	-0.79	0.00	0.56	0.00	0.09	-3.63	-0.15	inf	0.00	0.00	0.00	-0.02	inf
16	BnaC04g41910D	AT2G38770	EMBRYO DEFECTIVE 2765 (EMB2765)	0.00	0.00	0.00	0.00	0.00	0.00	inf	0.00	0.00	0.00	0.00	0.00	0.00	0.00	0.00	0.00
16	BnaC04g41920D	AT2G31130	unknown protein	0.00	-0.06	0.14	0.56	0.12	0.35	0.56	0.04	-0.23	-0.21	-0.47	-0.52	0.39	-0.92	-0.30	-0.26
16	BnaC04g41930D	AT2G31050	Cupredoxin superfamily protein	0.00	0.00	inf	0.00	0.00	0.00	0.00	inf	0.00	0.00	-0.77	inf	0.00	0.00	inf	0.00
16	BnaC04g41940D	AT4G25780	CAP (Cysteine-rich secretory proteins, Antigen 5, and Pathogenesis-related 1 protein) superfamily protein	0.00	0.00	0.00	0.00	0.00	0.00	0.00	0.00	0.00	0.00	0.00	0.00	0.00	0.00	0.00	0.00
16	BnaC04g41950D	AT2G31050	Cupredoxin superfamily protein	0.00	0.00	0.00	0.00	0.00	0.00	0.00	0.00	0.00	0.00	0.00	0.00	0.00	0.00	0.00	0.00
16	BnaC04g41960D			0.00	0.00	0.00	0.00	0.00	0.00	0.00	0.00	0.00	0.00	0.00	0.00	0.00	0.00	0.00	0.00
16	BnaC04g41970D	AT2G31160	LIGHT SENSITIVE HYPOCOTYLS 3 (LSH3)	-1.28	0.00	0.00	0.00	inf	-1.38	inf	-1.56	-1.13	0.00	-1.24	1.08	2.34	-1.11	2.12	-3.20
16	BnaC04g41980D	AT2G31180	myb domain protein 14 (MYB14)	0.00	0.00	0.00	0.00	0.00	0.00	0.00	0.00	0.00	0.00	0.00	0.00	0.00	0.00	0.00	0.00
16	BnaC04g41990D	AT2G31180	myb domain protein 14 (MYB14)	-1.70	0.00	0.00	0.00	-0.73	0.00	0.00	-0.70	0.92	0.00	0.00	2.05	-0.68	1.51	0.00	inf
16	BnaC04g42000D	AT2G31190	ROOT UV-B SENSITIVE 2 (RUS2)	0.08	0.29	0.44	-0.10	0.05	-0.27	0.69	-0.02	-0.35	0.25	-0.24	-0.08	0.04	-0.59	0.19	-0.31
16	BnaC04g42010D	AT5G02500	heat shock cognate protein 70-1 (HSC70-1)	0.11	-0.19	-0.15	-0.21	-0.25	0.26	-0.41	-0.09	0.13	0.35	-0.31	0.25	0.10	0.52	-0.17	0.18
16	BnaC04g42020D	AT5G02500	heat shock cognate protein 70-1 (HSC70-1)	0.29	-0.32	-0.09	0.11	-0.04	-0.03	-0.26	-0.19	0.46	0.45	-0.42	0.42	0.15	0.14	-0.19	0.08
16	BnaC04g42030D	AT2G31210	basic helix-loop-helix (bHLH) DNA-binding superfamily protein	inf	-2.11	0.84	-0.40	inf	1.07	-0.95	0.83	inf	0.00	0.58	-0.37	0.00	0.00	inf	inf
16	BnaC04g42040D	AT2G31210	basic helix-loop-helix (bHLH) DNA-binding superfamily protein	0.00	0.00	0.00	0.00	0.00	0.00	0.00	0.00	0.00	0.00	0.00	0.00	0.00	0.00	0.00	0.00
16	BnaC04g42050D	AT2G31220	basic helix-loop-helix (bHLH) DNA-binding superfamily protein	0.49	0.40	1.22	0.00	-2.20	-0.70	-2.64	0.98	-2.56	0.00	0.70	-0.53	2.88	0.00	1.27	inf

16	BnaC04g42060D	AT2G31230	ethylene-responsive element binding factor 15 (ERF15)	-0.52	-1.33	0.07	0.00	0.57	-0.10	0.00	inf	-0.47	0.43	inf	inf	-0.62	-0.92	0.00	0.00
16	BnaC04g42070D			0.00	0.00	0.00	0.00	0.00	0.00	0.00	0.00	0.00	0.00	0.00	0.00	0.00	0.00	0.00	0.00
16	BnaC04g42080D	AT5G15690	zinc ion binding	0.00	0.00	0.00	0.00	0.00	0.00	0.00	0.00	0.00	0.00	0.00	0.00	0.00	0.00	0.00	0.00
16	BnaC04g42090D	AT2G07200	Cysteine proteinases superfamily protein	0.00	0.00	0.00	0.00	0.00	0.00	0.00	0.00	0.00	0.00	0.00	0.00	0.00	0.00	0.00	0.00
16	BnaC04g42100D	AT2G07190	CONTAINS InterPro DOMAIN/s: Domain of unknown function DUF1985 (InterPro:IPR015410)	0.00	0.00	0.00	0.00	0.00	0.00	0.00	0.00	0.00	inf	0.00	0.00	0.00	0.00	-0.18	0.00
16	BnaC04g42110D			0.00	0.00	0.00	0.00	0.00	0.00	0.00	0.00	0.00	0.00	0.00	0.00	0.00	0.00	0.00	0.00
16	BnaC04g42120D	AT2G31240	Tetratricopeptide repeat (TPR)-like superfamily protein	-0.29	-0.45	0.01	0.87	-0.72	-0.02	0.34	-0.19	0.83	0.01	-0.36	-0.06	-0.40	-0.27	-0.56	0.59
16	BnaC04g42130D	AT2G31260	autophagy 9 (APG9)	0.46	-0.44	0.35	0.29	0.02	0.48	0.08	0.05	-0.02	0.08	0.39	-0.05	0.40	-0.23	0.02	0.01
16	BnaC04g42140D	AT2G31270	homolog of yeast CDT1 A (CDT1A)	-0.18	-0.64	0.11	0.02	0.03	-0.32	-0.11	0.12	0.08	-0.61	-0.32	-0.39	1.53	-0.33	0.04	0.10
16	BnaC04g42150D			0.00	0.00	0.00	0.00	0.00	0.00	0.00	0.00	0.00	0.00	inf	0.00	0.00	inf	0.00	0.00
16	BnaC04g42160D	AT1G06149	conserved peptide upstream open reading frame 8 (CPuORF8)	0.00	0.00	0.00	0.00	0.00	0.00	0.00	0.00	0.00	0.00	0.00	0.00	0.00	0.00	0.00	0.00
16	BnaC04g42170D	AT2G31280	conserved peptide upstream open reading frame 7 (CPUORF7)	-0.34	0.11	0.06	-0.16	0.05	0.07	-0.09	0.12	-0.27	-0.15	-0.12	0.01	0.21	0.21	-0.11	-0.25
16	BnaC04g42180D	AT2G31305	INHIBITOR-3 (INH3)	0.34	-0.33	0.32	0.40	0.33	1.10	0.10	-0.87	-0.45	-1.07	0.24	0.26	-0.22	-0.86	0.31	0.33
16	BnaC04g42190D	AT2G31720	FUNCTIONS IN: DNA binding	2.88	0.00	2.58	0.00	0.04	inf	0.00	2.49	0.00	0.00	1.21	0.27	0.00	0.00	0.00	inf
16	BnaC04g42200D	AT2G31720	FUNCTIONS IN: DNA binding	0.00	0.00	2.19	0.00	0.00	inf	0.00	2.51	0.00	0.00	0.49	-0.67	0.00	inf	0.00	0.00
16	BnaC04g42210D	AT2G31865	poly(ADP-ribose) glycohydrolase 2 (PARG2)	0.00	0.00	inf	0.00	0.00	0.00	0.00	inf	0.00	0.00	0.00	0.00	0.00	0.00	0.00	0.00
16	BnaC04g42220D	AT2G31865	poly(ADP-ribose) glycohydrolase 2 (PARG2)	0.00	0.00	0.00	0.00	0.00	0.00	0.00	0.00	0.00	0.00	0.00	0.00	0.00	0.00	0.00	0.00
16	BnaC04g42230D	AT2G31710	Vacuolar ATPase assembly integral membrane protein VMA21-like domain	-0.75	0.00	inf	0.32	1.38	0.78	-0.99	0.00	0.00	inf	2.65	0.00	0.00	1.92	inf	1.12
16	BnaC04g42240D	AT2G31660	SUPER SENSITIVE TO ABA AND DROUGHT2 (SAD2)	-0.20	0.43	-0.10	0.57	-0.10	-0.38	0.15	-0.17	0.41	-0.83	-0.08	0.12	-0.16	0.19	0.20	-0.05
16	BnaC04g42250D	AT2G31650	homologue of trithorax (ATX1)	-0.20	0.34	0.09	-0.52	-0.17	0.73	-0.48	-0.44	-0.31	-0.39	0.12	-0.17	0.50	0.04	0.35	-0.24
16	BnaC04g42260D	AT5G35530	Ribosomal protein S3 family protein	-0.56	0.44	-0.11	0.08	0.23	0.12	0.37	-0.02	0.13	-0.75	-0.32	-0.44	-0.10	-0.60	-0.14	-0.41
17	BnaC06g10070D	AT3G15270	squamosa promoter binding protein-like 5 (SPL5)	3.45	0.49	0.00	inf	0.50	0.34	0.00	1.45	0.01	1.61	inf	inf	0.62	-0.88	inf	0.00
17	BnaC06g10080D	AT1G53140	Dynamin related protein 5A (DRP5A)	0.09	-0.02	0.33	0.53	0.00	-0.23	0.07	0.22	-0.84	-0.16	0.45	0.09	0.83	0.30	-0.32	0.03
17	BnaC06g10090D	AT1G53120	RNA-binding S4 domain-containing protein	-0.25	0.25	0.29	-0.36	0.04	0.16	0.51	0.01	0.92	-0.15	0.28	-0.18	-0.30	-0.19	-0.52	-0.49
17	BnaC06g10100D	AT1G53090	SPA1-related 4 (SPA4)	-0.11	-0.50	-0.62	0.19	-0.11	-0.19	-0.67	-0.21	0.91	-0.28	-0.24	-0.11	-0.13	0.55	0.37	0.33
17	BnaC06g10110D	AT1G53050	Protein kinase superfamily protein	0.37	0.17	0.08	-0.70	0.50	0.25	-0.62	0.02	-0.07	-0.12	-0.09	-0.01	0.40	0.27	0.17	0.21
17	BnaC06g10120D			0.00	0.00	0.00	0.00	0.00	0.00	0.00	0.00	0.00	0.00	0.00	0.00	0.00	0.00	0.00	0.00
17	BnaC06g10130D	AT1G53590	NTMC2T6.1	-0.25	-0.58	0.00	0.07	-0.06	-0.23	0.18	-0.38	0.61	-0.10	-0.11	-0.35	0.15	-0.04	-0.04	-0.03
17	BnaC06g10140D	AT1G54445	LOCATED IN: endomembrane system	0.00	0.00	0.00	-1.09	0.00	0.00	0.14	0.00	0.00	0.00	0.00	inf	0.00	0.00	-0.88	0.95
17	BnaC06g10150D			0.00	0.00	inf	0.00	0.00	0.00	0.00	inf	0.00	0.00	0.00	0.00	0.00	0.00	0.00	0.00
17	BnaC06g10160D			0.00	0.00	0.00	0.00	0.00	0.00	0.00	0.00	0.00	0.00	0.00	0.00	0.00	0.00	0.00	0.00
17	BnaC06g10170D	AT1G53635	unknown protein	0.00	0.00	0.00	0.00	inf	0.00	0.00	0.00	0.00	0.00	0.00	0.00	0.00	0.00	0.00	0.00
17	BnaC06g10180D	AT4G27040	VPS22	0.00	0.00	0.00	0.00	0.00	0.00	0.00	0.00	0.00	0.00	0.00	0.00	0.00	0.00	0.00	0.00
17	BnaC06g10190D			0.00	0.00	0.00	0.00	0.00	0.00	0.00	0.00	0.00	0.00	0.00	0.00	0.00	0.00	0.00	0.00
17	BnaC06g10200D			0.00	0.00	0.00	0.00	0.00	0.00	0.00	0.00	0.00	0.00	0.00	0.00	0.00	0.00	0.00	0.00
17	BnaC06g10210D			0.00	0.00	0.00	0.00	0.00	0.00	0.00	0.00	0.00	0.00	0.00	0.00	0.00	0.00	0.00	0.00
17	BnaC06g10220D			0.00	0.00	0.00	0.00	0.00	0.00	0.00	0.00	0.00	0.00	0.00	0.00	0.00	0.00	0.00	0.00
17	BnaC06g10230D	AT1G53700	WAG 1 (WAG1)	-1.32	-0.65	1.36	-1.32	0.00	0.00	-1.22	0.37	-1.30	-0.54	-0.27	-0.25	2.56	2.04	-0.87	1.87
17	BnaC06g10240D	AT1G52600	Peptidase S24/S26A/S26B/S26C family protein	0.00	0.00	0.00	0.00	0.00	0.00	0.00	0.00	0.00	0.00	0.00	0.00	0.00	0.00	0.00	0.00
17	BnaC06g10250D	AT1G53790	F-box and associated interaction domains-containing protein	0.42	0.04	0.88	-0.12	0.72	-0.18	0.50	0.47	0.27	-0.79	0.22	0.57	0.18	-0.42	0.15	-0.20
17	BnaC06g10260D	AT1G53790	F-box and associated interaction domains-containing protein	-0.18	0.06	0.89	0.19	0.50	-0.02	0.92	0.33	-0.55	1.10	0.88	0.61	0.52	0.62	-0.05	0.22
17	BnaC06g10270D	AT1G53750	regulatory particle triple-A 1A (RPT1A)	1.22	0.98	0.51	-1.02	inf	1.58	0.00	-0.40	-2.54	inf	-0.18	3.30	-2.78	0.07	1.42	-0.73
17	BnaC06g10280D	AT1G53720	cyclophilin 59 (CYP59)	inf	0.00	0.00	0.00	0.00	0.00	0.00	0.00	0.00	0.00	0.00	0.00	0.00	inf	0.00	0.00
17	BnaC06g10290D	AT1G53750	regulatory particle triple-A 1A (RPT1A)	-0.54	-1.08	-0.68	0.21	-1.27	-0.97	0.24	-0.64	0.47	-0.40	-0.52	0.01	0.36	0.10	0.23	-0.19

17	BnaC06g10300D	AT1G53760	unknown protein	0.47	-0.97	-0.38	0.41	-0.19	0.43	0.84	0.10	-0.55	0.67	0.37	-0.03	-0.14	-0.11	-0.43	-0.27
17	BnaC06g10310D			0.00	0.00	0.00	0.00	0.00	0.00	0.00	inf	0.00	0.00	0.00	inf	0.00	0.00	0.00	0.00
17	BnaC06g10320D	AT1G53800	unknown protein	0.39	0.74	0.26	0.74	0.02	-0.12	1.52	0.40	-0.26	0.10	0.66	-0.37	-0.64	-0.25	0.82	0.39
17	BnaC06g10330D	AT5G38830	Cysteinyl-tRNA synthetase, class Ia family protein	0.00	0.00	inf	0.00	0.00	0.00	0.00	inf	0.00	0.00	0.00	0.00	0.00	0.00	0.00	0.00
17	BnaC06g10340D	AT1G53820	RING/U-box superfamily protein	-3.21	-0.41	0.81	inf	1.06	1.06	-0.56	-2.37	1.66	-1.10	-1.98	-0.58	0.39	0.73	0.00	0.00
17	BnaC06g10350D			0.00	0.00	0.00	0.00	0.00	0.00	0.00	0.00	0.00	0.00	0.00	0.00	0.00	0.00	0.00	0.00
17	BnaC06g10360D	AT1G53790	F-box and associated interaction domains-containing protein	0.00	0.00	inf	0.00	0.00	0.00	0.00	0.00	0.00	0.00	0.00	inf	0.00	0.00	0.00	0.00
17	BnaC06g10370D	AT1G53025	Ubiquitin-conjugating enzyme family protein	0.00	0.00	inf	0.00	0.00	inf	-1.61	-0.64	0.00	0.00	0.00	-0.20	inf	0.00	-0.37	-0.37
18	BnaC06g29120D	AT1G67950	RNA-binding (RRM/RBD/RNP motifs) family protein	0.79	0.36	0.15	-0.11	0.28	-0.18	-0.27	0.33	-0.33	-0.43	0.32	0.30	0.02	0.38	0.19	0.20
18	BnaC06g29130D	AT1G67940	non-intrinsic ABC protein 3 (NAP3)	-0.39	0.13	-0.09	-0.11	0.36	-0.53	0.49	-0.08	0.53	0.35	-0.38	-0.05	0.00	-0.22	0.37	0.00
18	BnaC06g29140D	AT1G67970	heat shock transcription factor A8 (HSFA8)	-0.68	0.21	0.51	-0.54	0.02	-0.11	0.39	-0.14	0.33	0.07	0.20	-0.49	-0.38	-0.57	-0.70	-0.22
18	BnaC06g29150D	AT1G67980	caffeoyl-CoA 3-O-methyltransferase (CCOAMT)	0.10	-2.66	-0.80	0.00	2.23	-0.17	inf	-0.95	1.02	inf	0.50	1.84	-0.81	-1.10	inf	inf
18	BnaC06g29160D	AT1G68010	hydroxypyruvate reductase (HPR)	-0.21	0.43	-0.01	-0.74	0.58	0.05	-0.64	0.15	0.58	0.02	-0.19	0.43	-0.47	-0.10	1.19	-0.11
18	BnaC06g29170D	AT1G68030	RING/FYVE/PHD zinc finger superfamily protein	-0.10	0.17	-0.09	0.14	0.49	-0.50	0.54	0.54	-0.11	0.21	0.26	0.03	-0.31	-0.44	-0.10	-0.65
18	BnaC06g29180D	AT1G68040	S-adenosyl-L-methionine-dependent methyltransferases superfamily protein	0.58	0.00	-0.61	inf	0.00	0.00	0.00	-0.41	0.84	0.00	1.95	-1.49	0.00	0.00	inf	inf
18	BnaC06g29190D	AT1G68060	microtubule-associated proteins 70-1 (MAP70-1)	-0.07	-0.36	0.12	-0.41	-0.39	-0.70	-0.29	0.13	-0.27	-0.39	-0.02	-0.01	0.50	-0.17	-0.23	-0.34
18	BnaC06g29200D	AT1G68070	Zinc finger, C3HC4 type (RING finger) family protein	-0.51	-0.54	0.18	0.30	0.15	-0.24	0.19	0.05	0.38	0.37	-0.09	0.34	0.32	0.03	0.36	-0.15
18	BnaC06g29210D	AT1G74770	zinc ion binding	0.00	-1.98	0.42	1.34	-0.64	-0.01	2.02	-1.80	0.39	-0.15	0.12	-1.70	-0.17	-1.19	0.50	-1.01
18	BnaC06g29220D			0.00	0.00	0.00	0.00	0.00	0.00	-1.09	0.00	0.00	0.00	0.00	0.00	0.00	0.00	0.00	0.00
18	BnaC06g29230D	AT1G68100	IAA-ALANINE RESISTANT 1 (IAR1)	1.28	0.38	0.48	-0.52	0.29	0.73	-0.72	0.93	0.15	0.05	-0.36	0.74	0.33	0.30	-0.26	-0.02
18	BnaC06g29240D	AT1G68110	ENTH/ANTH/VHS superfamily protein	-3.62	-1.43	-1.20	0.04	-0.84	-0.75	-0.47	-0.42	-0.10	0.30	0.39	1.32	1.73	-1.95	0.49	-0.41
18	BnaC06g29250D	AT1G65030	Transducin/WD40 repeat-like superfamily protein	inf	0.00	0.00	0.00	0.00	0.00	inf	0.00	inf	0.00	-0.39	0.65	0.00	0.00	0.00	0.00
18	BnaC06g29260D	AT1G65032	unknown protein	0.01	1.07	0.13	1.60	0.32	0.32	0.46	-0.76	-0.40	1.18	-0.43	-0.32	0.01	0.10	0.27	-0.72
18	BnaC06g29270D			0.00	2.17	0.20	-0.75	2.72	inf	0.00	-0.23	-0.64	1.08	-0.13	-1.95	0.65	0.01	0.97	0.00
18	BnaC06g29280D	AT1G65032	unknown protein	inf	0.00	0.00	0.00	0.00	0.00	0.00	inf	0.00	0.00	0.00	0.00	0.00	inf	0.00	0.00
18	BnaC06g29290D			0.00	0.00	0.00	0.00	0.00	0.00	inf	0.00	0.00	0.00	inf	0.00	0.00	0.00	0.00	0.00
18	BnaC06g29300D			0.00	0.00	0.00	0.00	0.00	0.00	0.00	0.00	0.00	0.00	0.00	0.00	0.00	0.00	0.00	0.00
18	BnaC06g29310D	AT1G68150	WRKY DNA-binding protein 9 (WRKY9)	0.00	0.00	0.93	0.00	0.00	0.00	0.00	inf	0.00	-1.06	0.00	1.38	0.00	0.00	0.00	0.00
18	BnaC06g29320D	AT1G68160	Protein of unknown function (DUF3755)	-0.70	-0.13	-0.07	0.14	-0.47	-0.09	-0.18	0.65	-0.30	-0.05	-0.13	-0.11	-0.09	-0.06	-0.14	-0.11
18	BnaC06g29330D	AT1G68180	RING/U-box superfamily protein	0.65	inf	1.66	0.00	0.00	0.00	0.00	-2.36	-1.42	inf	0.00	0.00	1.81	0.00	0.00	0.00
18	BnaC06g29340D	AT1G68190	B-box zinc finger family protein	0.00	0.00	0.00	0.00	0.00	0.00	0.00	0.00	0.00	0.00	0.00	0.00	0.00	0.00	0.00	0.00
18	BnaC06g29350D	AT1G68200	Zinc finger C-x8-C-x5-C-x3-H type family protein	1.35	1.72	1.74	-0.81	0.23	-1.36	-0.14	0.27	-1.22	0.03	0.48	0.31	-0.73	2.92	inf	inf
18	BnaC06g29360D	AT1G68210	pseudo-response regulator 6 (APRR6)	0.00	0.00	0.00	0.00	0.00	0.00	0.00	0.00	0.00	inf	inf	inf	0.00	0.00	0.00	0.00
18	BnaC06g29370D	AT1G68220	Protein of unknown function (DUF1218)	-0.46	0.11	0.08	-0.59	0.48	0.14	-0.42	0.20	-0.51	0.00	0.07	-0.01	0.38	-0.02	-0.18	0.21
18	BnaC06g29380D	AT1G68230	Reticulon family protein	0.33	0.34	-0.61	0.00	inf	inf	0.00	0.90	1.10	1.13	-0.27	-0.43	-0.70	-1.55	0.00	0.00
18	BnaC06g29390D			0.00	0.00	0.00	0.00	0.00	0.00	0.00	0.00	0.00	0.00	0.00	0.00	0.00	0.00	0.00	0.00
18	BnaC06g29400D	AT1G77520	O-methyltransferase family protein	0.00	0.59	0.00	0.00	5.27	1.29	0.00	-0.20	0.00	1.12	0.00	0.22	0.00	1.51	0.00	0.00
18	BnaC06g29410D	AT5G42090	Lung seven transmembrane receptor family protein	0.35	-0.02	0.19	-0.42	-0.03	0.21	-0.74	0.02	0.06	-0.18	-0.12	0.30	0.24	0.43	0.21	0.06
18	BnaC06g29420D	AT1G68260	Thioesterase superfamily protein	-2.02	0.81	0.74	-0.32	0.05	-0.90	-0.38	0.69	0.46	-0.28	0.40	-0.10	-0.16	0.22	-0.28	-0.21
18	BnaC06g29430D	AT1G68290	endonuclease 2 (ENDO 2)	1.20	-0.75	-0.47	0.45	0.43	0.90	0.46	0.05	0.57	0.00	-1.40	0.21	0.79	-1.03	0.08	0.91
18	BnaC06g29440D	AT1G68300	Adenine nucleotide alpha hydrolases-like superfamily protein	0.00	-0.43	0.06	0.91	0.30	0.15	0.84	0.20	-0.22	0.00	-0.22	0.26	0.64	-0.05	0.11	0.00
18	BnaC06g29450D	AT1G68310	FUNCTIONS IN: vacuolar sorting signal binding	0.70	0.18	0.40	0.09	0.27	0.05	0.18	0.12	-0.18	0.89	0.24	-0.58	0.10	-0.32	-0.01	0.38
19	BnaC08g04150D			0.00	0.00	0.00	0.00	0.00	0.00	0.00	0.00	0.00	0.00	0.00	0.00	0.00	0.00	0.00	0.00
19	BnaC08g04160D			0.00	0.00	0.00	0.00	0.00	0.00	0.00	0.00	0.00	0.00	0.00	0.00	0.00	0.00	0.00	0.00
19	BnaC08g04170D			0.00	0.00	0.00	0.00	0.00	0.00	0.00	0.00	0.00	0.00	0.00	0.00	0.00	0.00	0.00	0.00
19	BnaC08g04180D			0.00	0.00	0.00	0.00	0.00	0.00	0.00	0.00	0.00	0.00	0.00	0.00	0.00	0.00	0.00	0.00
19	BnaC08g04190D			0.00	0.00	0.00	0.00	0.00	0.00	0.00	0.00	0.00	0.00	0.00	0.00	0.00	0.00	0.00	0.00
19	BnaC08g04200D	AT2G07240	cysteine-type peptidases	0.00	0.00	0.00	0.00	0.00	0.00	0.00	0.00	0.00	0.00	0.00	0.00	0.00	0.00	0.00	0.00
19	BnaC08g04210D			0.00	0.00	0.00	0.00	0.00	0.00	0.00	0.00	0.00	0.00	0.00	0.00	0.00	0.00	0.00	0.00

19	BnaC08g04220D			0.00	0.00	0.00	0.00	0.00	0.00	0.00	0.00	0.00	0.00	0.00	0.00	0.00	0.00	0.00	0.00
19	BnaC08g04230D	AT4G04775	zinc ion binding	0.00	0.00	0.00	0.00	0.00	0.00	0.00	0.00	0.00	0.00	0.00	0.00	0.00	0.00	0.00	0.00
19	BnaC08g04240D	AT1G47750	peroxin 11A (PEX11A)	0.34	-0.23	-0.40	-0.87	1.08	0.15	-0.38	-0.61	0.60	1.18	0.26	1.61	0.51	-0.38	0.24	-0.23
19	BnaC08g04250D			0.00	0.00	0.00	0.00	0.00	0.00	0.00	0.00	0.00	0.00	0.00	0.00	0.00	0.00	0.00	0.00
19	BnaC08g04260D	AT1G47740	PPPDE putative thiol peptidase family protein	0.04	0.93	0.81	-0.73	-1.20	0.84	-0.41	0.54	-0.43	0.50	0.22	0.06	0.82	0.94	0.17	0.10
19	BnaC08g04270D	AT1G28710	Nucleotide-diphospho-sugar transferase family protein	0.00	0.00	1.97	0.00	0.00	0.00	0.00	inf	0.00	0.00	0.00	0.94	0.00	0.00	0.00	0.00
19	BnaC08g04280D	AT3G44770	Protein of unknown function (DUF626)	0.00	inf	0.00	0.00	0.00	0.00	0.00	0.00	0.00	0.00	0.00	0.00	0.00	0.00	0.00	0.00
19	BnaC08g04290D	AT1G47720	Organellar Single-stranded (OSB1)	-0.24	-0.30	0.16	-0.16	0.73	0.22	0.14	0.17	0.33	0.24	0.07	-0.41	-0.15	0.29	0.02	-0.22
19	BnaC08g04300D	AT1G03190	ULTRAVIOLET HYPERSENSITIVE 6 (UVH6)	0.00	0.00	0.00	0.00	0.00	0.00	0.00	0.00	0.00	0.00	0.00	0.00	0.00	0.00	0.00	0.00
19	BnaC08g04310D	AT2G05400	Ubiquitin-specific protease family C19-related protein	0.00	0.00	0.00	0.00	0.00	0.00	inf	0.00	0.00	0.00	0.00	0.00	0.00	0.00	0.00	0.00
19	BnaC08g04320D	AT4G31980	unknown protein	0.15	0.50	0.92	-0.78	-0.05	0.05	-2.25	0.84	-0.95	0.22	-0.96	0.83	0.34	-0.14	-0.47	-0.91
19	BnaC08g04330D			0.00	0.00	0.00	0.00	0.00	0.00	0.00	0.00	0.00	0.00	0.00	0.00	0.00	0.00	0.00	0.00
19	BnaC08g04340D	AT1G47670	Transmembrane amino acid transporter family protein	0.52	1.53	0.26	-0.35	0.25	-1.68	-0.91	0.03	-0.21	0.52	0.01	-0.07	0.81	-0.62	-0.13	-0.45
19	BnaC08g04350D	AT1G47655	Dof-type zinc finger DNA-binding family protein	0.00	0.00	0.00	0.00	0.00	0.00	0.00	0.00	0.00	0.00	0.00	0.00	0.00	0.00	0.00	0.00
19	BnaC08g04360D	AT5G45570	Ulp1 protease family protein	0.00	0.00	0.00	0.00	0.00	0.00	0.00	0.00	0.00	0.00	0.00	0.00	0.00	0.00	0.00	0.00
19	BnaC08g04370D			0.00	0.00	0.00	0.00	0.00	0.00	0.00	0.00	0.00	0.00	0.00	0.00	0.00	0.00	0.00	0.00
19	BnaC08g04380D	AT3G53810	Concanavalin A-like lectin protein kinase family protein	0.00	0.00	0.00	0.00	0.00	0.00	0.00	0.00	0.00	0.00	0.00	0.00	0.00	inf	0.00	0.00
19	BnaC08g04390D			0.00	0.00	0.00	0.00	0.00	0.00	0.00	0.00	0.00	0.00	0.00	0.00	0.00	0.00	0.00	0.00
19	BnaC08g04400D	AT1G80070	ABNORMAL SUSPENSOR 2 (SUS2)	1.61	-0.18	-0.15	0.19	0.95	0.70	1.72	0.09	1.21	1.70	-0.30	-0.82	0.18	0.25	-0.42	0.06
19	BnaC08g04410D	AT1G47480	alpha/beta-Hydrolases superfamily protein	0.00	0.00	0.00	0.00	0.00	0.00	0.00	0.00	0.00	0.00	0.00	0.00	0.00	0.00	0.00	0.00
19	BnaC08g04420D	AT1G47480	alpha/beta-Hydrolases superfamily protein	0.00	0.00	0.00	0.00	0.00	0.00	0.00	0.00	0.00	0.00	0.00	0.00	0.00	0.00	0.00	0.00
19	BnaC08g04430D			0.00	0.00	0.00	0.00	0.00	0.00	0.00	0.00	0.00	0.00	0.00	0.00	0.00	0.00	0.00	0.00
19	BnaC08g04440D			0.00	0.00	0.00	0.00	0.00	0.00	0.00	0.00	0.00	0.00	0.00	0.00	0.00	0.00	0.00	0.00
19	BnaC08g04450D	AT5G05200	Protein kinase superfamily protein	inf	0.00	inf	0.00	inf	0.00	0.00	0.00	inf	0.00	0.00	0.00	0.00	0.00	0.00	0.00
19	BnaC08g04460D			0.00	0.00	0.00	0.00	0.00	0.00	0.00	0.00	0.00	0.00	0.00	0.00	0.00	0.00	0.00	0.00
19	BnaC08g04470D	AT1G47420	succinate dehydrogenase 5 (SDH5)	0.12	-0.06	-0.05	0.49	-0.05	-0.01	0.60	0.18	-0.27	-0.21	-0.04	0.06	-0.36	-0.11	-0.11	-0.08
19	BnaC08g04480D	AT1G47410	unknown protein	0.00	0.00	0.00	0.00	0.00	0.00	0.00	0.00	0.00	0.00	0.00	0.00	0.00	0.00	0.00	0.00
19	BnaC08g04490D			0.00	0.00	0.00	0.00	0.00	0.00	0.00	0.00	0.00	0.00	0.00	0.00	0.00	0.00	0.00	0.00
19	BnaC08g04500D			0.00	0.00	0.00	0.00	0.00	0.00	inf	0.00	0.00	0.00	0.00	0.00	0.00	0.00	0.00	0.00
19	BnaC08g04510D	AT3G02040	senescence-related gene 3 (SRG3)	0.49	1.51	0.00	-2.24	0.70	-0.10	-1.39	-0.50	0.20	-4.10	-0.59	0.34	0.83	1.74	0.73	-0.27
19	BnaC08g04520D	AT3G02030	transferases, transferring acyl groups other than amino-acyl groups	0.00	0.00	inf	0.00	0.00	0.00	0.00	0.00	0.00	0.00	0.00	inf	0.00	0.00	0.00	0.00
19	BnaC08g04530D			0.00	0.00	0.00	0.00	0.00	0.00	0.00	0.00	0.00	0.00	0.00	0.00	0.00	0.00	0.00	0.00
19	BnaC08g04540D	AT1G47380	Protein phosphatase 2C family protein	0.48	0.17	-0.24	-0.73	0.17	0.03	-0.05	-0.10	-0.14	0.10	0.06	0.06	0.11	0.26	-0.09	-0.18
19	BnaC08g04550D	AT1G47340	F-box and associated interaction domains-containing protein	0.00	-3.36	1.47	inf	0.00	-2.40	1.27	1.12	0.00	0.00	-0.24	-0.04	0.48	0.00	0.36	-1.54
19	BnaC08g04560D	AT1G47290	3beta-hydroxysteroid-dehydrogenase/decarboxylase isoform 1 (3BETAHSD/D1)	0.00	0.00	0.00	0.00	0.00	0.00	0.00	0.00	0.00	0.00	0.00	0.00	0.00	0.00	0.00	0.00
19	BnaC08g04570D	AT1G47280	unknown protein	0.00	0.00	0.00	inf	inf	-0.67	0.00	0.00	0.00	0.00	0.43	inf	0.00	0.00	-0.93	0.00
19	BnaC08g04580D	AT1G47278	unknown protein	0.00	inf	0.00	0.00	0.00	0.00	0.00	0.00	inf	inf	-0.53	-0.78	-1.05	0.00	0.00	0.00
19	BnaC08g04590D	AT5G38820	Transmembrane amino acid transporter family protein	-0.12	-0.54	1.66	-0.33	-1.41	-0.07	-1.44	0.52	-0.63	0.25	0.19	0.28	1.57	-1.29	-0.12	-0.52
19	BnaC08g04600D	AT5G38830	Cysteinyl-tRNA synthetase, class Ia family protein	-1.41	-1.13	0.07	-1.03	1.37	0.88	0.13	0.56	0.55	-0.18	-1.60	-0.20	-1.41	-0.39	-1.53	0.92
19	BnaC08g04610D	AT2G42550	Protein kinase superfamily protein	0.00	0.00	2.00	inf	0.00	inf	inf	1.21	0.00	inf	1.30	-0.02	0.00	0.00	0.00	inf
19	BnaC08g04620D	AT1G47260	gamma carbonic anhydrase 2 (GAMMA CA2)	0.36	0.35	0.09	-0.04	0.37	0.02	0.11	0.18	-0.01	-0.16	0.26	0.20	-0.18	-0.10	-0.09	-0.15
19	BnaC08g04630D	AT1G47250	20S proteasome alpha subunit F2 (PAF2)	-0.35	0.14	-0.22	-0.34	0.22	0.17	-0.27	0.02	-0.24	0.46	-0.18	0.54	-0.23	-0.11	0.22	-0.32
19	BnaC08g04640D	AT1G47240	NRAMP metal ion transporter 2 (NRAMP2)	0.13	0.47	0.29	0.38	0.57	-0.03	-0.11	0.10	-0.46	0.10	0.09	0.42	0.17	-0.04	0.21	-0.16
19	BnaC08g04650D	AT1G47230	CYCLIN A3	-0.14	0.66	0.18	-0.50	-0.36	1.71	-1.07	-0.40	-0.10	-0.58	-0.23	-0.58	-1.16	0.13	0.03	-0.31
19	BnaC08g04660D	AT1G47210	cyclin-dependent protein kinase 3	0.63	1.53	0.35	0.00	0.86	0.74	0.77	0.69	-0.57	-0.13	-0.04	0.01	-0.92	0.08	0.17	-0.36
19	BnaC08g04670D			0.00	0.00	-0.93	0.00	0.00	inf	0.00	0.00	inf	0.00	0.00	inf	-0.69	0.00	0.00	0.00
19	BnaC08g04680D			0.00	0.00	0.00	0.00	0.00	0.00	0.00	0.00	0.00	0.00	0.00	0.00	0.00	0.00	0.00	0.00
19	BnaC08g04690D			0.00	0.00	-0.34	0.00	0.00	0.00	0.00	0.00	0.00	0.00	0.00	0.00	0.00	0.00	0.00	inf

19	BnaC08g04700D	AT1G47200 WPP domain protein 2 (WPP2)	0.49	0.61	0.24	-0.25	-0.19	-0.07	-0.26	0.31	-0.27	-0.05	0.06	0.21	-0.41	1.07	0.00	0.11
19	BnaC08g04710D		0.00	0.00	0.00	0.00	0.00	2.08	0.00	0.00	0.00	0.00	0.00	0.03	1.60	0.00	0.00	0.00
19	BnaC08g04720D	AT5G37320 Protein of unknown function (DUF674)	0.00	0.00	0.00	0.00	0.00	0.00	0.00	0.00	0.00	0.00	0.00	0.00	0.00	0.00	0.00	0.00
19	BnaC08g04730D	AT5G37320 Protein of unknown function (DUF674)	0.00	0.00	0.00	0.00	0.00	0.00	0.00	0.00	0.00	0.00	0.00	0.00	0.00	0.00	0.00	0.00
19	BnaC08g04740D	AT5G01150 Protein of unknown function (DUF674)	0.00	0.00	0.00	0.00	0.00	0.00	0.00	0.00	0.00	0.00	0.00	0.00	0.00	0.00	0.00	0.00
19	BnaC08g04750D	AT1G45976 S-ribonuclease binding protein 1 (SBP1)	-0.14	0.43	0.24	0.33	0.59	0.06	0.02	0.68	-0.38	0.75	0.50	0.32	0.64	-0.08	0.43	-0.41
19	BnaC08g04760D		0.00	0.00	0.00	0.00	0.00	inf	0.00	0.00	0.00	0.00	0.00	0.00	0.00	0.00	0.00	0.00
19	BnaC08g04770D		0.00	0.00	inf	0.00	inf	0.00	0.00	0.00	0.00	inf	0.00	0.00	0.00	0.00	0.00	0.00
21	BnaC09g31320D		0.00	0.00	0.00	0.00	0.00	0.00	0.00	0.00	0.00	0.00	0.00	0.00	0.00	0.00	0.00	0.00
21	BnaC09g31330D	AT5G54840 SGP1	0.55	-1.22	-0.75	0.76	0.48	0.07	0.66	-0.53	0.03	0.07	-0.29	0.29	0.37	-0.29	0.34	-0.34
21	BnaC09g31340D	AT1G56050 GTP-binding protein-related	0.00	0.00	0.00	0.00	0.00	0.00	0.00	0.00	0.00	0.00	0.00	0.00	0.00	0.00	0.00	0.00
21	BnaC09g31350D		0.00	0.00	0.00	0.00	0.00	0.00	0.00	0.00	0.00	0.00	0.00	0.00	0.00	inf	0.00	0.00
21	BnaC09g31360D	AT5G54850 unknown protein	1.11	0.86	0.41	0.45	-0.81	-2.23	1.00	0.67	0.66	-0.11	0.38	0.77	0.06	-0.70	-0.13	-0.46
21	BnaC09g31370D	AT5G54855 Pollen Ole e 1 allergen and extensin family protein	0.43	0.27	0.04	0.28	0.04	0.47	0.34	0.34	-0.29	0.47	0.29	0.38	0.20	0.07	0.22	-0.31
21	BnaC09g31380D	AT5G54860 Major facilitator superfamily protein	0.27	-0.21	-0.02	0.10	0.13	0.26	0.46	0.30	-0.03	0.15	0.22	0.29	-0.50	-0.60	0.19	-0.83
21	BnaC09g31390D	AT5G54870 unknown protein	2.84	-1.74	-0.84	0.17	0.24	1.25	-0.19	-1.05	1.07	0.36	0.12	0.79	1.67	-2.34	0.03	-0.61
21	BnaC09g31400D	AT5G54900 RNA-binding protein 45A (RBP45A)	0.30	-0.12	0.33	0.27	0.41	0.39	0.21	0.37	-0.38	-0.15	-0.11	0.41	0.22	-0.02	0.11	-0.01
21	BnaC09g31410D		0.00	0.00	0.00	0.00	0.00	0.00	0.00	0.00	0.00	0.00	0.00	0.00	0.00	0.00	0.00	0.00
21	BnaC09g31420D	AT5G54930 AT hook motif-containing protein	-0.02	0.49	0.25	-0.22	0.19	0.22	-0.15	0.39	-0.35	-0.06	-0.21	0.43	-0.07	-0.54	0.06	-0.11
21	BnaC09g31430D	AT5G54940 Translation initiation factor SUI1 family protein	0.83	-1.38	1.31	0.00	-1.27	0.67	inf	0.00	0.00	-0.80	0.00	0.42	1.01	-1.24	0.00	-2.32
21	BnaC09g31440D		0.00	0.00	0.00	0.00	0.00	0.00	0.00	0.00	0.00	0.00	0.00	0.00	0.00	0.00	inf	0.00
21	BnaC09g31450D	AT4G26970 aconitase 2 (ACO2)	0.00	0.00	0.00	0.00	0.00	0.00	0.00	0.00	0.00	0.00	0.00	0.00	0.00	0.00	0.00	0.00
21	BnaC09g31460D	AT5G54960 pyruvate decarboxylase-2 (PDC2)	0.51	-0.43	-0.23	0.00	0.34	0.51	-0.64	-0.19	-0.34	0.13	0.06	0.50	0.65	0.74	0.25	0.06
21	BnaC09g31470D		0.00	0.00	0.00	inf	0.00	0.00	0.00	0.00	0.00	0.00	0.00	0.00	inf	0.00	0.00	0.00
21	BnaC09g31480D		0.00	0.00	0.00	0.00	0.00	0.00	0.00	0.00	0.00	0.00	0.00	0.00	0.00	0.00	0.00	0.00
21	BnaC09g31490D		0.00	0.00	0.00	0.00	0.00	0.00	0.00	0.00	0.00	0.00	0.00	0.00	0.00	0.00	0.00	0.00
21	BnaC09g31500D		0.00	0.00	0.00	0.00	0.00	0.00	0.00	0.00	0.00	0.00	inf	inf	0.00	0.00	0.00	0.00
21	BnaC09g31510D		0.00	0.00	0.00	0.00	0.00	0.00	0.00	0.00	0.00	0.00	0.00	0.00	0.00	0.00	0.00	0.00
21	BnaC09g31520D		0.00	0.00	0.00	0.00	0.00	0.00	0.00	0.00	0.00	0.00	0.00	0.00	0.00	0.00	0.00	0.00
21	BnaC09g31530D	AT2G05140 phosphoribosylaminoimidazole carboxylase family protein / AIR carboxylase family protein	0.00	0.00	0.00	0.00	0.00	0.00	0.00	0.00	0.00	0.00	0.00	0.00	0.00	0.00	0.00	0.00
21	BnaC09g31540D	AT5G54980 Uncharacterised protein family (UPF0497)	-0.18	-1.10	-0.05	0.60	0.37	0.17	0.57	0.25	-0.55	0.02	-0.05	-0.01	0.22	-0.43	-0.06	-0.71
21	BnaC09g31550D	AT4G11770 Galactose oxidase/kelch repeat superfamily protein	0.00	0.00	0.00	0.00	0.00	0.00	0.00	0.00	0.00	0.00	inf	0.00	0.00	0.00	0.00	0.00
21	BnaC09g31560D	AT4G26930 myb domain protein 97 (MYB97)	0.00	0.00	1.45	0.00	0.00	0.00	0.00	1.28	0.00	inf	0.61	1.34	0.00	0.00	0.00	0.00
