# Supplementary material for: Genome-Wide Association and Transcriptome Analyses Reveal Candidate Genes Underlying Yield-determining Traits in Brassica napus
Source: Front Plant Sci. 2017 Feb 15;8:206. doi: 10.3389/fpls.2017.00206 (PMC5309214; doi:10.3389/fpls.2017.00206)
Supplement: Supplementary file 9 [file Image1.PDF]

Supplementary Material

Genome-Wide Association and Transcriptome Analyses Reveal Candidate Genes Underlying Yield-determining Traits in *Brassica napus*

Kun Lu[1†*], Liu Peng[1,2†], Chao Zhang[1,3], Junhua Lu[1], Bo Yang[1], Zhongchun Xiao[1], Ying Liang[1], Xingfu Xu[1], Cunmin Qu[1], Kai Zhang[1], Liezhao Liu[1], Qinlong Zhu[4], Minglian Fu[5], Xiaoyan Yuan[5], Jiana Li[1*]

*** Correspondence:**

Kun Lu: drlukun@swu.edu.cn
Jiana Li: ljn1950@swu.edu.cn

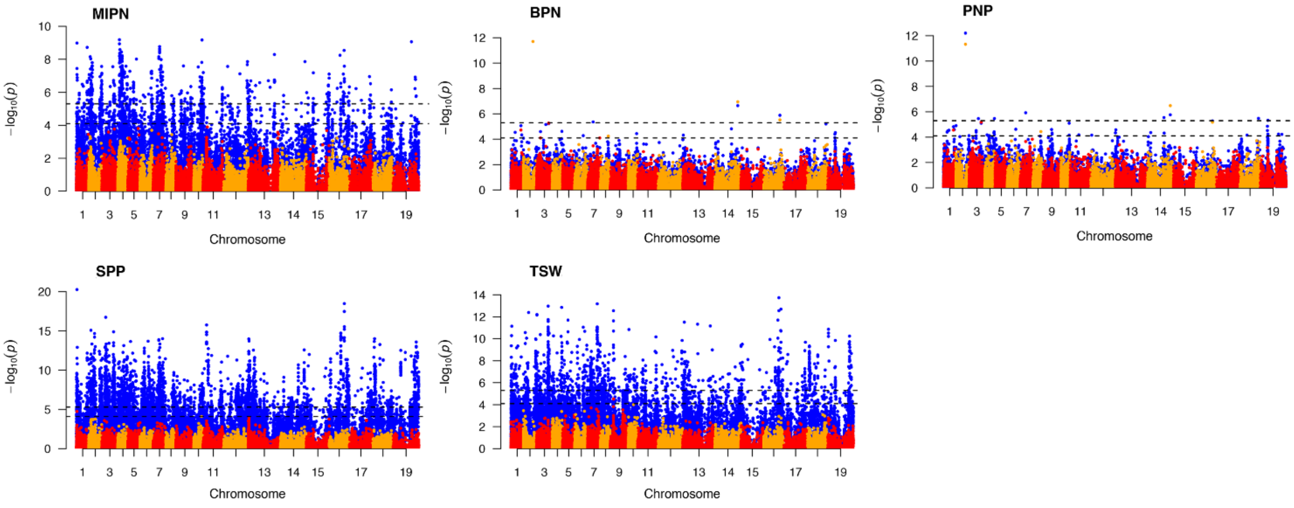

Supplementary Figure S1. Manhattan plots of GWASs for YDTs in E1.

MIPN, main inflorescence pod number; BPN, branch pod number; PNP, pod number per plant; SPP, seed number per pod; TSW, thousand seed weight. The dashed horizontal lines depict the suggestive ($-\log_{10}(P)$ = 4.11) and significance thresholds ($-\log_{10}(P)$ = 5.41). GWAS loci identified with the mixed linear model (MLM) are indicated with light blue dots, those identified with the general linear model (GLM) are indicated with red and yellow dots. E1 refers to plants grown in Chongqing (CQ, 29 º45' N, 106 º22' E, 238.57 m) during the 2012–13 growing seasons.
