# Supplementary material for: Genome-Wide Association and Transcriptome Analyses Reveal Candidate Genes Underlying Yield-determining Traits in Brassica napus
Source: Front Plant Sci. 2017 Feb 15;8:206. doi: 10.3389/fpls.2017.00206 (PMC5309214; doi:10.3389/fpls.2017.00206)
Supplement: Supplementary file 11 [file Image3.PDF]

Supplementary Material

Genome-Wide Association and Transcriptome Analyses Reveal Candidate Genes Underlying Yield-determining Traits in *Brassica napus*

Kun Lu[1†*], Liu Peng[1,2†], Chao Zhang[1,3], Junhua Lu[1], Bo Yang[1], Zhongchun Xiao[1], Ying Liang[1], Xingfu Xu[1], Cunmin Qu[1], Kai Zhang[1], Liezhao Liu[1], Qinlong Zhu[4], Minglian Fu[5], Xiaoyan Yuan[5], Jiana Li[1*]

*** Correspondence:**

Kun Lu: drlukun@swu.edu.cn
Jiana Li: ljn1950@swu.edu.cn

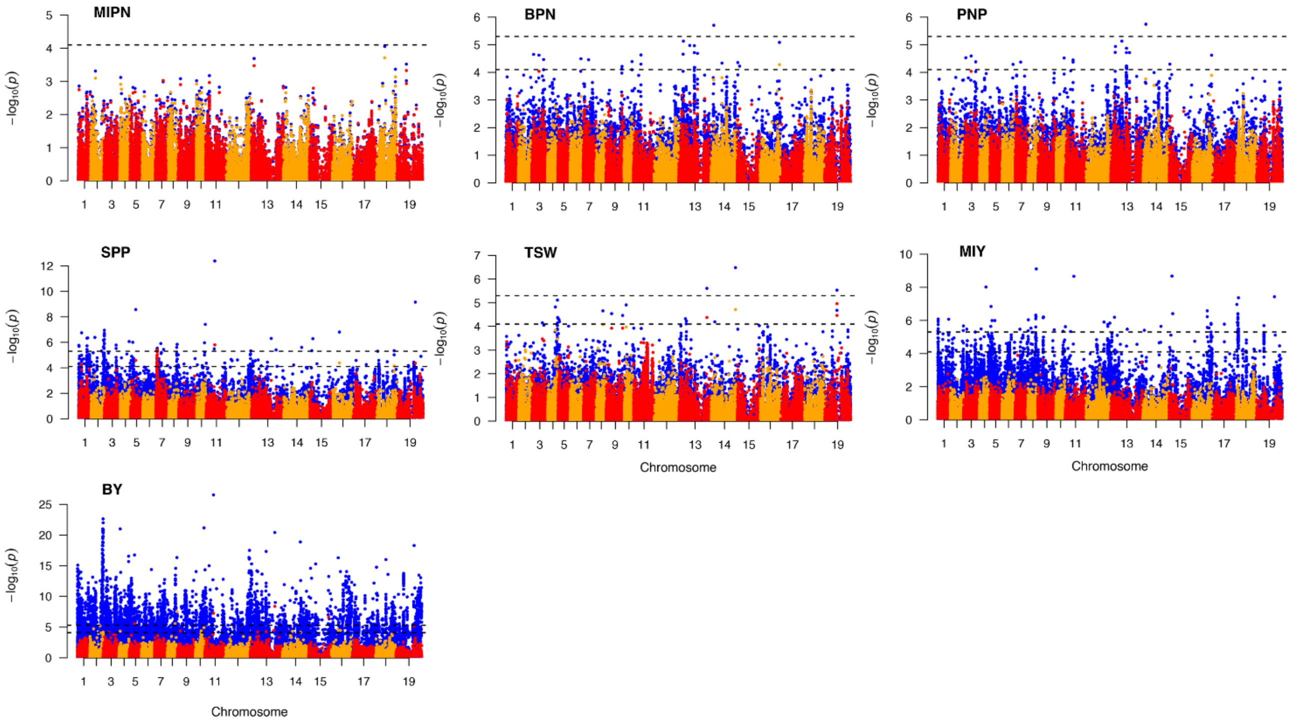

Supplementary Figure S3. Manhattan plots of GWASs for YDTs in E3.

MIPN, main inflorescence pod number; BPN, branch pod number; PNP, pod number per plant; SPP, seed number per pod; TSW, thousand seed weight; MIY, main inflorescence yield; BY, branch yield. The dashed horizontal lines depict the suggestive ($-\log_{10}(P)$ = 4.11) and significance thresholds ($-\log_{10}(P)$ = 5.41). GWAS loci identified with the mixed linear model (MLM) are indicated with light blue dots, those identified with the general linear model (GLM) are indicated with red and

yellow dots. E3 refers to plants grown in Yunnan (YN; 23 º 43' N, 100 º 02' E, 1819.50 m) in the 2013–14 growing season.
