# Supplementary material for: Genome-Wide Association and Transcriptome Analyses Reveal Candidate Genes Underlying Yield-determining Traits in Brassica napus
Source: Front Plant Sci. 2017 Feb 15;8:206. doi: 10.3389/fpls.2017.00206 (PMC5309214; doi:10.3389/fpls.2017.00206)
Supplement: Supplementary file 14 [file Image6.PDF]

Supplementary Material

Genome-Wide Association and Transcriptome Analyses Reveal Candidate Genes Underlying Yield-determining Traits in *Brassica napus*

Kun Lu[1†*], Liu Peng[1,2†], Chao Zhang[1,3], Junhua Lu[1], Bo Yang[1], Zhongchun Xiao[1], Ying Liang[1], Xingfu Xu[1], Cunmin Qu[1], Kai Zhang[1], Liezhao Liu[1], Qinlong Zhu[4], Minglian Fu[5], Xiaoyan Yuan[5], Jiana Li[1*]

*** Correspondence:**

Kun Lu: drlukun@swu.edu.cn
Jiana Li: ljn1950@swu.edu.cn

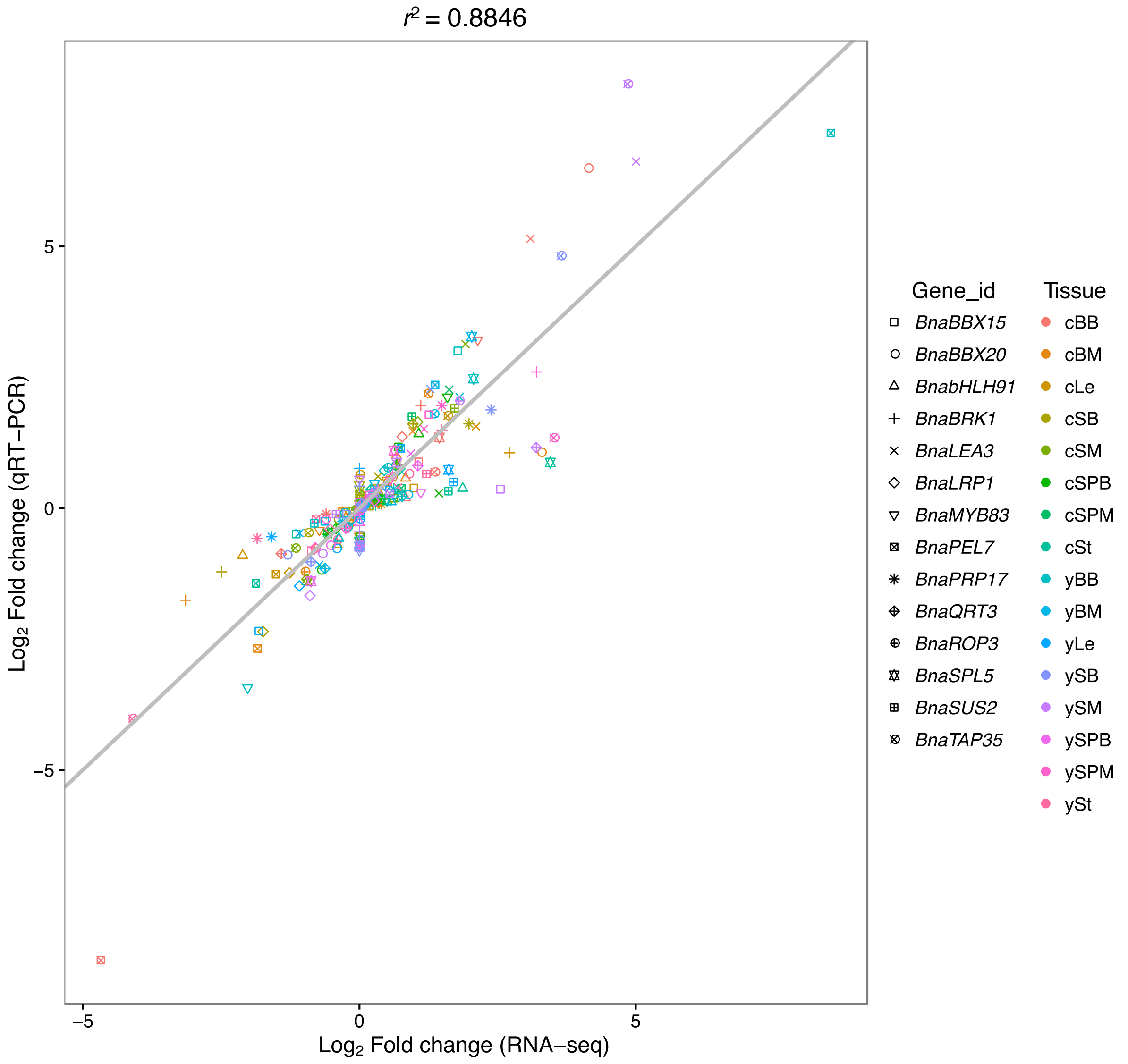

Supplementary Figure S6. Comparison of gene expression patterns of 14 candidate genes obtained by RNA-seq and qRT-PCR methods.

Fold-changes of 14 candidate genes within HBs were calculated in eight tissues from plants harvested at Chongqing (CQ) and Yunnan (YN). A high Pearson's correlation coefficient (PCC) (r^2 = 0.8846) was observed between the expression patterns of 14 selected genes determined by RNA-seq and qRT-PCR approaches. First letters in the tissue names (c and y) represent cultivation regions Chongqing (CQ) and Yunnan (YN), respectively. St, stems; Le, leaves; BM, buds on the main inflorescence; BB, buds on the primary branch; SPM, silique pericarps on the main inflorescence; SPB, silique pericarps on the primary branch; SM, seeds harvested 20 days after flowering on the main inflorescence; SB, seeds harvested 20 days after flowering on the primary branch.
