# Supplementary material for: Genome-Wide Association and Transcriptome Analyses Reveal Candidate Genes Underlying Yield-determining Traits in Brassica napus
Source: Front Plant Sci. 2017 Feb 15;8:206. doi: 10.3389/fpls.2017.00206 (PMC5309214; doi:10.3389/fpls.2017.00206)
Supplement: Supplementary file 15 [file Image7.PDF]

Supplementary Material

Genome-Wide Association and Transcriptome Analyses Reveal Candidate Genes Underlying Yield-determining Traits in *Brassica napus*

Kun Lu[1†*], Liu Peng[1,2†], Chao Zhang[1,3], Junhua Lu[1], Bo Yang[1], Zhongchun Xiao[1], Ying Liang[1], Xingfu Xu[1], Cunmin Qu[1], Kai Zhang[1], Liezhao Liu[1], Qinlong Zhu[4], Minglian Fu[5], Xiaoyan Yuan[5], Jiana Li[1*]

*** Correspondence:**

Kun Lu: drlukun@swu.edu.cn
Jiana Li: ljn1950@swu.edu.cn

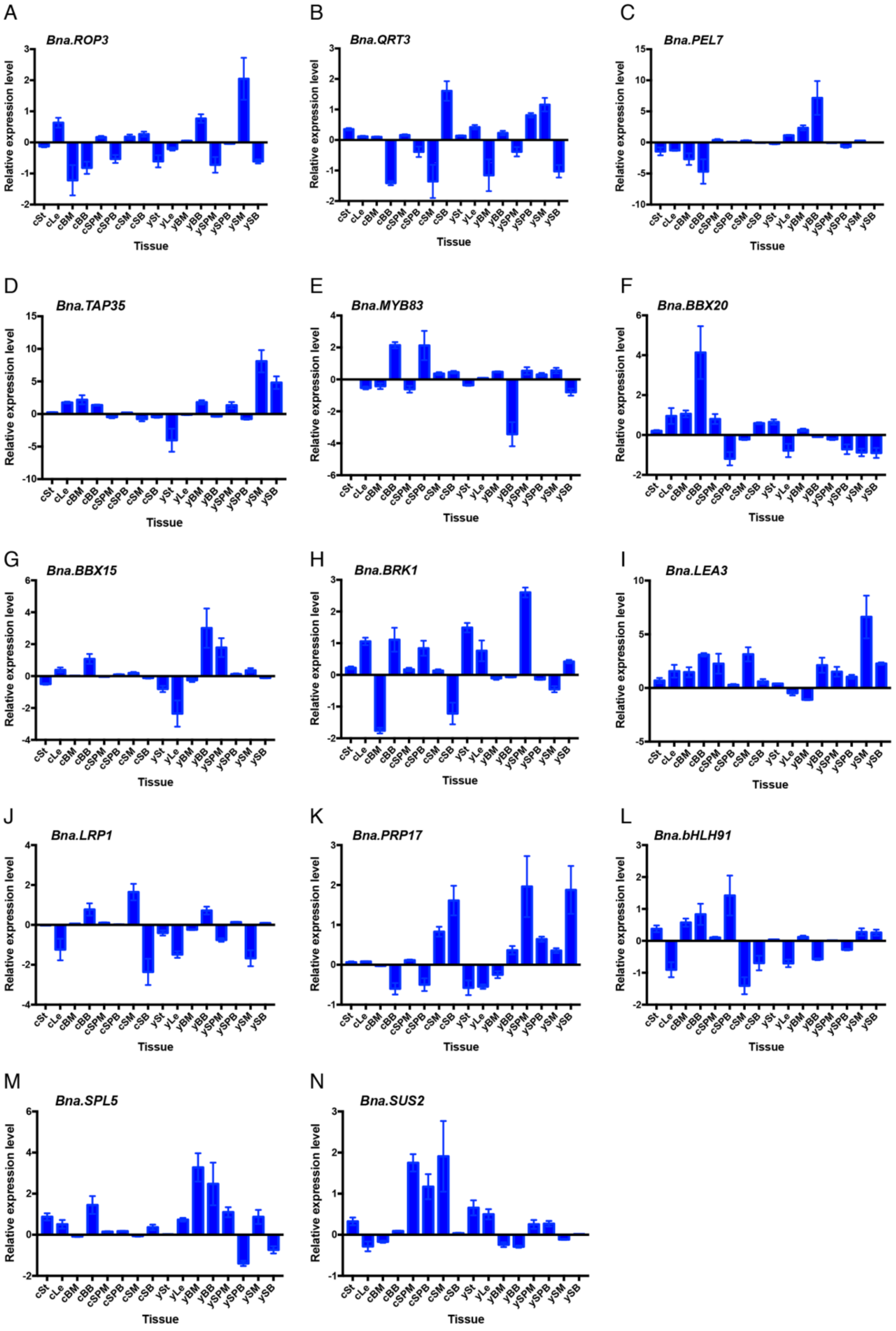
A
Bna.ROP3
B
Bna.QRT3
C
Bna.PEL7
D
Bna.TAP35
E
Bna.MYB83
F
Bna.BBX20
G
Bna.BBX15
H
Bna.BRK1
I
Bna.LEA3
J
Bna.LRP1
K
Bna.PRP17
L
Bna.bHLH91
M
Bna.SPL5
N
Bna.SUS2
Relative expression level
Tissue
cSt cLe cBM cBB cSPM cSPB cSM cSB ySt yLe yBM yBB ySPM ySPB ySM ySB

Supplementary Figure S7. Expression patterns of 14 differentially expressed genes (DEGs) determined by qRT-PCR.

First letters in the tissue names (c and y) represent cultivation regions Chongqing (CQ) and Yunnan (YN), respectively. St, stems; Le, leaves; BM, buds on the main inflorescence; BB, buds on the primary branch; SPM, silique pericarps on the main inflorescence; SPB, silique pericarps on the primary branch; SM, seeds harvested 20 days after flowering on the main inflorescence; SB, seeds harvested 20 days after flowering on the primary branch. Relative expression levels of DEGs represent the average ± SD of two biological replicates with three technical replicates per sample.
